# Algorithm-driven Artifacts in median polish summarization of Microarray data

**DOI:** 10.1186/1471-2105-11-553

**Published:** 2010-11-11

**Authors:** Federico M Giorgi, Anthony M Bolger, Marc Lohse, Bjoern Usadel

**Affiliations:** 1 Max Planck Institute of Molecular Plant Physiology, Am Muehlenberg 1, 14476 Golm - Germany

## Abstract

**Background:**

High-throughput measurement of transcript intensities using Affymetrix type oligonucleotide microarrays has produced a massive quantity of data during the last decade. Different preprocessing techniques exist to convert the raw signal intensities measured by these chips into gene expression estimates. Although these techniques have been widely benchmarked in the context of differential gene expression analysis, there are only few examples where their performance has been assessed in respect to coexpression-based studies such as sample classification.

**Results:**

In the present paper we benchmark the three most used normalization procedures (MAS5, RMA and GCRMA) in the context of inter-array correlation analysis, confirming and extending the finding that RMA and GCRMA consistently overestimate sample similarity upon normalization. We determine that median polish summarization is responsible for generating a large proportion of these over-similarity artifacts. Furthermore, we show that most affected probesets show also internal signal disagreement, and tend to be composed by individual probes hitting different gene transcripts. We finally provide a correction to the RMA/GCRMA summarization procedure that massively reduces inter-array correlation artifacts, without affecting the detection of differentially expressed genes.

**Conclusions:**

We propose tRMA as a modification of RMA to normalize microarray experiments for correlation-based analysis.

## Background

High density oligonucleotide microarrays are widely used in many areas of biological research for quantitative, high-throughput measurements of gene expression. Although ultra-deep sequencing techniques promise to replace them in the near future [1], it would be a mistake to ignore the biological importance of the massive quantity of data already produced through this platform. Publicly available databases alone store a huge (and growing) quantity of microarray experiments (e.g. 338947 samples in Gene Expression Omnibus [2] and 251711 in ArrayExpress [3]), comprising hundreds of different species.

Among microarrays, the single-channel Affymetrix GeneChip platform [4] is by far the most popular (for instance, in Gene Expression Omnibus they represent 97.9% of all arrays available for Arabidopsis thaliana, and 99.0% for Homo sapiens). In this technology each transcript is typically measured by a set of 11-20 pairs of 25 bases-long probes, collectively referred to as "probeset".

For every "perfect match" probe (PM), Affymetrix chips contain a "mismatch" counterpart (MM), with a single nucleotide change in the middle of the PM probe sequence. The role of MM probes, located adjacent to the respective PM, is to measure probe-specific background signal associated to any perfect-match signal intensity.

In general, the process of obtaining a single gene expression value out of raw probe intensity measurements is called "microarray preprocessing". Three steps are usually required: background correction, normalization and summarization. Many different methods or combinations of methods were proposed over the years [5,6].

The most popular manufacturer-provided method, MAS5 [7], uses a scale normalization approach, then corrects the background by subtracting the mean intensity of the lowest 2% spots in every microarray region, and then MM intensities from the respective PM ones. Wherever MM intensity is higher than a PM one, in order to avoid negative signal intensities typical of the MAS5 predecessor, MAS4 [4,8], MAS5 replaces the MM signal with an "idealized mismatch" value (IM) derived from other values in the same probeset. To extract final probeset intensities, MAS5 calculates a robust average (Tukey's biweight) of all the contained probes.

Many alternative techniques have challenged MAS5 supremacy for preprocessing. Being a single-array technique, MAS5 doesn't model probes' behaviour across different samples, and therefore suffers from high variance and is theoretically less robust than multi-array algorithms [9,10].

Two of the most popular multi-array normalization techniques are RMA [9] and GCRMA [11]. RMA doesn't use information contained in MM probes, and calculates background signal by performing a modelled global correction of all PM intensities. Then it applies a quantile normalization step and a median polish summarization, which accounts for probe intensities over multiple arrays. GCRMA applies the same normalization and summarization steps as RMA, but it differs in the background correction method, which is based on the probe sequence. Other multi-array methods which don't discard MM intensities exist, one of them being dChip [12]. However, in the present paper we will focus on RMA, GCRMA and MAS5, which are possibly the most popular microarray normalization methods [13,14]. Their popularity is illustrated by the fact that they are the most popular normalization techniques in online databases [15].

Most benchmarks have tried to assess the quality of different preprocessing methods in differential gene expression scenarios, the original purpose for which microarrays were developed [16]. To do so, golden set spike-in samples were used, with known concentrations of transcripts [17,6], or Real Time PCR measurements were performed for comparison [18]. The outcome of these benchmarks has not identified any technique as the top performer, although single-array techniques such as MAS5 have been outperformed by multi-array ones such as RMA [9,18,19].

However, many different approaches to biological investigation have relied on microarrays, ranging from gene and sample clustering [20] to gene-gene network reverse-engineering [21], from sample classification [22] to global transcript models [23]. The field of microarray data correlation and clustering based on the principle of coexpression has developed at a quite considerable pace [24]; despite this, the effects of preprocessing on coexpression analyses have been generally overlooked, with a few exceptions. [25] used bacterial operons to validate the different normalization techniques for correlation analysis and concluded that a combination of different methods works best. On the other hand, [26] have pointed out how the use of the multi-array techniques RMA and GCRMA can yield inter-array correlation artifacts and generally lower quality networks than the older MAS5. In particular, a specific step in GCRMA background correction (the gene-specific binding correction, or GSB) has been identified as partially responsible for the spurious correlations generated by GCRMA. Notably however, the correction of this step is not sufficient to remove all artifact effects, and no explanation was provided for artifacts produced by RMA.

In the present paper, we extend the analysis performed by [26], aiming to shed more light on the behaviour of multi-array techniques specifically in the context of inter-array correlation. We will describe the characteristics of probesets which induce these artifacts and provide both a mathematical and a biological explanation for the phenomenon. Finally, we introduce a slightly changed version of the RMA code which massively reduces inter-array correlation artifacts, while retaining RMA features in the context of differential gene expression analysis.

## Results

### Multi-array preprocessing effects

**Figure 1:**
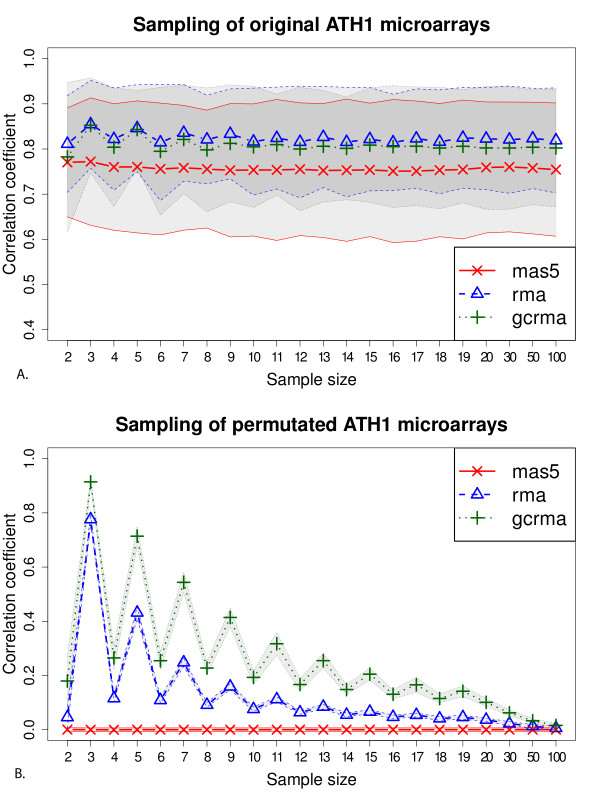
**Inter-array similarity calculated on the Arabidopsis dataset normalized by RMA, GCRMA and MAS5**. 1000 groups of arrays for each sample size were selected, and then the averages and standard deviations of inter-array Spearman correlation coefficients were calculated. The averages are reproduced as symbols which are connected by a broken line and averages plus minus one standard deviation are shown as shaded areas bordered by a solid line of the same color. Values for MAS5 are shown in red, RMA in blue and GCRMA in green. A) real samples. B) samples with their raw signal intensities internally permutated.

In order to compare the behaviour of three of the most popular microarray preprocessing techniques (MAS5, RMA and GCRMA), Lim and colleagues [26], tested these on a single dataset of 10 microarrays hybridized with human samples. We extended this analysis on a considerably larger Arabidopsis thaliana dataset comprising 3707 microarrays, selecting different sample sizes (see Material and Methods), according to the realistic size of a single experiment dataset (2 to 100 samples). First, we calculated inter-array correlations on randomly selected groups of original arrays (Figure 1A). The plots show us that many genes' relative expression will remain constant across different treatments and genotypes, indicating a certain robustness of Arabidopsis' genetic machinery in varying environmental conditions and other perturbations (e.g. gene knock-outs). The sample size doesn't seem to influence the high correlation between arrays, although some evident oscillations could be detected for RMA and GCRMA at lower sample sizes. The comparison of the three preprocessing methods shows that RMA and GCRMA yield somewhat more similar microarray expression values than the Affymetrix algorithm MAS5.

In order to compare real data with a null dataset, we analyzed the behaviour of the three preprocessing techniques on permutated arrays (see Material and Methods). Since permutated arrays are entirely shuffled and uninformative, we expect them to be, on average, not correlated at all. However as previously reported by Lim and colleagues [26], this is not the case for the non-Affymetrix techniques we used (Figure 1B). RMA and GCRMA show a high mean inter-array correlation, which is decreasing with the sample size and this correlation is also much higher for odd sample sizes, and reminiscent of the oscillating behaviour in real arrays (Figure 1A). Average values for Figure 1 are shown in Table 1. In order to assess if these artifacts were due to the choice of correlation coefficient we repeated our analysis using Pearson's and Lin's correlation, but obtained nearly identical results (Additional File 1, Supplemental Figure S2 and Additional File 2 Figure S3).

**Table 1:** average values for inter-array correlation coefficients

*Sample size*	Original arrays			Permutated arrays		
		
	MAS5	RMA	GCRMA	MAS5	RMA	GCRMA
*2*	0.7704	0.8113	0.7825	3.31^-4^	0.0456	0.1795
	
*3*	0.7721	0.8547	0.8524	-9.88^-5^	0.7761	0.9144
	
*4*	0.7601	0.8220	0.8039	-4.63^-4^	0.1154	0.2647
	
*5*	0.7604	0.8460	0.8432	3.01^-5^	0.4316	0.7140
	
*6*	0.7557	0.8144	0.7949	-5.90^-5^	0.1086	0.2544
	
*7*	0.7584	0.8357	0.8212	-1.09^-5^	0.2481	0.5436
	
*8*	0.7555	0.8210	0.7981	1.00^-4^	0.0913	0.2274
	
*9*	0.7529	0.8334	0.8125	3.46^-5^	0.1587	0.4140
	
*10*	0.7536	0.8163	0.8048	1.20^-4^	0.0757	0.1929
	
*11*	0.7536	0.8242	0.8097	-1.01^-4^	0.1118	0.3170
	
*12*	0.7552	0.8156	0.8000	9.03^-5^	0.0637	0.1665
	
*13*	0.7523	0.8264	0.8061	1.85^-5^	0.0843	0.2546
	
*14*	0.7531	0.8154	0.8014	6.90^-5^	0.0547	0.1482
	
*15*	0.7537	0.8217	0.8087	2.82^-5^	0.0659	0.2048
	
*16*	0.7511	0.8146	0.8052	7.68^-5^	0.0467	0.1307
	
*17*	0.7511	0.8232	0.8062	-6.80^-5^	0.0540	0.1654
	
*18*	0.7532	0.8159	0.8022	-7.89^-5^	0.0411	0.1150
	
*19*	0.7546	0.8247	0.8059	9.65^-6^	0.0460	0.1418
	
*20*	0.7592	0.8228	0.8024	4.69^-6^	0.0364	0.1008
	
*30*	0.7602	0.8208	0.8022	3.09^-5^	0.0226	0.0620
	
*50*	0.7578	0.8233	0.8038	7.72^-6^	0.0124	0.0326
	
*100*	0.7543	0.8190	0.8023	-2.41^-8^	0.0058	0.0157

average inter-array correlation coefficients at different sample sizes, using three different normalization procedures.

MAS5 alone shows the expected no-correlation behaviour. It must be noted that, unlike the other two techniques, MAS5 uses a single-array summarization technique (a robust Tukey-biweight average of the probe values) which treats each sample separately.

We will focus on the cause of this behaviour observed when using RMA and GCRMA, trying to understand the mathematical and biological scenarios that could introduce such a massive artificial inter-array correlation for these two methods.

### Causes of RMA and GCRMA artifact generation

**Figure 2:**
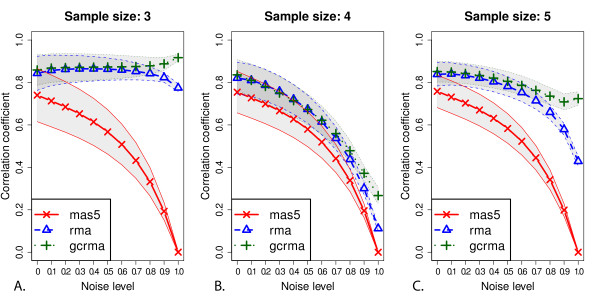
**MAS5, RMA and GCRMA behaviour on inter-array correlation for samples of 3,4 and 5 arrays upon incremental noise addition by adding values from real arrays to values from permutated arrays before normalization**. The "noise level" gives the fraction of the values that came from the permutated arrays (For more details see Materials and methods). The figure shows means and standard deviations, as in figure one using three different sample sizes, an even number, 4 (Figure 2B), and two odd numbers, 3 (Figure 2A) and 5 (Figure 2C).

We have already seen that the introduction of artificial similarities between arrays by RMA and GCRMA is particularly strong for small and odd sample sizes. In Figure 2 we show how adding an increasing amount of noise to microarray samples in the Arabidopsis dataset (see Material and methods) results in the expected loss-of-correlation behaviour for MAS5, GCRMA and RMA for an even sample size (Figure 2B). However, for sample size 3 (Figure 2A), RMA and GCRMA actually *add *inter-array correlation as noise is combined with the biological signal. The situation is still atypical for the next odd sample size (5 samples, Figure 2C).

Returning to our original Arabidopsis dataset, we observed that many probesets seem to yield completely identical values across different samples when processed by RMA or GCRMA. Datasets of three arrays normalized by RMA and GCRMA show, respectively, around 20% and 12% of the probesets population with identical values across all samples. The effect will decrease with increasing sample size (see Figure 1B) as previously reported in [15]. We therefore measured the tendency to yield identical expression estimates for any particular probeset after RMA normalization (ID tendency, see Materials and Methods) and compared it to several probeset characteristics.

**Figure 3:**
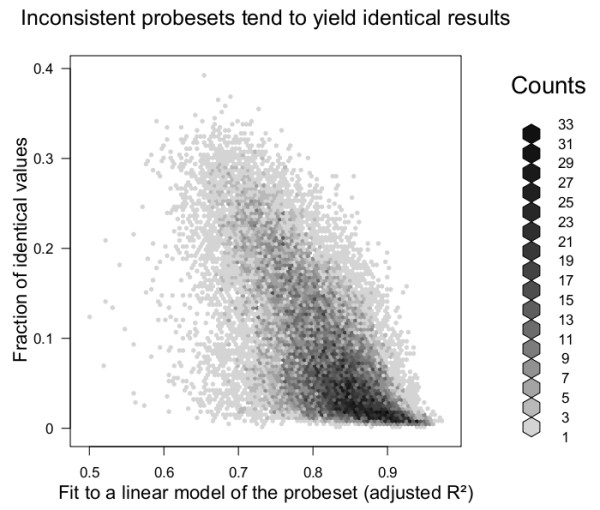
**Inverse correlation between probeset tendency to yield identical expression values and internal probeset consistency, measured as a probe linear model R^2^**. The x axis shows a fit to a linear model of any given probeset across 3707 Arabidopsis microarrays, using probeset sample means as explanatory variable. On the y axis the fraction of 3 sample subsets yielding 3 identical arrays for a given probeset is shown (10000 randomly picked groups were selected).

The ID tendency is inversely correlated (Spearman correlation coefficient = -0.624) to the probeset internal consistency (Figure 3), which we measured using the fit of the probeset to a linear model that measures concordance between probes.

This phenomenon is also particularly evident for lowly expressed probesets (Additional file 3, Figure S6) and those hybridizing to multiple targets (Additional file 4, Figure S7), especially if the different targets fall into different biological classes (Additional file 5, Figure S8).

In summary, RMA and GCRMA tend to yield identical values for probesets containing probes that yield grossly different measurements across samples, and therefore are either noise-driven or have multiple independent targets.

As the problem of bad probesets has been discussed before and been tackled by providing updated probeset definitions in the customCDF project [27], we assessed whether the oscillating behaviour for real data was still observable when using such an updated definition. However, qualitatively identical results were still obtained using such an updated probeset annotation (Additional file 6, Figure S1). This might be explained by the fact that expression is also inversely correlated to a probesets' tendency to give identical values across arrays.

Taken together, these results tell us that RMA (and GCRMA) introduce artificial correlation across microarrays driven by lowly expressed, internally inconsistent, multi-target and/or multi-function probesets.

### Median polish inconsistency

**Figure 4:**
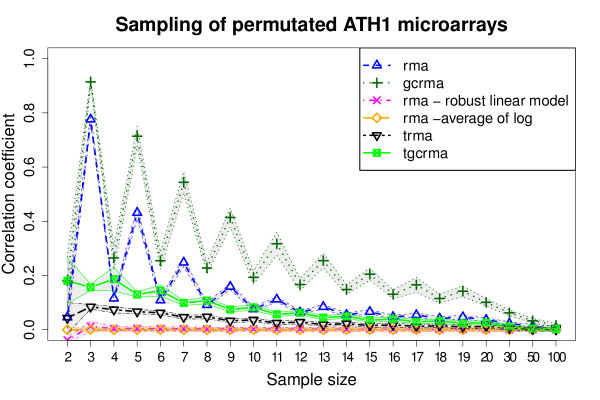
**Comparison of alternative RMA summarization steps on permutated datasets**. The original median polish summarizaion step is plotted with BioConductor alternatives and the transposed median polish of the tRMA method. 1000 groups of arrays for each sample size were selected, and then the averages and standard deviations of inter-array Spearman correlation coefficients were calculated and plotted as in Figure 1.

RMA [9] and the closely related method, GCRMA [11], differ only in the initial background correction step. Since the same effect is present in both methods (Figure 1B), we reasoned that the artifact generation should depend on either the shared normalization step (which is quantile normalization in both cases [28]), or on the probe summarization step (median polish [29]). We concluded that the effect cannot arise from quantile normalization, since substituting it by scale normalization or removing it completely yields qualitatively identical plots whereas the inclusion of a median polish step always introduced the effect, regardless of background correction and normalization procedures (Additional file 7, Figure S4). The shared artifact can therefore only be generated within the median polish summarization step. Indeed, substituting the median polish step with any other alternative available in the BioConductor RMA implementation eliminates the artificial inter-array correlation effect (Figure 4 and Additional file 7, Figure S4). As an example of multi-array summarization, we substituted the RMA default summarization with the robust least squares linear model summarization, described by [30], and show that this procedure almost completely removes inter-array correlation (magenta dashed line in Figure 4). On the other hand, as an example of single-array summarization, we used RMA with an "average of log" summarization, which simply computes the average of the logarithms of probe intensities for every probeset. This single-array summarization yields a predictable 0 correlation among all arrays (orange dashed line in Figure 4).

To identify why this artifact arises during the median polish procedure, we investigated the algorithm further. RMA and GCRMA apply median polish by creating a matrix from the measured values within each probeset, placing probes along each row, and samples along each column. The medians are subtracted from the intensities to cumulate residuals in each step and the grand effect (or median of medians) is subtracted from medians to cumulate probe effects in each step (Additional file 8, Figure S10).

This algorithm is more likely to introduce identical values with odd and small sample sizes, like the one depicted in Additional file 8, Figure S10. In such a case, the row medians will fall on a specific value and be transformed to zero during the first row sweep (Additional file 8, Figure S10B, top panel), this will increase the chance to have a zero as column median during column sweep (Additional file 8, Figure S10C, top panel).

Overall, the RMA implementation of the median polish algorithm shrinks all values in the probeset matrix to similar or identical values, with a stronger effect for samples, since it starts subtracting probe (row) medians. In the example of Additional file 8, Figure S10, the final sample values will be calculated by adding the grand effect to each column effect, and will therefore be equal to 8 for all samples.

It could be argued that the median polish summarization step could be helpful in the context of Differential Gene Expression analysis, since it will flatten unclear probeset matrices and therefore highlight strong signals. However, the result of generating completely identical expression values across arrays is not always beneficial. Moreover, this effect can be dramatically reduced by swapping the order of row/column median subtraction within median polish, or equivalently, by transposing the matrix created for each probeset, placing samples on rows and probes on columns. This alteration will introduce a presumably harmless similarity between probes within a probeset (which are assumed to be measuring the same quantity, and which don't form part of the output) while massively reducing the artificial sample identity.

To confirm this, we re-implemented the median polish summarization by inverting the order of the two sweep steps (Additional file 8, Figure S10), in what we call "transposed RMA" or tRMA. As shown in Figure 4, the inversion of median subtraction steps alone reduces the median polish effect to a very small residual inter-array correlation. This can be explained by the fact that the likelihood for the sample effects to give a zero value in tRMA is very low during the first iteration, as it would require perfectly identical medians of raw probe values (Additional file 8, Figure S10). Effectively, tRMA transfers the artifact of inter-correlation between sample to an inter-correlation between probe (in a common probeset) effects, which might be more plausible biologically (as all probes in a probeset should measure the same target) and remains contained within the procedure and not yielded as output of the preprocessing method.

The inter-array artificial correlation effect introduced by the median polish step is increased in GCRMA (Figure 4, dark green dotted line). As previously discussed by [26], GCRMA contains a potential problem in its background correction step, that adjusts probe intensity values through gene-specific binding. This introduces artificial inter-array correlations between probes with similar binding affinity, and therefore strengthens the effect of the following summarization step. However, substituting the median polish step with our transposed alternative "tGCRMA" (Figure 4, dark green line), massively reduces the inter-array correlation between permutated samples.

### Comparison between RMA and tRMA in biological contexts

The AffyComp open challenge benchmark [6,17] is a well known tool to evaluate summaries of Affymetrix probe level data, based on known concentration of transcripts in the so-called "spike-in" experiments by Affymetrix [4]. In order to demonstrate that our tRMA procedure still performs nearly as well as the original RMA implementation, we used the latest implementation of the AffyComp benchmark ("AffycompII") to compare the performance of the original RMA with our tRMA implementation. In Table 2 we show some of the most relevant scores, as calculated for the HGU95 Affymetrix spike-in series.

**Table 2:** comparison between RMA and tRMA in the affycomp benchmark

	MAS5	RMA	tRMA	Best possible
Signal detect slope	0.71	0.63	0.63	1

Signal detect R2	0.86	0.80	0.80	1

Obs-intended-fc slope	0.69	0.61	0.61	1

Obs-(low)int-fc slope	0.65	0.36	0.36	1

null log-fc IQR	0.85	0.19	0.20	0

null log-fc 99.9%	4.48	0.57	0.58	0

low AUC	0.07	0.40	0.39	1

med AUC	0.00	0.87	0.86	1

high AUC	0.00	0.46	0.44	1

weighted avg AUC	0.05	0.52	0.51	1

**Median SD**	**0.63**	**0.11**	**0.12**	**0**

low.slope	0.72	0.35	0.35	1

med.slope	0.80	0.76	0.76	1

high.slope	0.45	0.47	0.47	1

affycompII most indicative results (as in [6]) for MAS5, RMA and tRMA, spike-in HGU95 dataset. Differences between RMA and tRMA are trivial, especially when compared to other methods (see also [6]).

The differences between RMA and tRMA are minor when compared to the results for an independent method (MAS5): tRMA shares most of the qualities of RMA, without introducing inter-array correlations. It is interesting to highlight the fact that tRMA yields a higher median standard deviation (Median SD, in bold in Table 2) between spike-in replicates. This effect can be wrongly interpreted as tRMA's lower sensitivity; however, we now know that the original RMA median polish implementation is introducing identical values across experiments, and therefore artificially reducing the variance between spike-in replicates as well.

Since median polish alters inter-array correlation, sample classification is a common analysis that could be affected by this summarization step. Thus, we analyzed the AtGenExpress stress dataset for Arabidopsis [31], and calculated the capability of both preprocessing techniques to separate roots and shoots samples (see Material and Methods).

**Figure 5:**
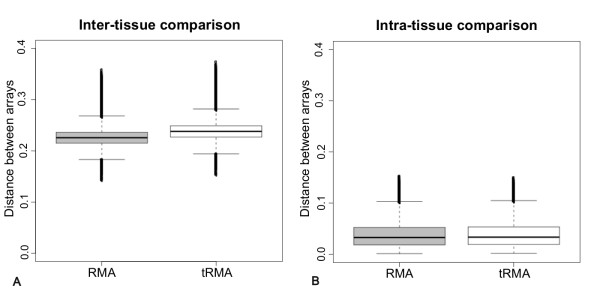
**Distances between Arabidopsis microarrays belonging to (A) different tissues (roots and shoots) and (B) the same tissue in 10000 5-samples subsets, calculated after RMA (left) preprocessing or tRMA (right) preprocessing**. Distances are reported on the y axis and calculated as (1-Spearman correlation coefficient).

As can be seen in Figure 5A, tRMA outperforms RMA as it increases the distance between different tissue samples (Wilcoxon test: p-value <2.2*10^-16^), while keeping similar low distances between samples coming from the same tissue (Figure 5B, Wilcoxon test: p-value = 0.935). As variance filtering is a common procedure for microarray clustering, we used only the 50% most varying genes in every subset and obtained similar results (Additional file 9, Figure S9A, inter-tissue distance, p-value<2.2*10^-16 ^and Additional file 9, Figure S9B, intra-tissue distance, p-value = 0.141). It can be concluded that tRMA increases the capability to discern different array conditions, when only a small number of microarrays have been used.

In order to compare the relative performance of RMA and tRMA when filtering on differentially expressed genes, we used a dataset that was previously used by [32], to tune classification where the origin of the RNA in each sample was known. Choosing a sample size of 5 where 2 pairs of 2 samples each came from the same specimen and one sample came from a different specimen, tRMA yields better classification results for almost all FDR corrected p-value thresholds (Additional file 10, Figure S5).

However, when filtering out lowly expressed genes [33], RMA performed generally as well as tRMA when performing sample classification on this dataset (data not shown). Unlike for the tissue sample dataset, RMA performed better than tRMA when filtering based on variance in this use case even when adding random noise to all samples (Additional file 11, Figure S11A). Only when noise was selectively added to non-paired samples did tRMA outperform RMA (Additional file 11, Figure S11B).

Furthermore, RMA performs slightly better than tRMA in 7 out of 14 of the Affycomp tests shown. Although objectively minor, these differences point out that tRMA may not necessarily be an improvement over RMA in all types of analyses.

## Discussion

The use of GCRMA and RMA preprocessing algorithms for Affymetrix GeneChip technology has received a remarkably broad adoption in the community due to their low computation time and to their superiority with respect to other methods in previous benchmarks.

However, one of the most relevant advantage of RMA and GCRMA in the AffycompII challenge [17], the low variance across replicates, seems to be partially the result of artificial inter-array correlation. Extending what was already noted by [26], we show that the artificially high similarity between samples given by RMA and GCRMA is caused by the shared median polish summarization step. This artificial behaviour is particularly strong in internally inconsistent, noise-driven and multi-hit probesets, and as a consequence identical results across arrays are generated. We analyzed this artifact effect for the Arabidopsis thaliana ATH1 Affymetrix GeneChip, but we found highly similar results in exploratory experiments on other organisms and platforms (specifically, human HG133 and E. coli Asv2 - data not shown).

The median polish step doesn't seem to pose a particular problem in differential gene expression analyses, because, on the contrary, it could enhance the differences of changing transcripts by shrinking most unclear probesets to identical values across experiments. In any case, the small underlying change in gene expression of such an unclear probeset would generally be below the cut-off value to be considered an 'interesting' gene. It is interesting to note, however, that median polish has already been shown to work poorly when compared to MAS5 summarization in correlation between E. coli operon members [25].

However, this artificial correlation can't be ignored in contexts where unbiased measurements are needed, like transcript clustering [20], genetic network reverse-engineering [21], sample classification [22,34] or global transcript models [23], and we show in the present paper how RMA can artificially decrease the gap between samples coming from different tissue types. Furthermore, we could show that filtering of differentially expressed genes leads to a worse sample classification performance for small odd sample sizes samples for RMA, when compared to tRMA. Nevertheless, depending on the use case, RMA performed better when filtering based on variance. Thus, our results raise issues, especially when small sample sizes are used, on the validity of many studies obtained on the basis of correlation measures after these normalization procedures were applied.

## Conclusions

We propose a minor change to the median polish algorithm that will almost eliminate the correlation artifacts without significantly affecting any of the positive RMA/GCRMA qualities. We provide a modified version of RMA named tRMA as a standalone R package (additional files 12 and 13). The method contains a modified version of the BioConductor [35] preprocessCore and affy packages that alter the median polish summarization step as described in Additional file 8, Figure S10 (bottom panel). Our tRMA method will offer the possibility to use an unbiased normalization technique both for differential gene expression analyses and for correlative studies based on microarray data.

## Methods

### Datasets

In order to obtain a vast, robust and condition-independent dataset, we downloaded all Arabidopsis thaliana ATH1 microarrays available from GEO [2] and removed truncated or unreadable files and genomic DNA experiments. This dataset comprised 3707 arrays and is henceforth referred to as the "*Arabidopsis dataset"*.

To test the abilities of RMA and tRMA to correctly cluster different tissue samples, we analyzed microarrays from the AtGenExpress stress study [31], contained in the Gene Expression Omnibus series GSE5620-GSE5628. This dataset (*root-shoot dataset*) comprises 248 samples, evenly distributed in shoot and root tissues.

To further assess sample classification performance of RMA and tRMA, we focused on a human breast cancer dataset published by [36] and reanalyzed by [32]. This dataset contains 98 surgical specimens, 18 of which belong to 9 replicate pairs in which two samples were taken from adjacent sections of the same frozen block.

### Permutation of CEL files

In order to compare real samples with completely uninformative samples, we decided to randomly permute the raw signal intensities of the Arabidopsis dataset as in [26]. In brief, every Perfect Match (PM) probe and its Mismatch (MM) counterpart were reassigned to a random probeset within the same microarray. This generates information-less probesets while keeping the properties of the original probe intensity distribution.

### Microarray preprocessing procedures

We compared the microarray preprocessing procedures RMA [9], GCRMA [11] and MAS5 [7] using the software implementations available from BioConductor[35]. In every case, the default parameters were used. All final outputs, including MAS5 ones, were analyzed on the log2 scale.

### Inter-array correlation analysis

The behaviour of the three microarray preprocessing procedures was analyzed in the context of randomly selected subsets of the Arabidopsis dataset. Different sample sizes were selected (2, 3, 4, 5, 6, 7, 8, 9, 10, 11, 12, 13, 14, 15, 16, 17, 18, 19, 20, 30, 50 and 100) according to the realistic scale of a single-experiment dataset. For each sample size, 1000 subsets were selected and normalized. For each normalized subset, we calculated inter-array Spearman correlations and then plotted the overall mean and standard deviation of these correlations for each sample size.

The same procedure was then repeated for the permutated Arabidopsis dataset.

### Noise robustness analysis

In order to assess the response of RMA, GCRMA and MAS5 to data perturbation, we generated increasingly noisy samples using the formula:

$$I=w_{o}*O+w_{p}*P$$

where ***I ***is the final probe intensity, ***O ***and ***P ***are, respectively, the original intensity and a permuted intensity. ***w_o _***and ***w_p _***are the weights given to both (where ***w_o_***+***w_p _***= 1). ***W_p _***is referred to as noise level.

### Linear model for measuring internal probeset consistency

Internal probeset consistency was analyzed by developing a linear model. Given a matrix for each probeset, where columns are samples and rows are probes, we ranked the values row-wise and determined the model

$${\text{p}}_{\text{ij}}=\text{w}*{\text{S}}_{\text{j}}+\text{i}+\varepsilon$$

The model tries to predict every i^th ^probe intensity rank in the j^th ^sample (**p_ij_**) using as explanatory variable the j^th ^sample effect, **S_j_, **calculated as the probe's mean rank for the sample. The model will then try to adjust the sample effect weight **w **and the intercept **i **to minimize the unexplained error ε.

It is apparent that the R^2 ^for this model will be high when all probes within a probeset behave consistently relative to each other across different experiments, i.e. when the probe rank in a specific experiment is predicted quite well by the probe's mean rank across experiments. On the other hand, a low R^2 ^will result from probes acting inconsistently across experiments, e.g. with some probes ranking particularly highly in some experiments yet poorly in others. The modelling procedure is provided as an R function (fitLM, Additional file 14) to determine internal consistency of probesets together with a function to reproduce the figures (Additional File 15).

### Transposed RMA

With the goal of reducing inter-array correlation artifacts without losing the positive features of RMA, we modified the RMA median polish source code of the preprocessCore library available on BioConductor [35]. Our method simply changes the order of median substitution, starting from column (sample-wise medians) instead of from rows (probe-wise medians), and was therefore called "transposed RMA" (or tRMA). tRMA code is available in the Additional files 12 and 13 and can be run in the R environment [27].

### Affycomp benchmark

In order to evaluate and benchmark our newly proposed preprocessing method, tRMA, we adopted the criteria developed for the AffycompII challenge [9,6] using the two Affymetrix spike-in datasets HGU95 and HGU133 and the Affycomp online tool [6].

### Sample classification performance

From the *root-shoot dataset*, we randomly selected 10000 groups of 5 arrays composed of 3 samples from one tissue type, and 2 from the other. Each dataset was normalized using tRMA or RMA and distances between arrays coming from the same tissue (intra-tissue distance) and between arrays coming from different tissues (inter-tissue distance) were determined. Distances were calculated as (1-Spearman correlation coefficient) using either all probe sets or only the 50% showing the highest variance.

Secondly, a dataset previously used by [32] to assess microarray performance was used to determine the percentage of correctly clustered subsets of 5 microarrays. From the dataset, two couples of samples coming from the same tumor or non tumor specimen, plus a different specimen were sampled. Probe-sets were selected based on differential expression between the samples using the limma package applying different p-value thresholds corrected using the Benjamini-Hochberg method [37]. These p-values are obtained by a specific combination of empirical Bayes methods and linear models described in [35]. The outcome of the normalization was defined as "correct" if, for every sample in a couple, its highest correlation coefficient against all other samples is the other correct member of the couple, which would lead to them being clustered together. The sampling was repeated 1000 times for each different p-value. The increase in the performance of tRMA when compared to RMA was assessed using a Fisher's exact test with Benjamini Hochberg correction.

The human dataset was used also to perform a test on clustering perfomance on groups of genes sorted by variance, as described by [32], but using only subsets of five samples (belonging to three groups). This test was performed for RMA and tRMA at different probe noise levels, added following the procedure described previously in the "Noise robustness analysis" paragraph. The noise was either added to all samples uniformly (Additional file 11, Figure S11A) or only to two unrelated samples (i.e. belonging to different replicate groups) (Additional file 11, Figure S11B).

## Abbreviations

RMA: Robust Multiarray Algorithm; tRMA: transposed Robust Multiarray Algorithm; MAS5: MicroArray Suite 5; PM: Perfect Match probe; MM: MisMatch probe

## Authors' contributions

FMG conceived of the study, carried out the experiments, performed the statistical analysis and drafted the manuscript. AB participated in the study design and provided technical assistance on the linear model experiments. ML and BU helped drafting the manuscript and in the study design. All authors read and approved the final manuscript.

## Supplementary Material

Additional file 1 **Figure S2**. drawn as in Figure 1 of the main paper, inter-array correlation for real (A) and permutated (B) Arabidopsis ATH1 microarrays, with different sample sizes, using Pearson correlation. Click here for file

Additional file 2 **Figure S3**. drawn as in Figure 1 of the main paper, inter-array correlation for real (A) and permutated (B) Arabidopsis ATH1 microarrays, with different sample sizes, using Lin correlation. Click here for file

Additional file 3 **Figure S6**. inverse correlation between probeset tendency to yield identical expression values and mean probeset expression. On the x axis the log2 of the mean probeset expression across 3707 Arabidopsis microarrays is shown. On the y axis the fraction of 3 samples subsets yielding 3 identical arrays for a given probeset is shown (10000 randomly picked groups were selected). Click here for file

Additional file 4 **Figure S7**. positive correlation between the number of distinct targets hybridized by a probeset and the tendency of a probeset to yield identical expression values across arrays. This tendency is calculated as the fraction of RMA normalized subsets of 3 arrays yielding 3 identical results for the given probeset. Within each boxplot the number of probesets in the category is indicated. Click here for file

Additional file 5 **Figure S8**. identical arrays output for multi-target probesets matching only one (left) or multiple (right) MapMan functional classes [38,39]. Click here for file

Additional file 6 **Figure S1**. drawn as in Figure 1 of the main paper, inter-array correlation for real (A) and permutated (B) Arabidopsis ATH1 microarrays, with different sample sizes, and updated probeset mapping provided by CustomCDF [27]. Click here for file

Additional file 7 **Figure S4**. drawn as in Figure 1 of the main paper, however standard deviation is plotted by using bars. Several combinations of background correction, normalization and summarization steps, on real arrays (left side) and permutated arrays (right side) are reproduced. Background methods: RMA.2 = RMA background correction, MAS = MAS5 background correction, GCRMA = GCRMA background correction, NA = no background correction Normalization methods: scaling normalization, quantile normalization, NA = no normalization, Summarization methods: median.polish = median polish (default method in RMA and GCRMA), tukey.biweight = Robust estimation based on Tukey's biweight function (default method in MAS5), average.log = average of log of probe intensities (single-array technique), median.log = median of log of probe intensities (single-array technique), rlm = robust linear model, tRMA = transposed median polish (method used by tRMA) Click here for file

Additional file 8 **Figure S10**. detail of the (t)RMA median polish procedures for a single probeset. Using alternating row and column sweeps, medians are calculated and subtracted until convergence is reached. In the case of RMA (upper panel), this can lead to the generation of many zeroes in a column, which subsequently could lead to the column effect also being zero, since the column effect is calculated as the median of the column residuals. In the case of tRMA (lower panel) zeroes introduced during a column sweep would lead to rather similar row (i.e. probe) effects. Click here for file

Additional file 9 **Figure S9**. distances between Arabidopsis microarrays belonging to (A) different tissues (roots and shoots) and (B) the same tissue in 1000 5-samples subsets, calculated after RMA (left) preprocessing or tRMA (right) preprocessing. Distances are reported on the y axis and calculated as (1-Spearman's correlation coefficient). For every subset, only the top 50% variance-wise probesets were used for calculating the distance. Click here for file

Additional file 10 **Figure S5**. percentage of correctly clustered subsets of 1000 samples of 5 microarrays using a clinical dataset from [32]. Different p-value thresholds to select genes used in the sample classification are shown. P-values were calculated using limma [38] and corrected using Benjamini-Hochberg method [37]. Significant differences (corrected p-value <0.05) in the proportions of the right classification determined by a Fisher's exact test are indicated by a blue line. Click here for file

Additional file 11 **Figure S11**. clustering performance for RMA (red solid line) and tRMA (black dashed line) over five-samples subsets of a human cancer dataset. Increasing number of genes, sorted by variance, are used in the calculation of clustering. Different noise levels are added to all samples (top panel A) or only to two unrelated samples (bottom panel B). Click here for file

Additional file 12 **trma source code for R (any Operating System), compatible with R.2.10.0 or later**. R package source code of the trma procedure, where the median polish summarization step implements a "column-first" median calculation Click here for file

Additional file 13 **tRMA package for R (Windows), compatible with R.2.10.0 or later**. R package for Windows of the trma procedure, where the median polish summarization step implements a "column-first" median calculation Click here for file

Additional file 14 **fitLM function and probesets examples**. a small R function to detect internal probesets' inconsistencies. The function, included in the tRMA package, here provides two probesets' examples. Click here for file

Additional file 15 **supplementary R code for plot generation**. a collection of R scripts used to generate the paper's pictures. Click here for file
