# Supplementary figures and images for: Algorithm-driven Artifacts in median polish summarization of Microarray data
Source: BMC Bioinformatics. 2010 Nov 11;11:553. doi: 10.1186/1471-2105-11-553 (PMC2998528; doi:10.1186/1471-2105-11-553)

# Inter-array correlation

original arrays

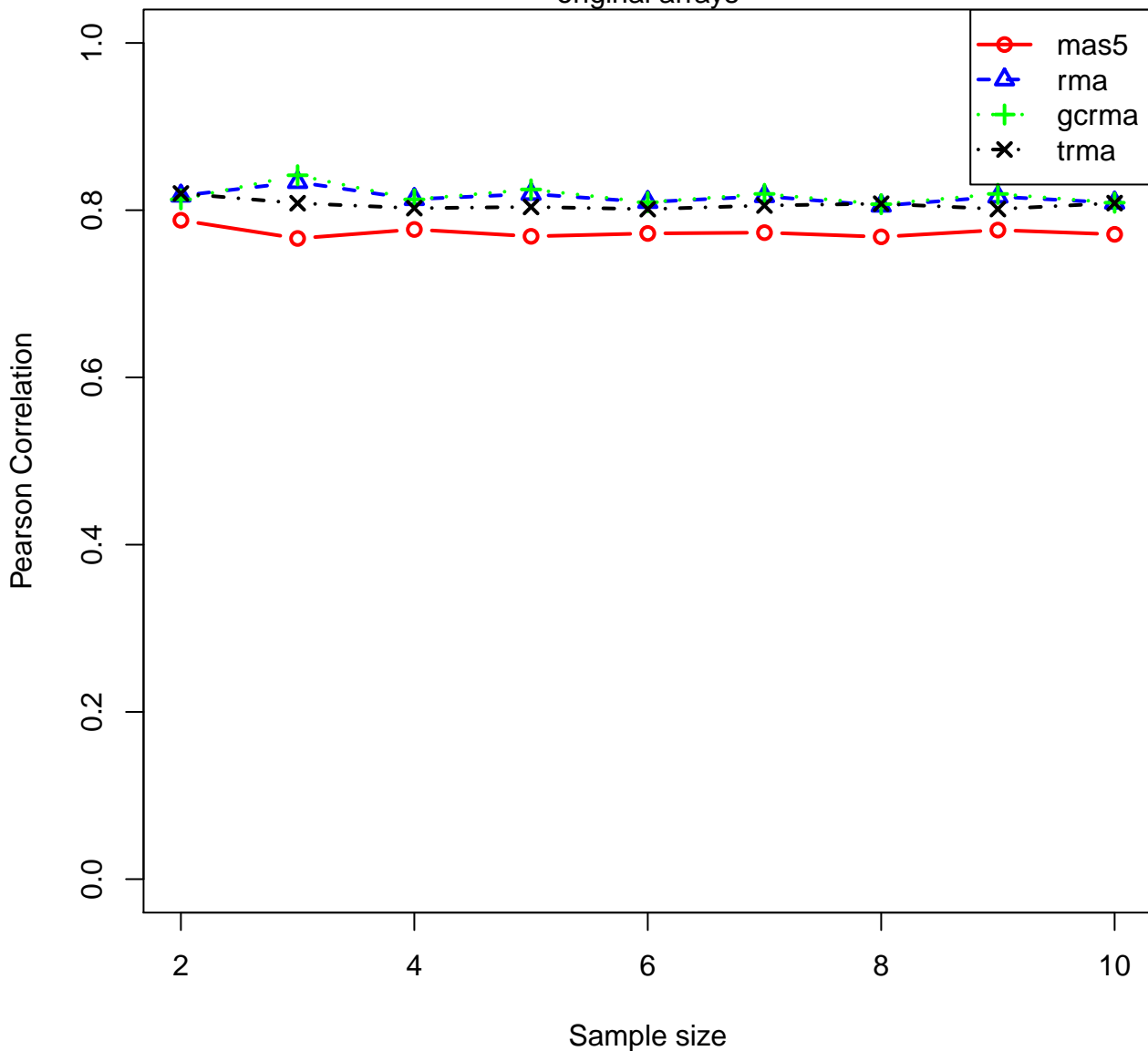

# Inter-array correlation

permuted arrays

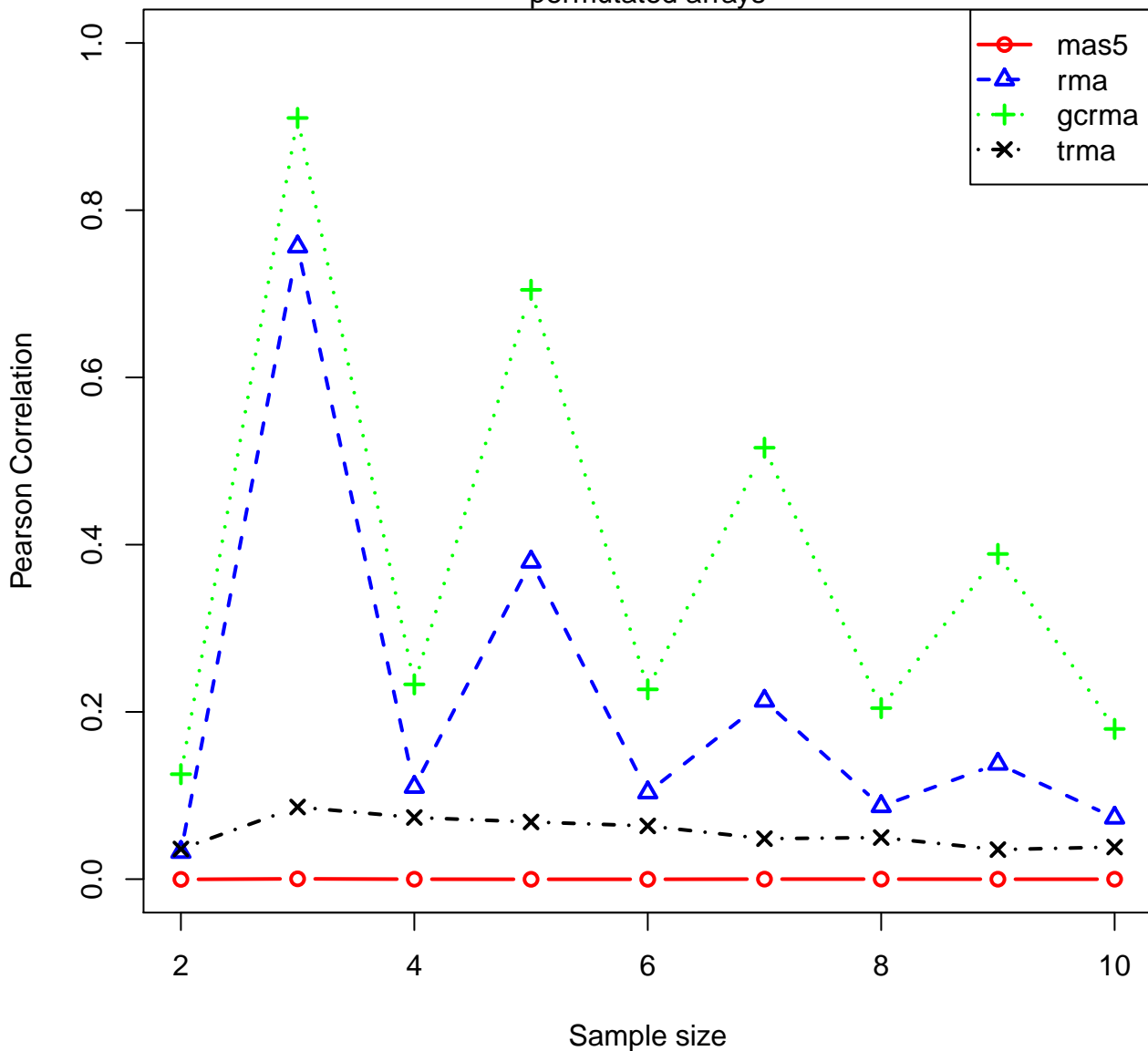

Supplement: Additional file 1 — Figure S2. drawn as in Figure 1 of the main paper, inter-array correlation for real (A) and permutated (B) Arabidopsis ATH1 microarrays, with different sample sizes, using Pearson correlation. [file 1471-2105-11-553-S1.PDF]

# Inter-array correlation

original arrays

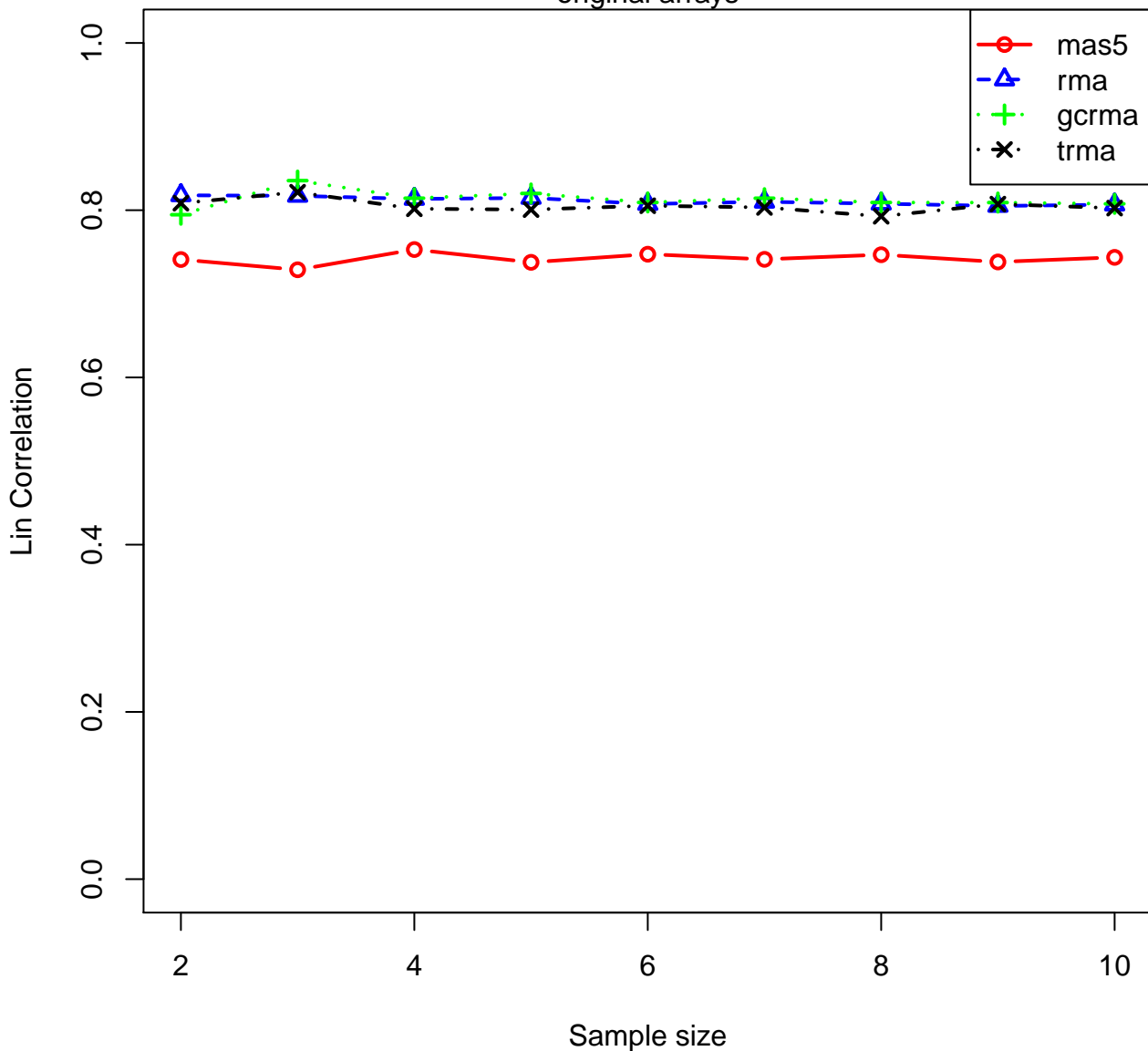

# Inter-array correlation

permuted arrays

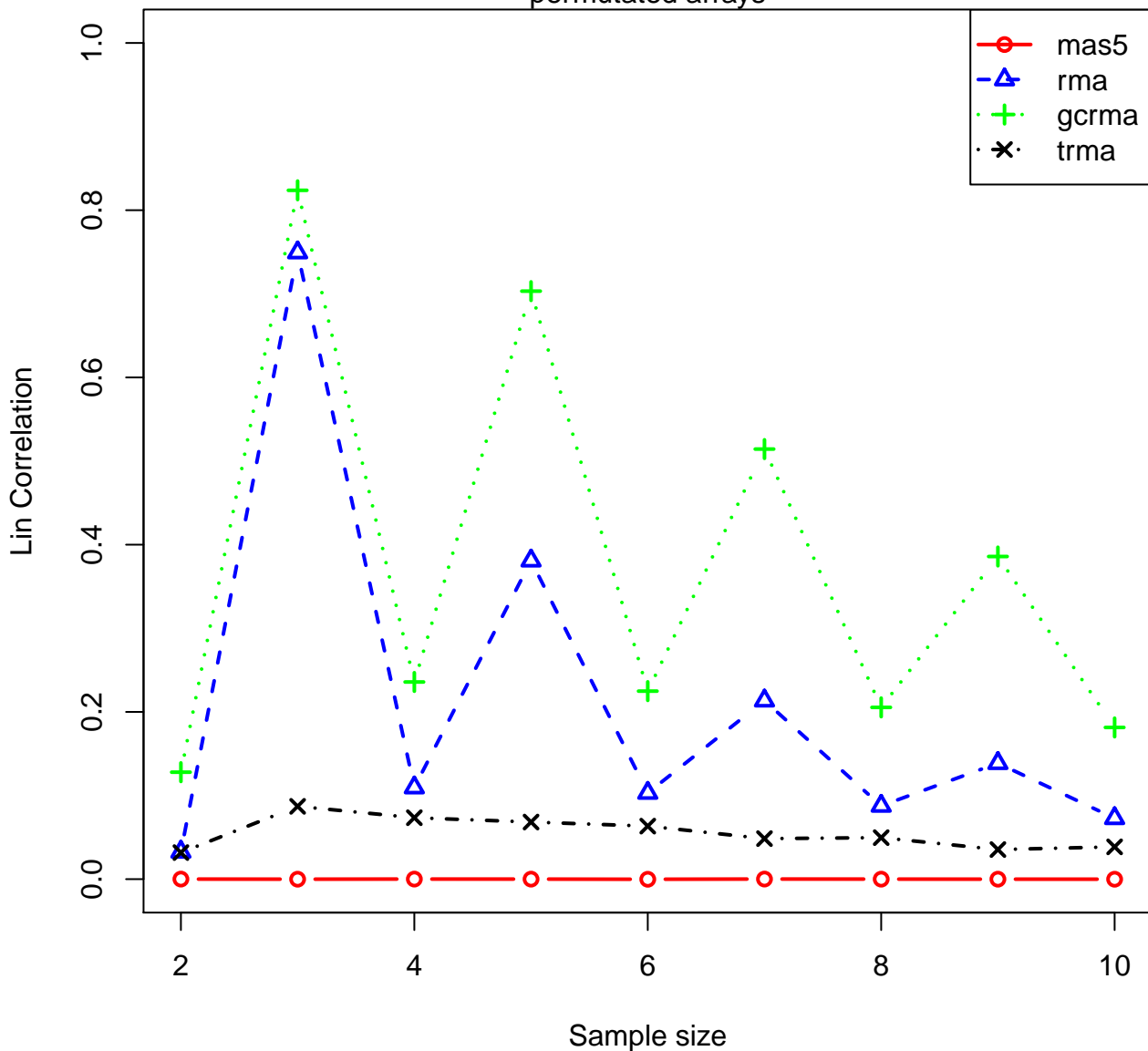

Supplement: Additional file 2 — Figure S3. drawn as in Figure 1 of the main paper, inter-array correlation for real (A) and permutated (B) Arabidopsis ATH1 microarrays, with different sample sizes, using Lin correlation. [file 1471-2105-11-553-S2.PDF]

# Inter-array correlation effect and probeset expression

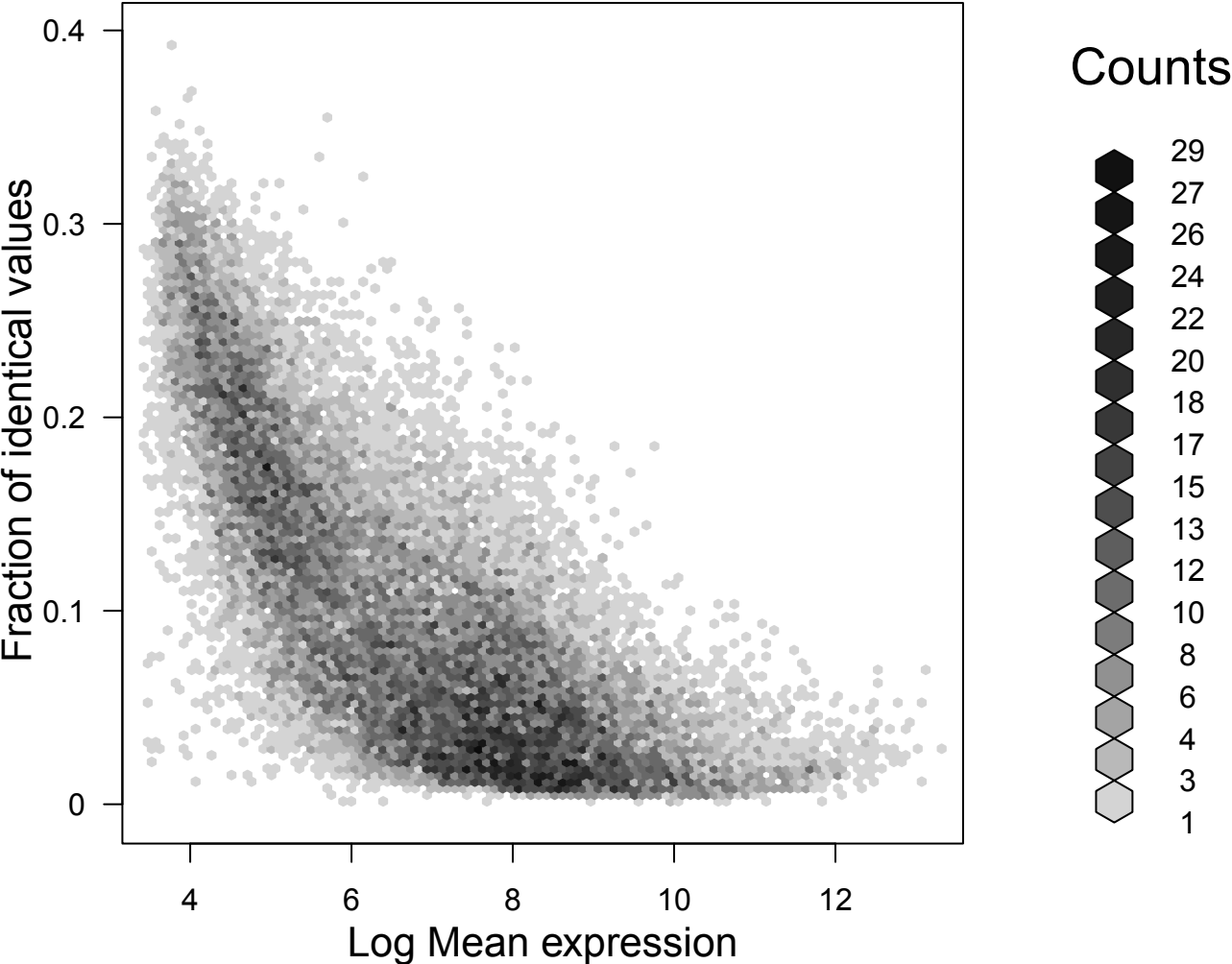

Supplement: Additional file 3 — Figure S6. inverse correlation between probeset tendency to yield identical expression values and mean probeset expression. On the x axis the log2 of the mean probeset expression across 3707 Arabidopsis microarrays is shown. On the y axis the fraction of 3 samples subsets yielding 3 identical arrays for a given probeset is shown (10000 randomly picked groups were selected). [file 1471-2105-11-553-S3.PDF]

Fraction of identical values

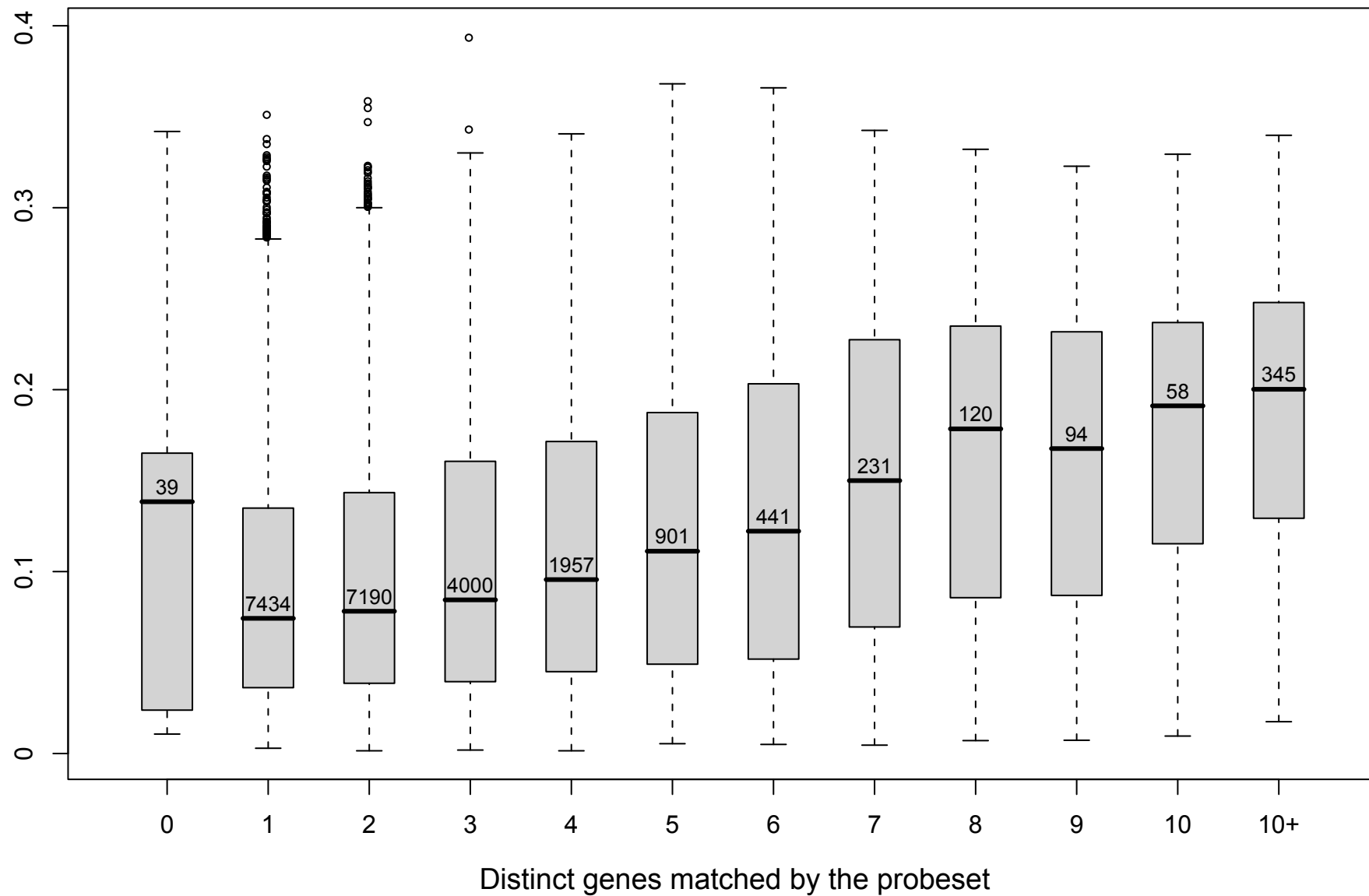

Supplement: Additional file 4 — Figure S7. positive correlation between the number of distinct targets hybridized by a probeset and the tendency of a probeset to yield identical expression values across arrays. This tendency is calculated as the fraction of RMA normalized subsets of 3 arrays yielding 3 identical results for the given probeset. Within each boxplot the number of probesets in the category is indicated. [file 1471-2105-11-553-S4.PDF]

# MapMan functional classes targeted by multi-gene probesets

Wilcoxon test p-value:  $<2.2e-16$

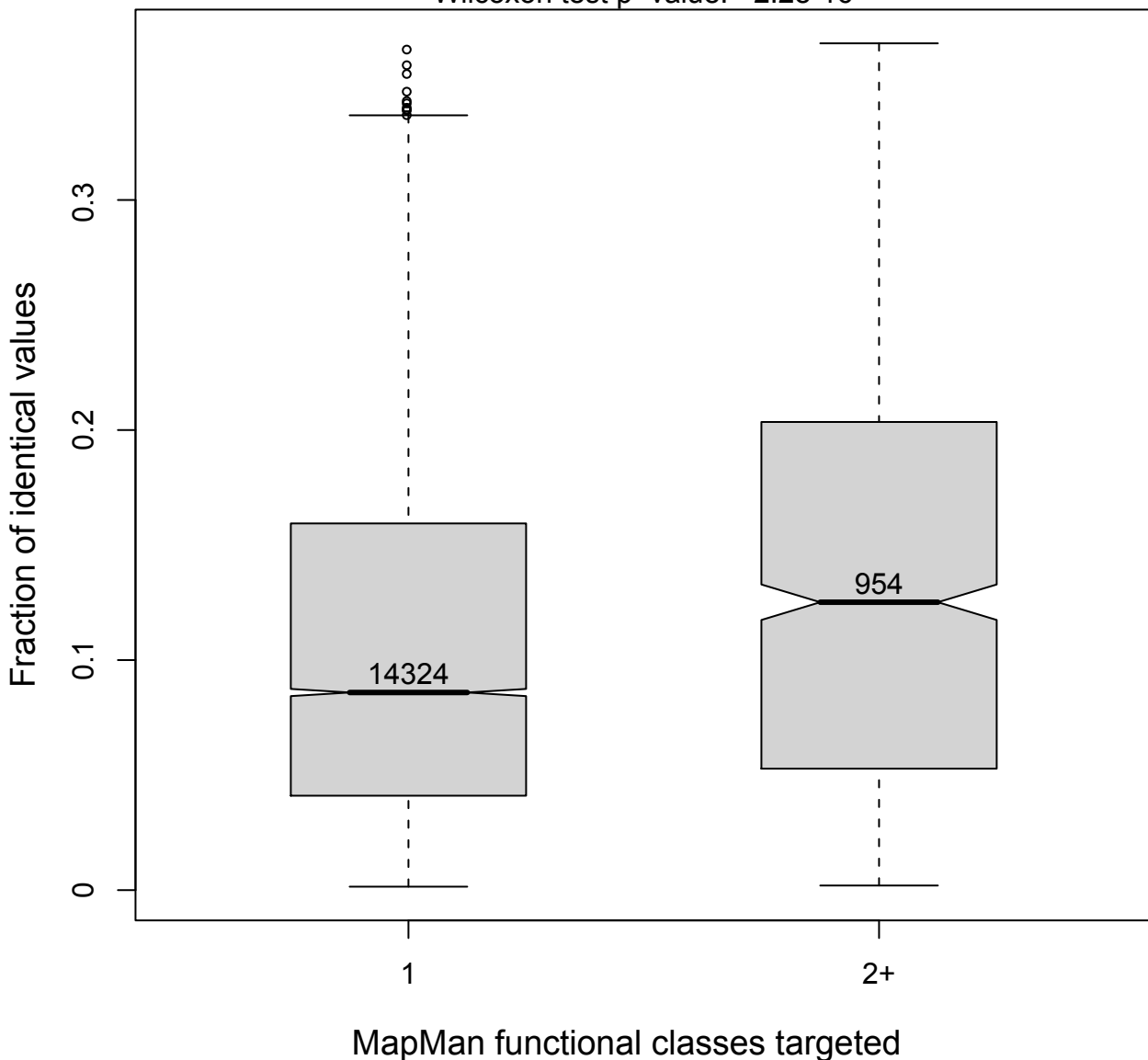

Supplement: Additional file 5 — Figure S8. identical arrays output for multi-target probesets matching only one (left) or multiple (right) MapMan functional classes [38,39]. [file 1471-2105-11-553-S5.PDF]

**Inter-tissue comparison**

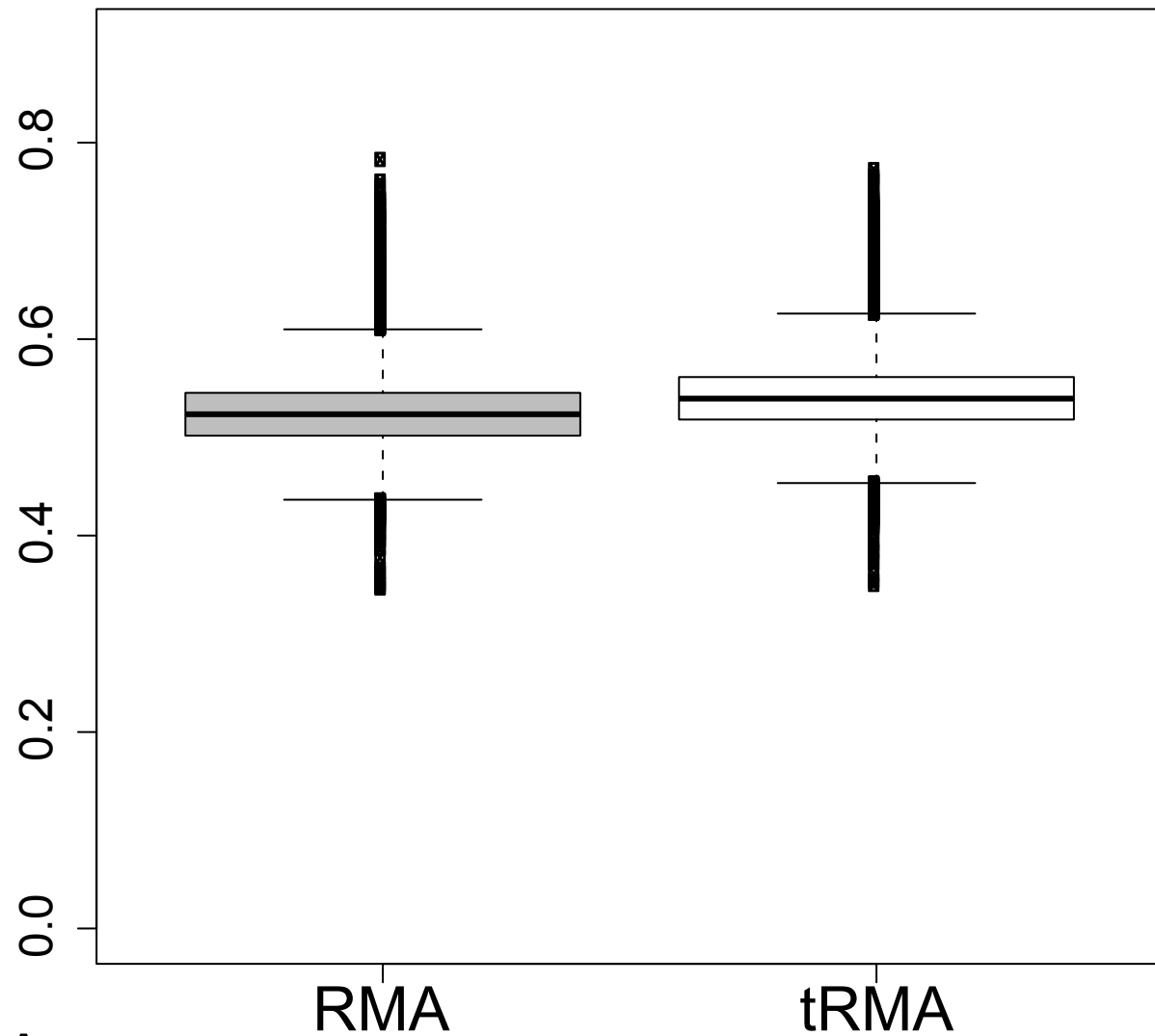

**A**

**Intra-tissue comparison**

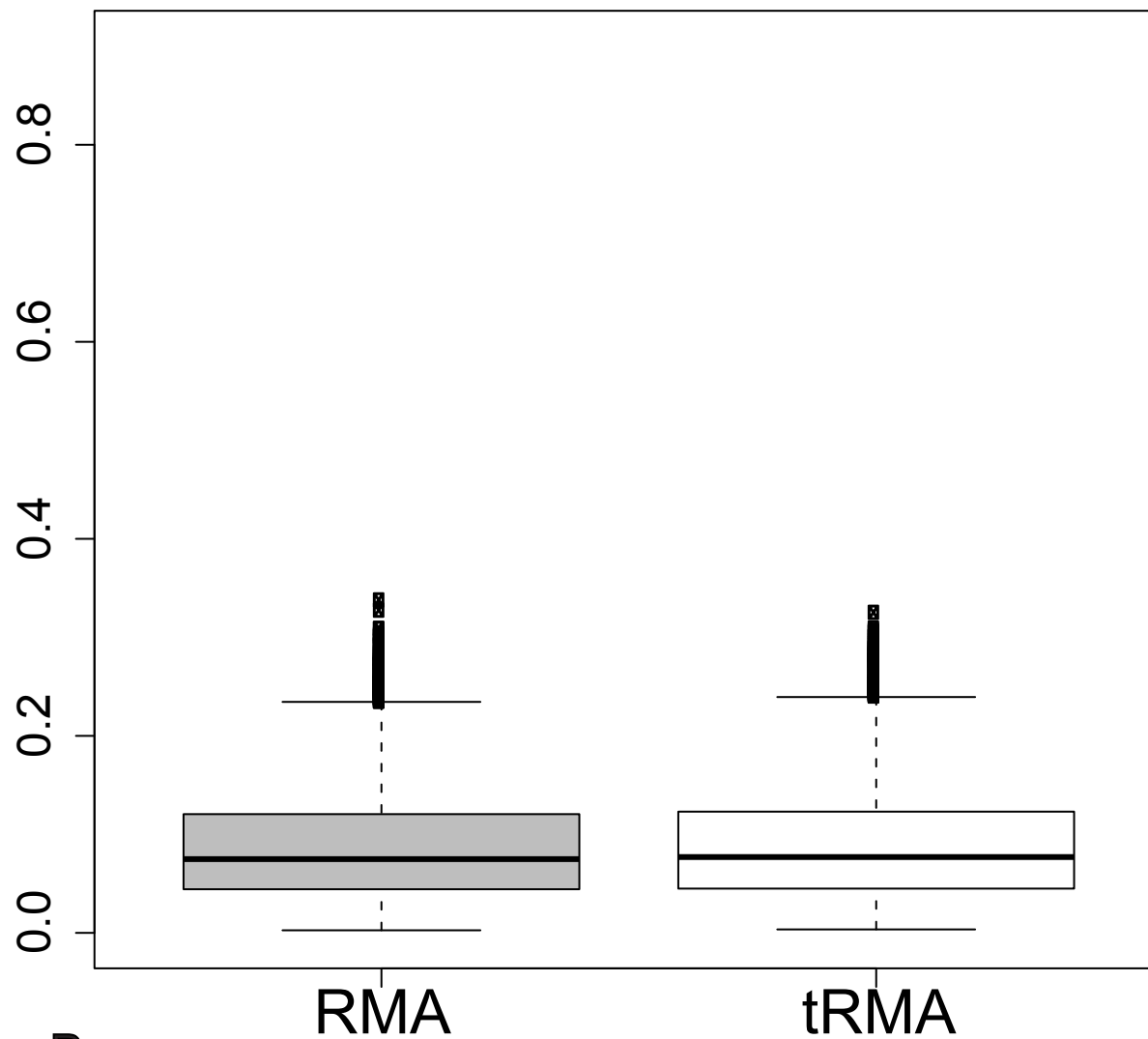

**B**

Supplement: Additional file 9 — Figure S9. distances between Arabidopsis microarrays belonging to (A) different tissues (roots and shoots) and (B) the same tissue in 1000 5-samples subsets, calculated after RMA (left) preprocessing or tRMA (right) preprocessing. Distances are reported on the y axis and calculated as (1-Spearman's correlation coefficient). For every subset, only the top 50% variance-wise probesets were used for calculating the distance. [file 1471-2105-11-553-S9.PDF]

# tRMA and RMA

1000 samplings

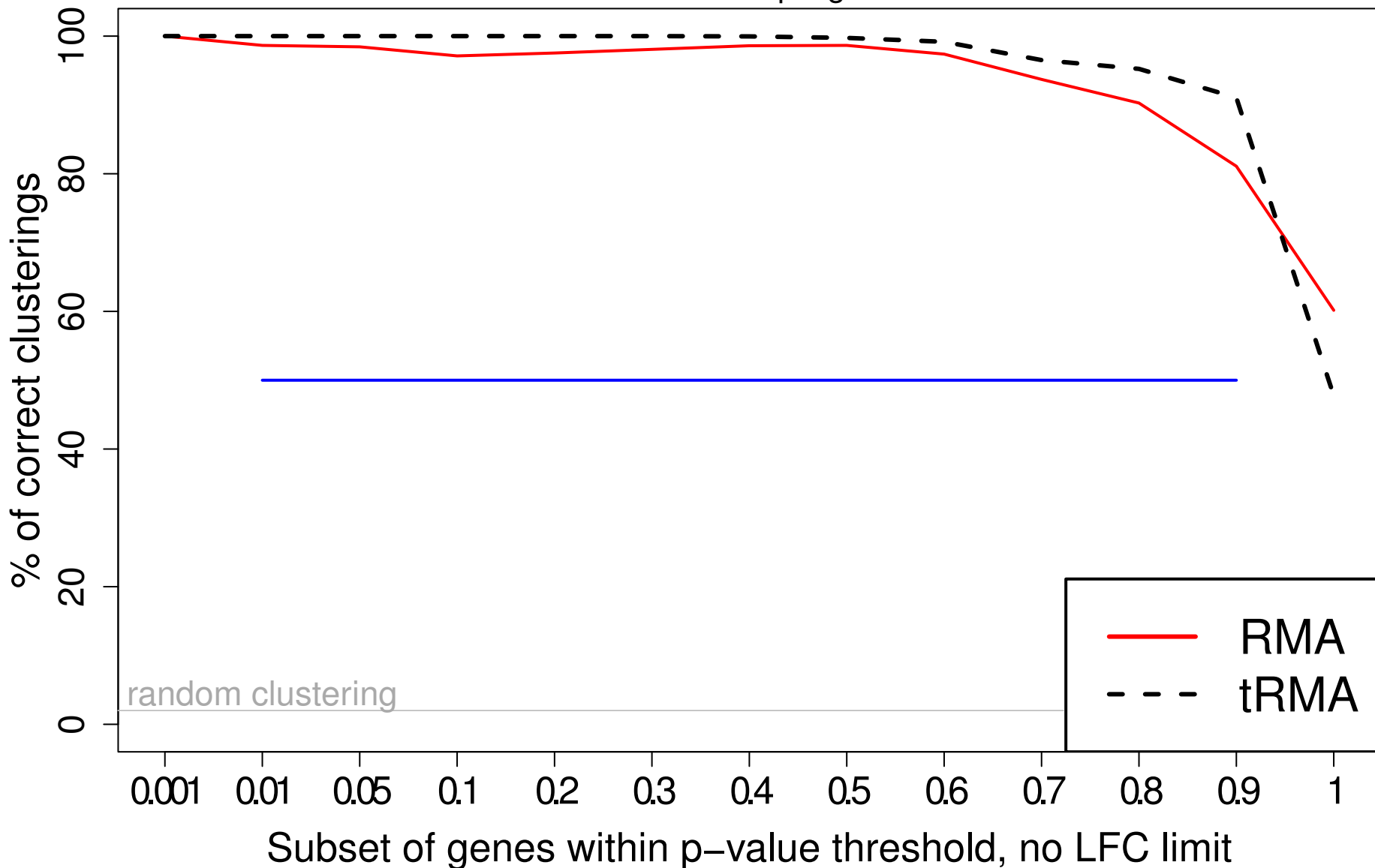

Supplement: Additional file 10 — Figure S5. percentage of correctly clustered subsets of 1000 samples of 5 microarrays using a clinical dataset from [32]. Different p-value thresholds to select genes used in the sample classification are shown. P-values were calculated using limma [38] and corrected using Benjamini-Hochberg method [37]. Significant differences (corrected p-value <0.05) in the proportions of the right classification determined by a Fisher's exact test are indicated by a blue line. [file 1471-2105-11-553-S10.PDF]

A.

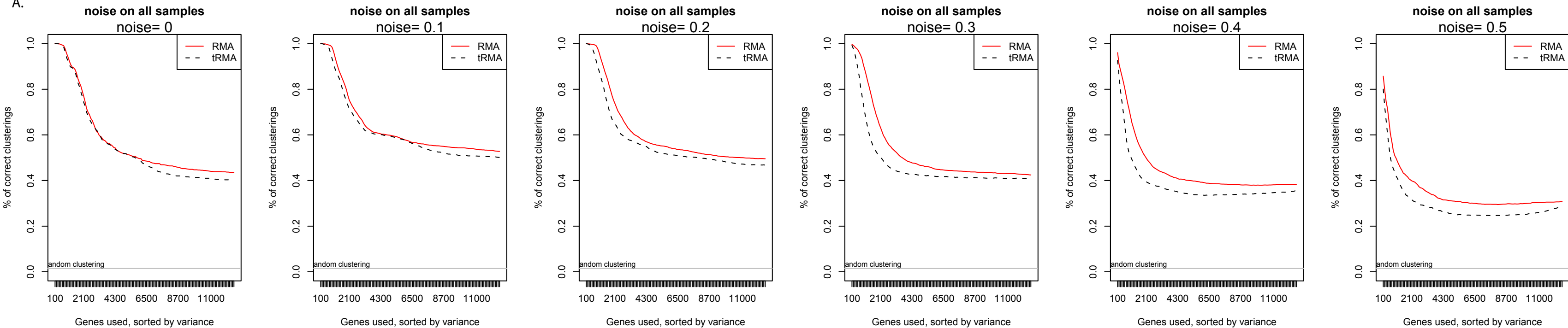

B.

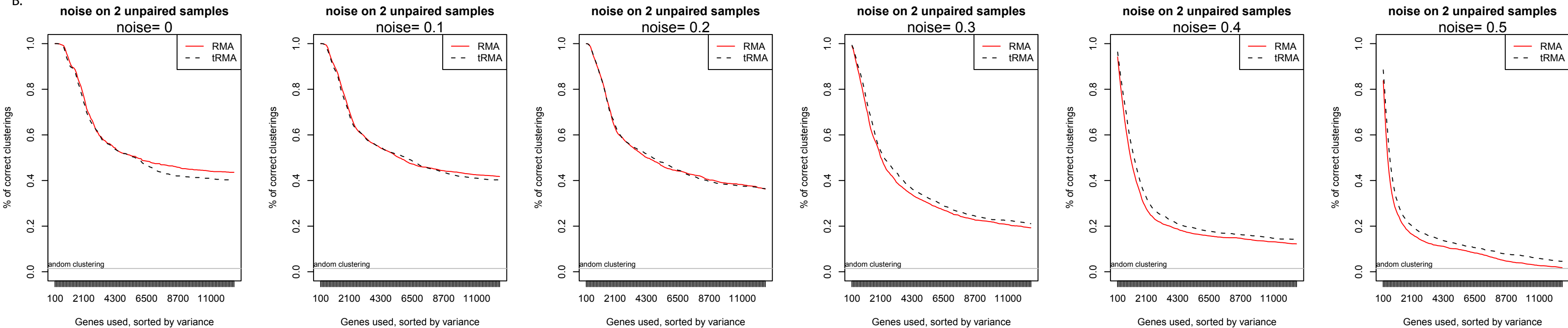

Supplement: Additional file 11 — Figure S11. clustering performance for RMA (red solid line) and tRMA (black dashed line) over five-samples subsets of a human cancer dataset. Increasing number of genes, sorted by variance, are used in the calculation of clustering. Different noise levels are added to all samples (top panel A) or only to two unrelated samples (bottom panel B). [file 1471-2105-11-553-S11.PDF]
