# Supplementary material for: Algorithm-driven Artifacts in median polish summarization of Microarray data
Source: BMC Bioinformatics. 2010 Nov 11;11:553. doi: 10.1186/1471-2105-11-553 (PMC2998528; doi:10.1186/1471-2105-11-553)
Supplement: Additional file 6 — Figure S1. drawn as in Figure 1 of the main paper, inter-array correlation for real (A) and permutated (B) Arabidopsis ATH1 microarrays, with different sample sizes, and updated probeset mapping provided by CustomCDF [27]. [file 1471-2105-11-553-S6.PDF]

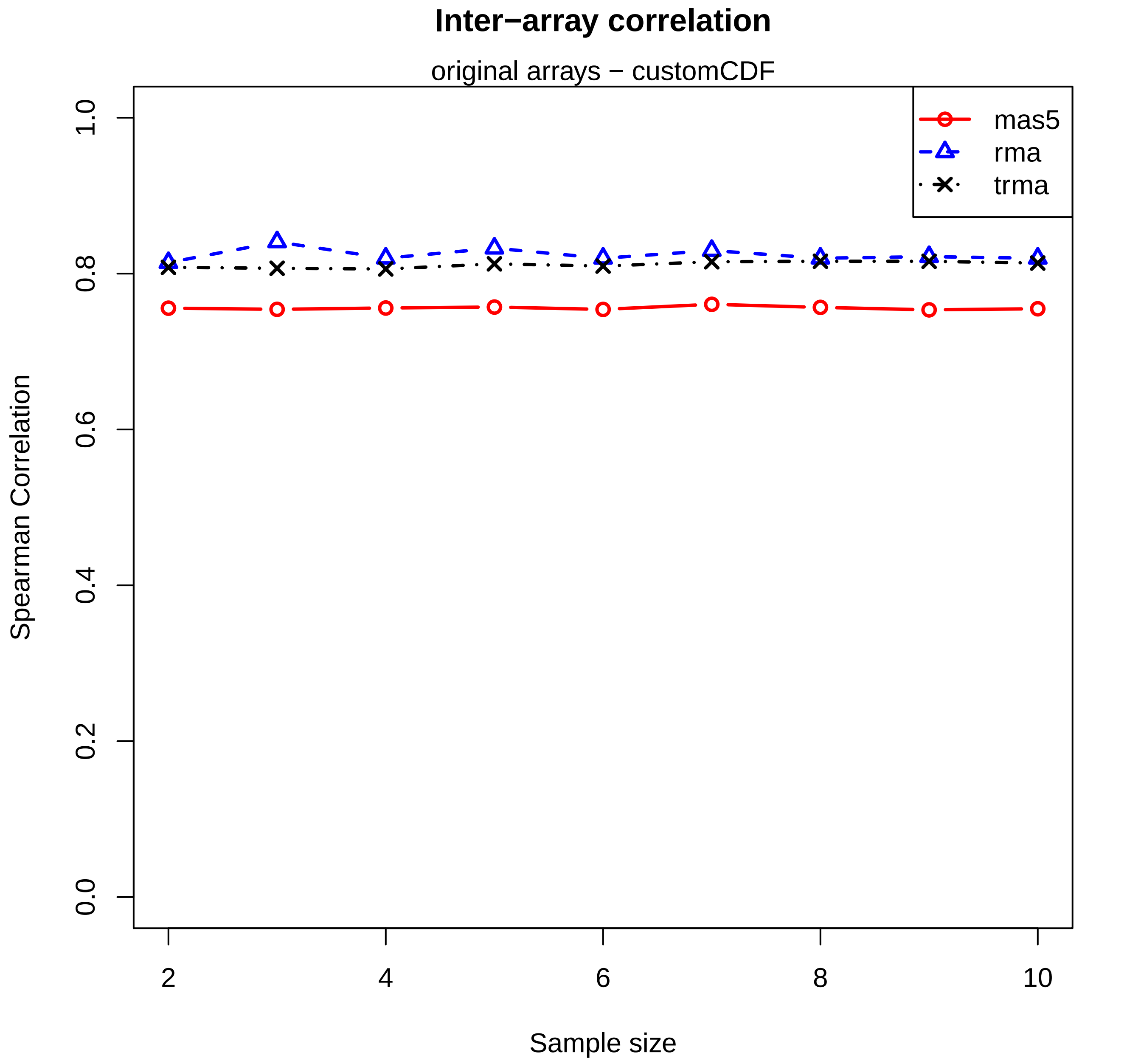

Inter−array correlation
original arrays − customCDF
mas5
rma
trma
Spearman Correlation
0.0
0.2
0.4
0.6
0.8
1.0
2
4
6
8
10
Sample size

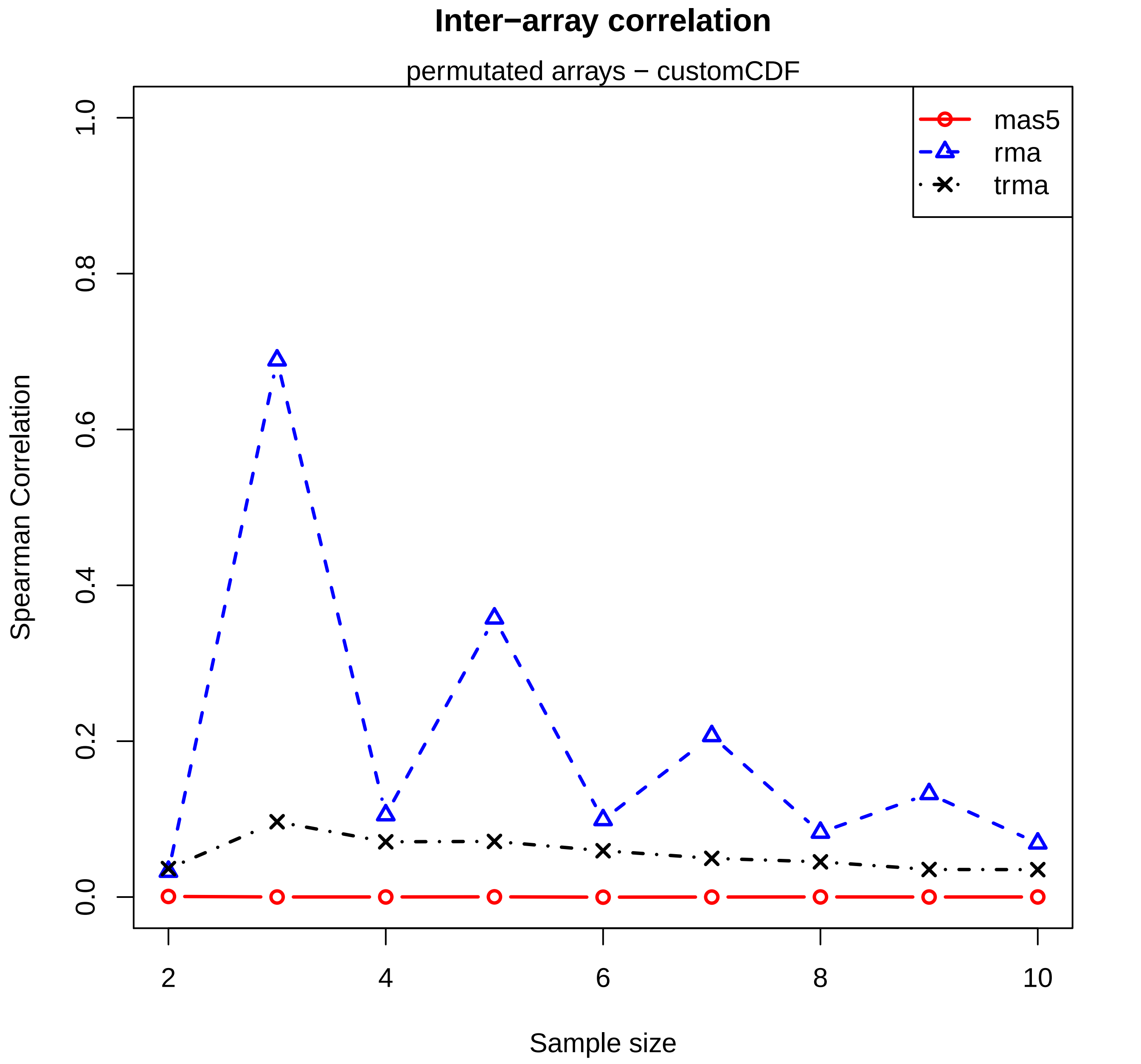
Inter−array correlation
permutated arrays − customCDF
mas5
rma
trma
Spearman Correlation
0.0
0.2
0.4
0.6
0.8
1.0
2
4
6
8
10
Sample size
