# Supplementary material for: Algorithm-driven Artifacts in median polish summarization of Microarray data
Source: BMC Bioinformatics. 2010 Nov 11;11:553. doi: 10.1186/1471-2105-11-553 (PMC2998528; doi:10.1186/1471-2105-11-553)
Supplement: Additional file 7 — Figure S4. drawn as in Figure 1 of the main paper, however standard deviation is plotted by using bars. Several combinations of background correction, normalization and summarization steps, on real arrays (left side) and permutated arrays (right side) are reproduced. Background methods: RMA.2 = RMA background correction, MAS = MAS5 background correction, GCRMA = GCRMA background correction, NA = no background correction Normalization methods: scaling normalization, quantile normalization, NA = no normalization, Summarization methods: median.polish = median polish (default method in RMA and GCRMA), tukey.biweight = Robust estimation based on Tukey's biweight function (default method in MAS5), average.log = average of log of probe intensities (single-array technique), median.log = median of log of probe intensities (single-array technique), rlm = robust linear model, tRMA = transposed median polish (method used by tRMA) [file 1471-2105-11-553-S7.PDF]

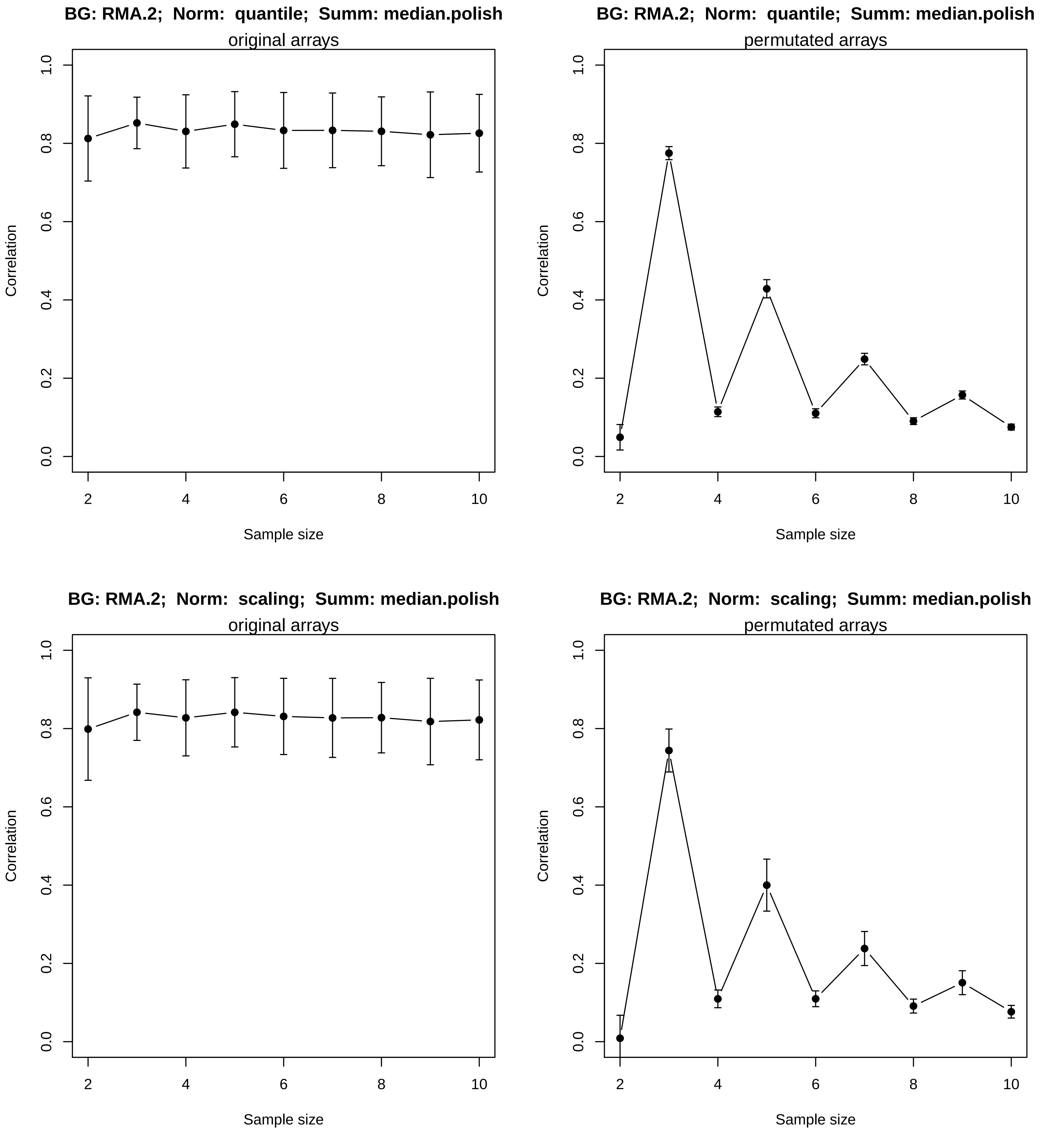

BG: RMA.2; Norm: quantile; Summ: median.polish
original arrays
BG: RMA.2; Norm: quantile; Summ: median.polish
permutated arrays
BG: RMA.2; Norm: scaling; Summ: median.polish
original arrays
BG: RMA.2; Norm: scaling; Summ: median.polish
permutated arrays
Correlation
Sample size
0.0
0.2
0.4
0.6
0.8
1.0
2
4
6
8
10

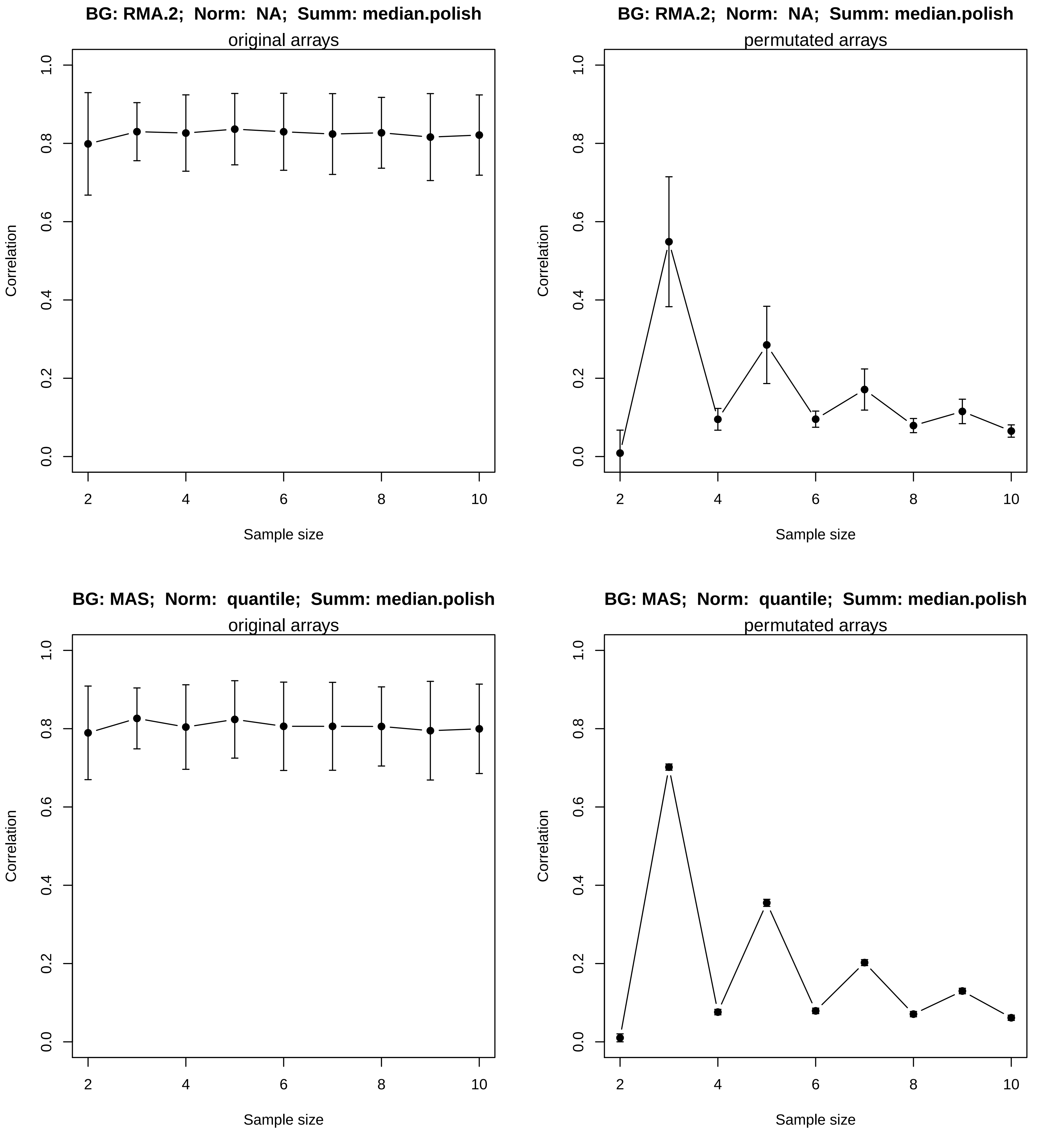
BG: RMA.2; Norm: NA; Summ: median.polish
original arrays
BG: RMA.2; Norm: NA; Summ: median.polish
permutated arrays
BG: MAS; Norm: quantile; Summ: median.polish
original arrays
BG: MAS; Norm: quantile; Summ: median.polish
permutated arrays
Correlation
Sample size
0.0
0.2
0.4
0.6
0.8
1.0
2
4
6
8
10

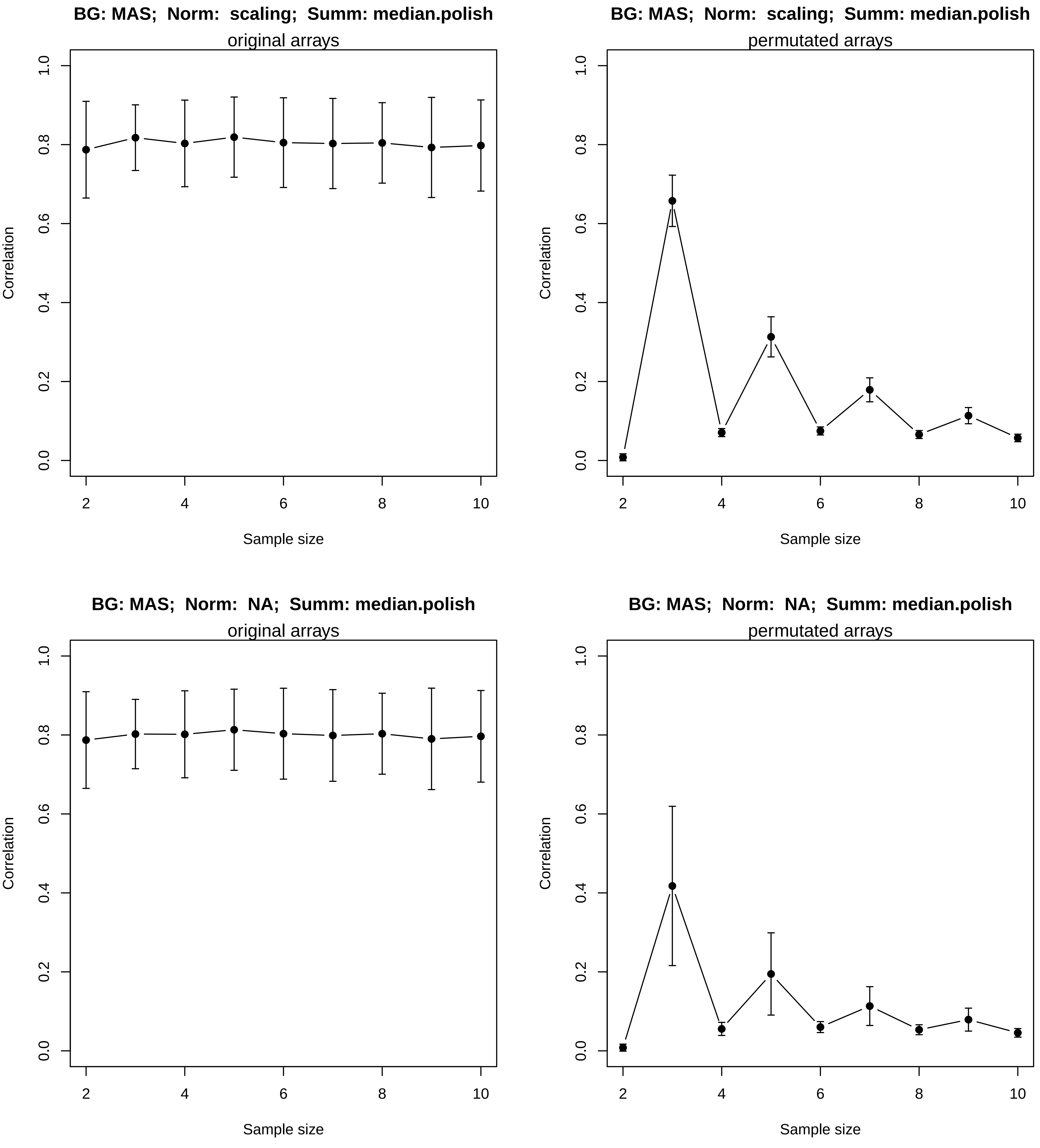
BG: MAS; Norm: scaling; Summ: median.polish
original arrays
Correlation
Sample size
BG: MAS; Norm: scaling; Summ: median.polish
permutated arrays
Correlation
Sample size
BG: MAS; Norm: NA; Summ: median.polish
original arrays
Correlation
Sample size
BG: MAS; Norm: NA; Summ: median.polish
permutated arrays
Correlation
Sample size

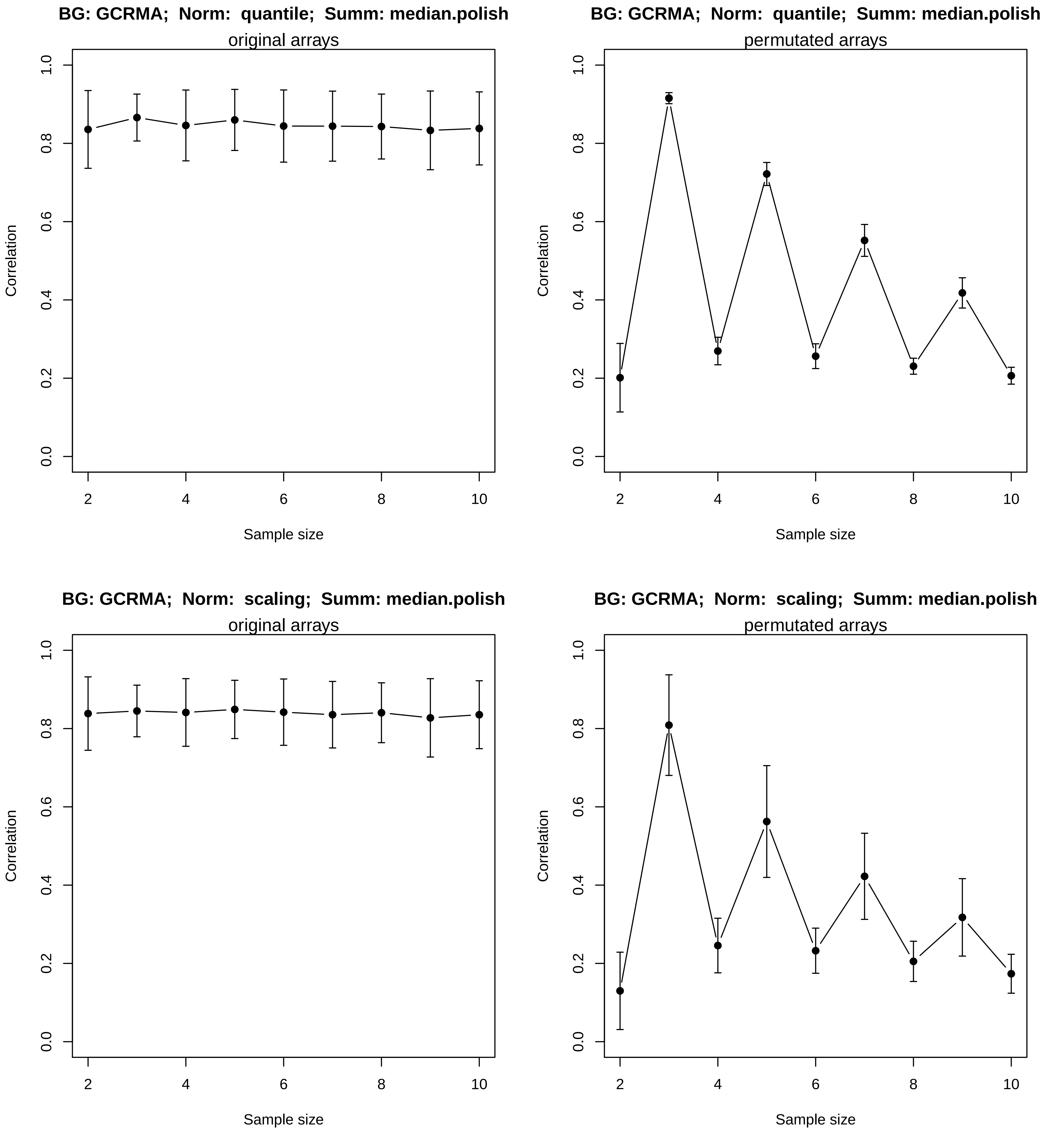
BG: GCRMA; Norm: quantile; Summ: median.polish
original arrays
BG: GCRMA; Norm: quantile; Summ: median.polish
permutated arrays
BG: GCRMA; Norm: scaling; Summ: median.polish
original arrays
BG: GCRMA; Norm: scaling; Summ: median.polish
permutated arrays
Correlation
Sample size
0.0
0.2
0.4
0.6
0.8
1.0
2
4
6
8
10

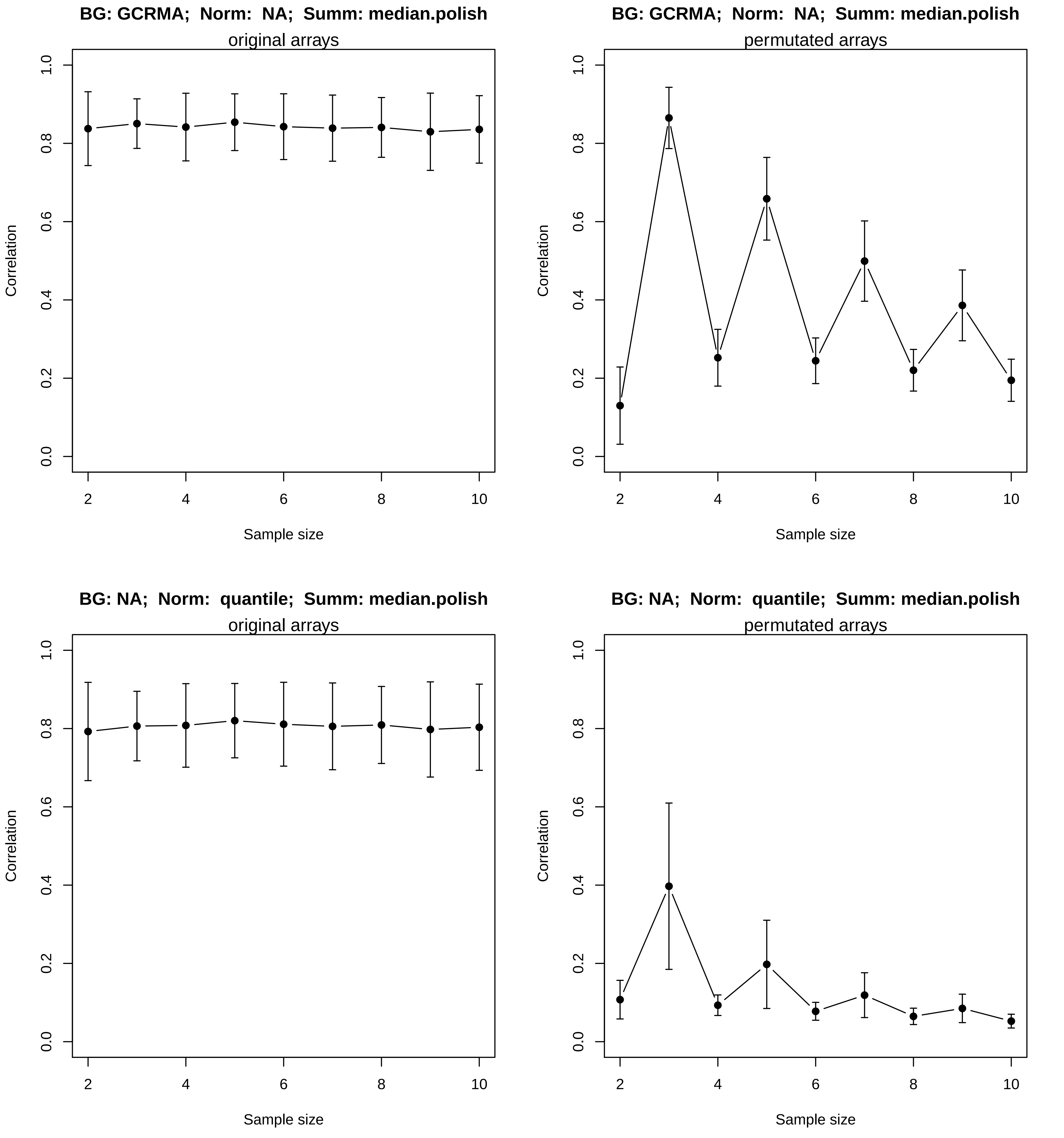
BG: GCRMA; Norm: NA; Summ: median.polish
original arrays
permutated arrays
BG: NA; Norm: quantile; Summ: median.polish
original arrays
permutated arrays
Correlation
Sample size
0.0
0.2
0.4
0.6
0.8
1.0
2
4
6
8
10

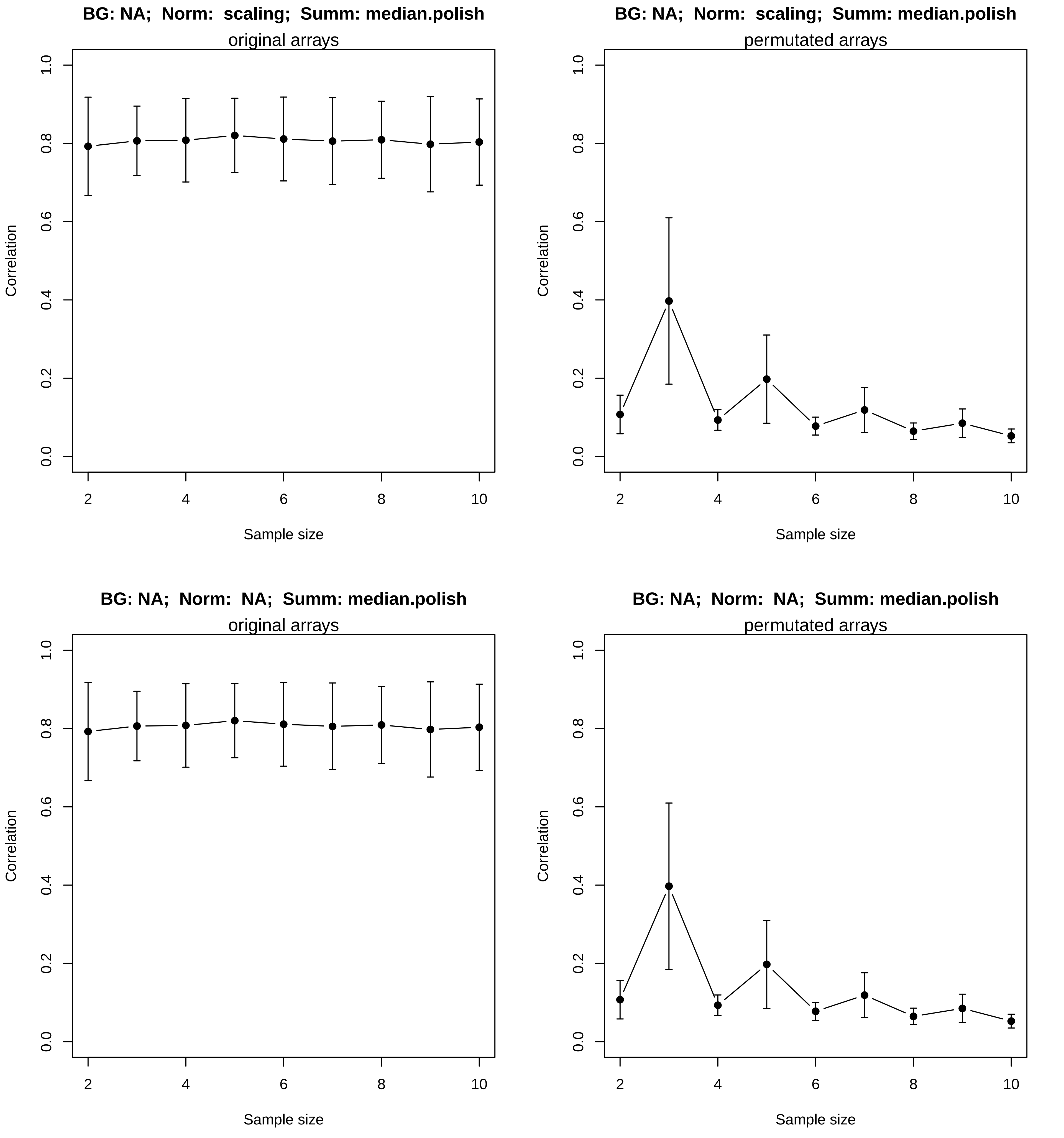
BG: NA; Norm: scaling; Summ: median.polish
original arrays
Correlation
Sample size
BG: NA; Norm: scaling; Summ: median.polish
permutated arrays
Correlation
Sample size
BG: NA; Norm: NA; Summ: median.polish
original arrays
Correlation
Sample size
BG: NA; Norm: NA; Summ: median.polish
permutated arrays
Correlation
Sample size

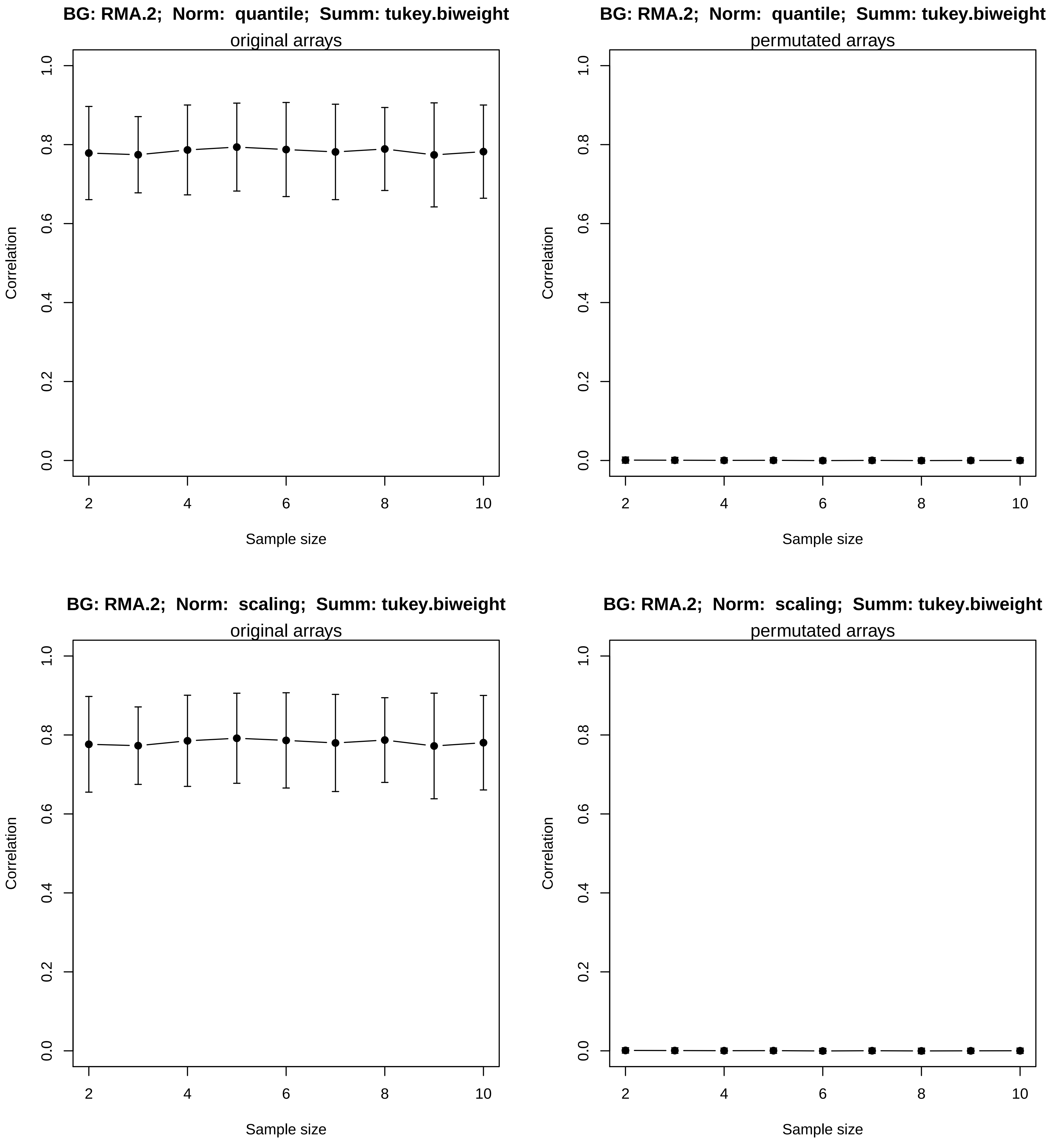
BG: RMA.2; Norm: quantile; Summ: tukey.biweight
original arrays
Correlation
Sample size
BG: RMA.2; Norm: quantile; Summ: tukey.biweight
permutated arrays
Correlation
Sample size
BG: RMA.2; Norm: scaling; Summ: tukey.biweight
original arrays
Correlation
Sample size
BG: RMA.2; Norm: scaling; Summ: tukey.biweight
permutated arrays
Correlation
Sample size

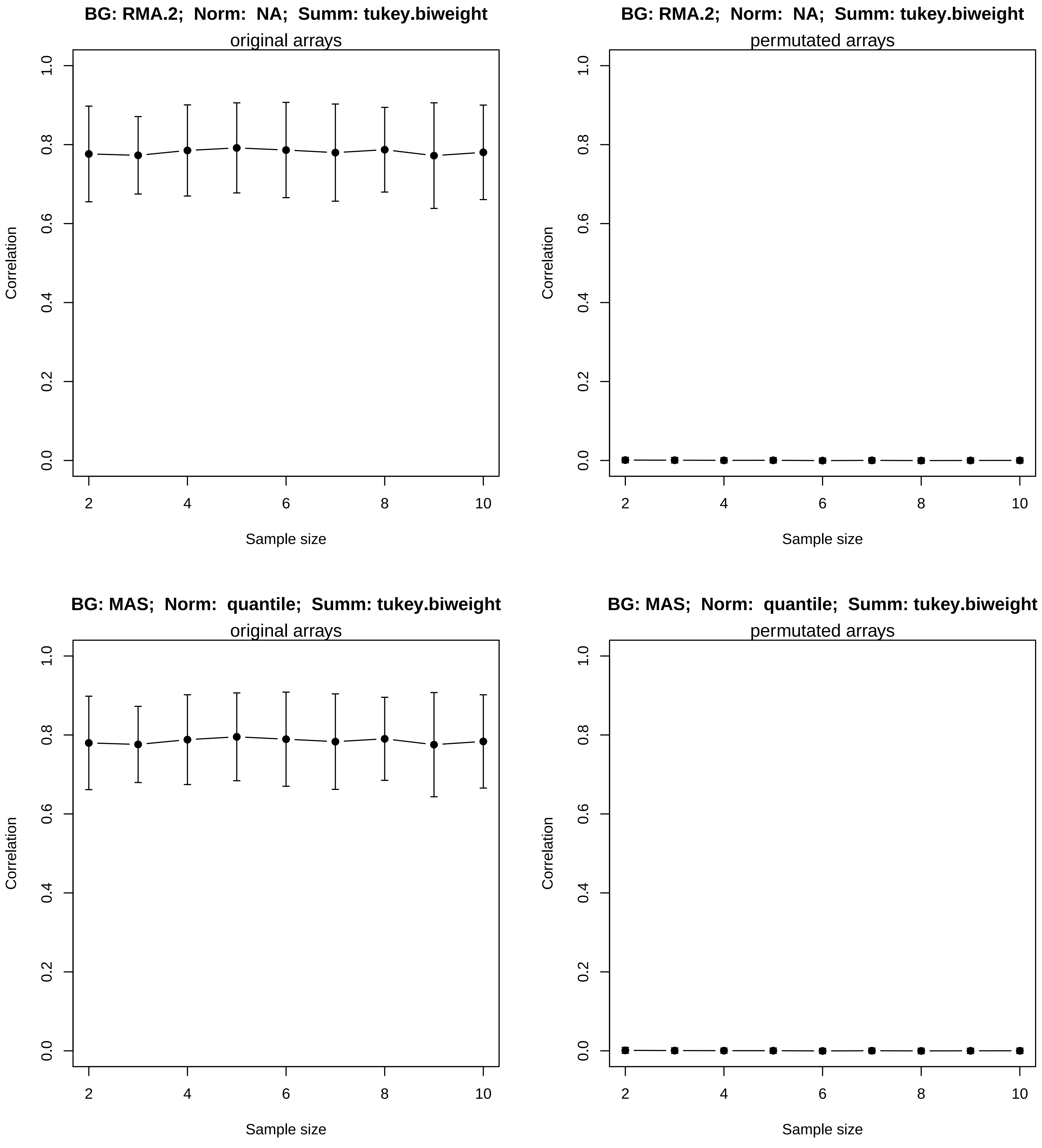
BG: RMA.2; Norm: NA; Summ: tukey.biweight
original arrays
BG: RMA.2; Norm: NA; Summ: tukey.biweight
permutated arrays
BG: MAS; Norm: quantile; Summ: tukey.biweight
original arrays
BG: MAS; Norm: quantile; Summ: tukey.biweight
permutated arrays
Correlation
Sample size
0.0
0.2
0.4
0.6
0.8
1.0
2
4
6
8
10

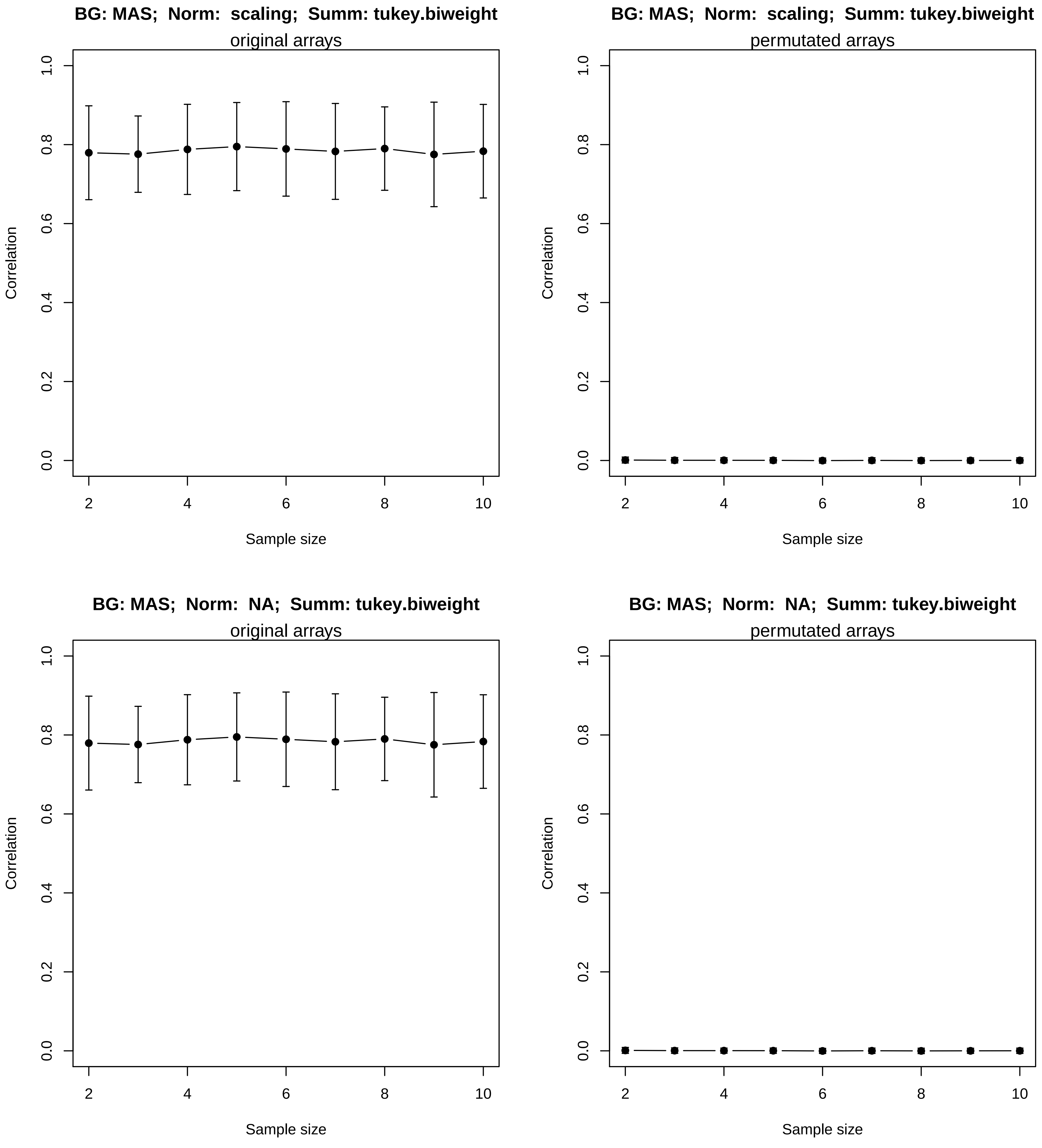
BG: MAS; Norm: scaling; Summ: tukey.biweight
original arrays
Correlation
Sample size
BG: MAS; Norm: scaling; Summ: tukey.biweight
permutated arrays
Correlation
Sample size
BG: MAS; Norm: NA; Summ: tukey.biweight
original arrays
Correlation
Sample size
BG: MAS; Norm: NA; Summ: tukey.biweight
permutated arrays
Correlation
Sample size

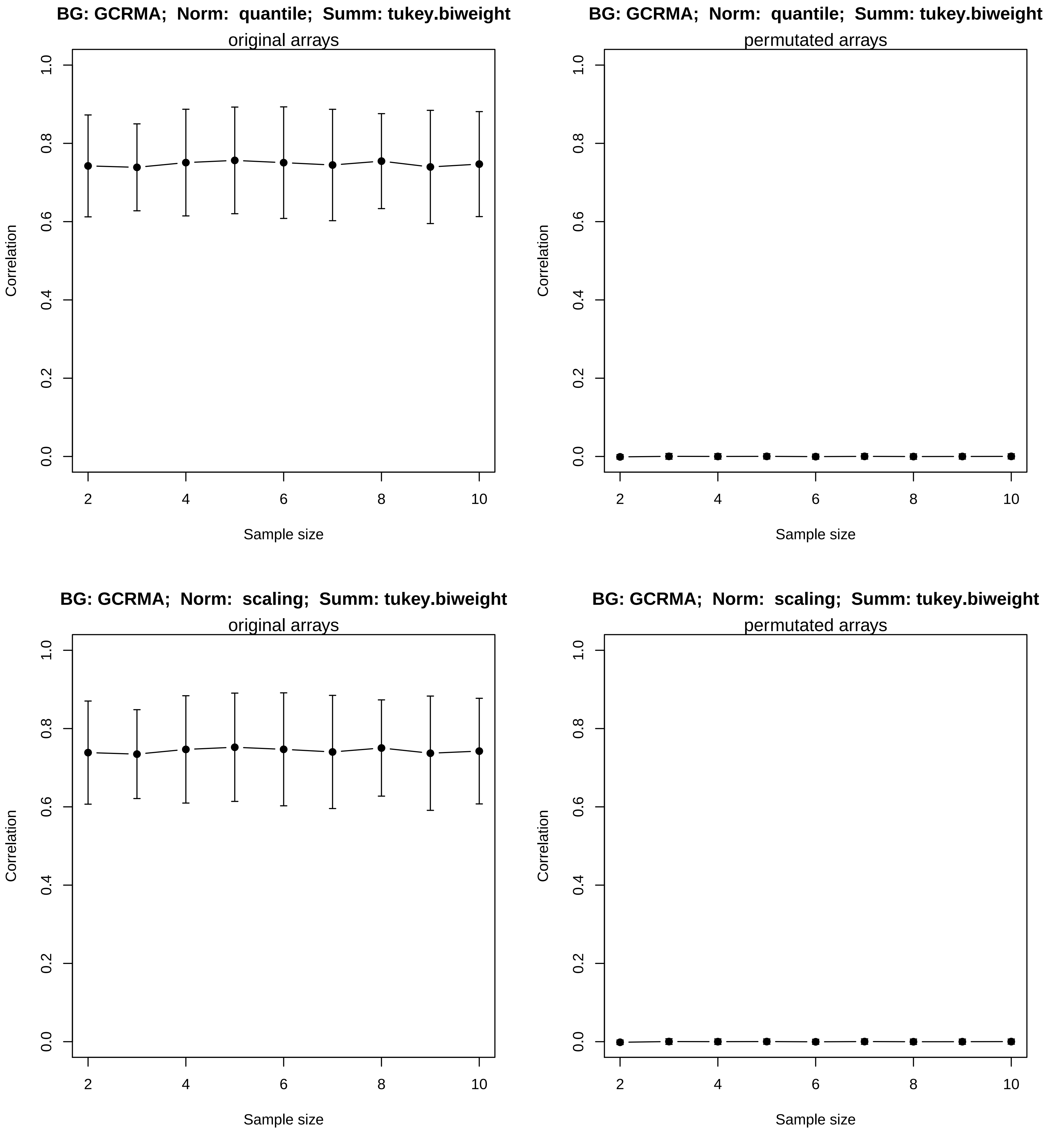
BG: GCRMA; Norm: quantile; Summ: tukey.biweight
original arrays
permutated arrays
BG: GCRMA; Norm: scaling; Summ: tukey.biweight
Correlation
Sample size
0.0
0.2
0.4
0.6
0.8
1.0
2
4
6
8
10

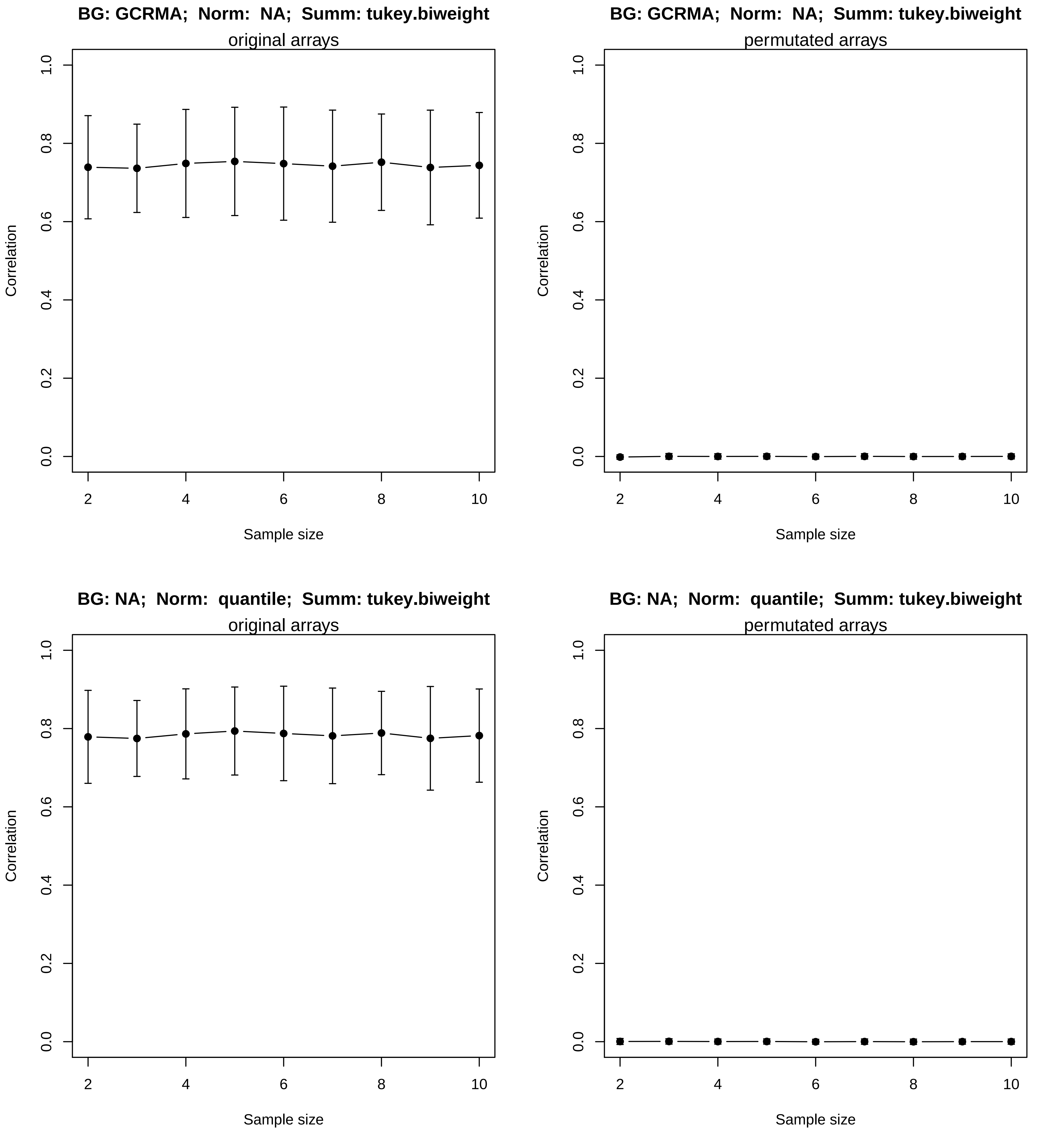
BG: GCRMA; Norm: NA; Summ: tukey.biweight
original arrays
permutated arrays
BG: NA; Norm: quantile; Summ: tukey.biweight
original arrays
permutated arrays
Correlation
Sample size
0.0
0.2
0.4
0.6
0.8
1.0
2
4
6
8
10

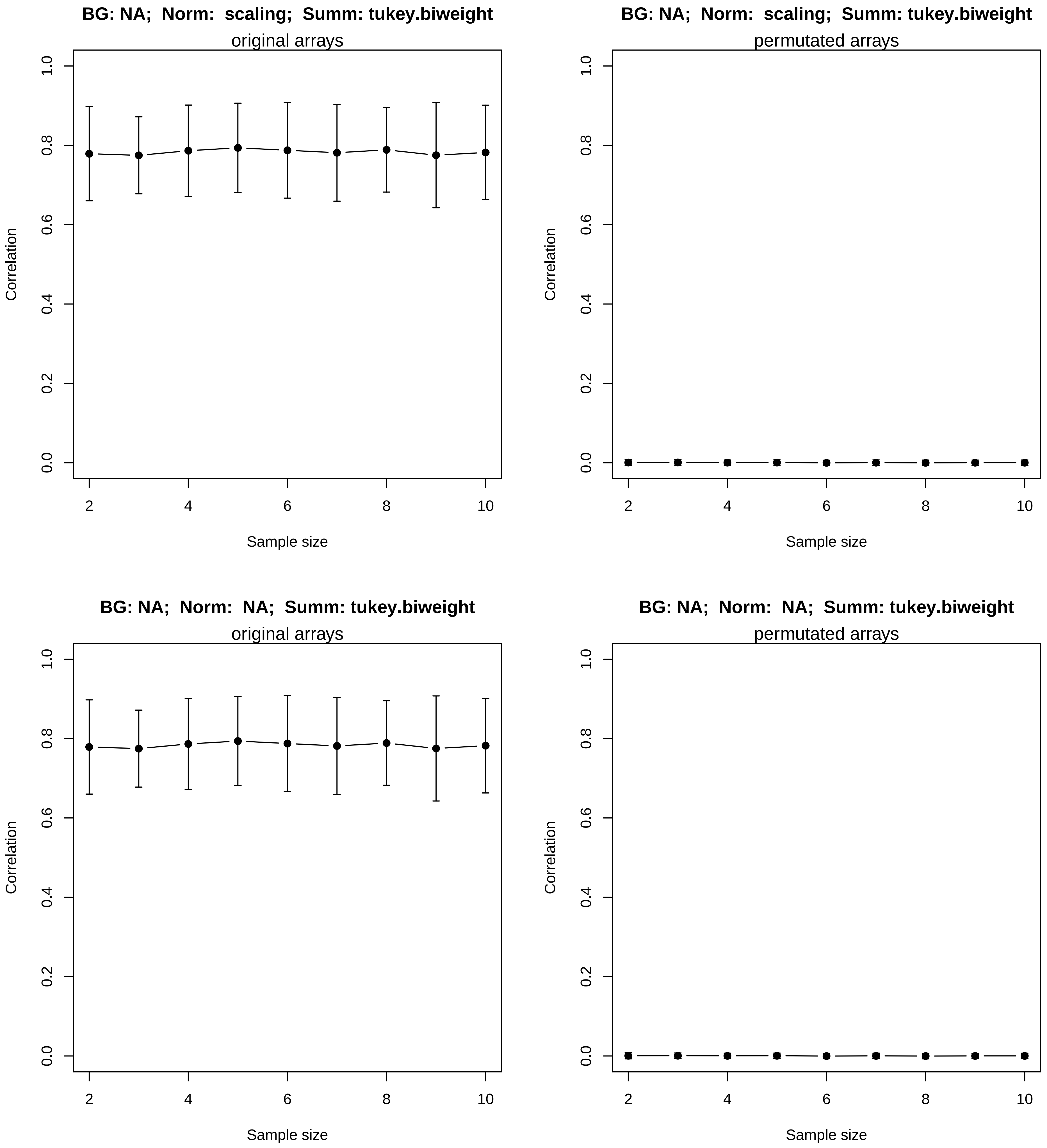

BG: NA; Norm: scaling; Summ: tukey.biweight
original arrays
Correlation
Sample size
BG: NA; Norm: scaling; Summ: tukey.biweight
permutated arrays
Correlation
Sample size
BG: NA; Norm: NA; Summ: tukey.biweight
original arrays
Correlation
Sample size
BG: NA; Norm: NA; Summ: tukey.biweight
permutated arrays
Correlation
Sample size

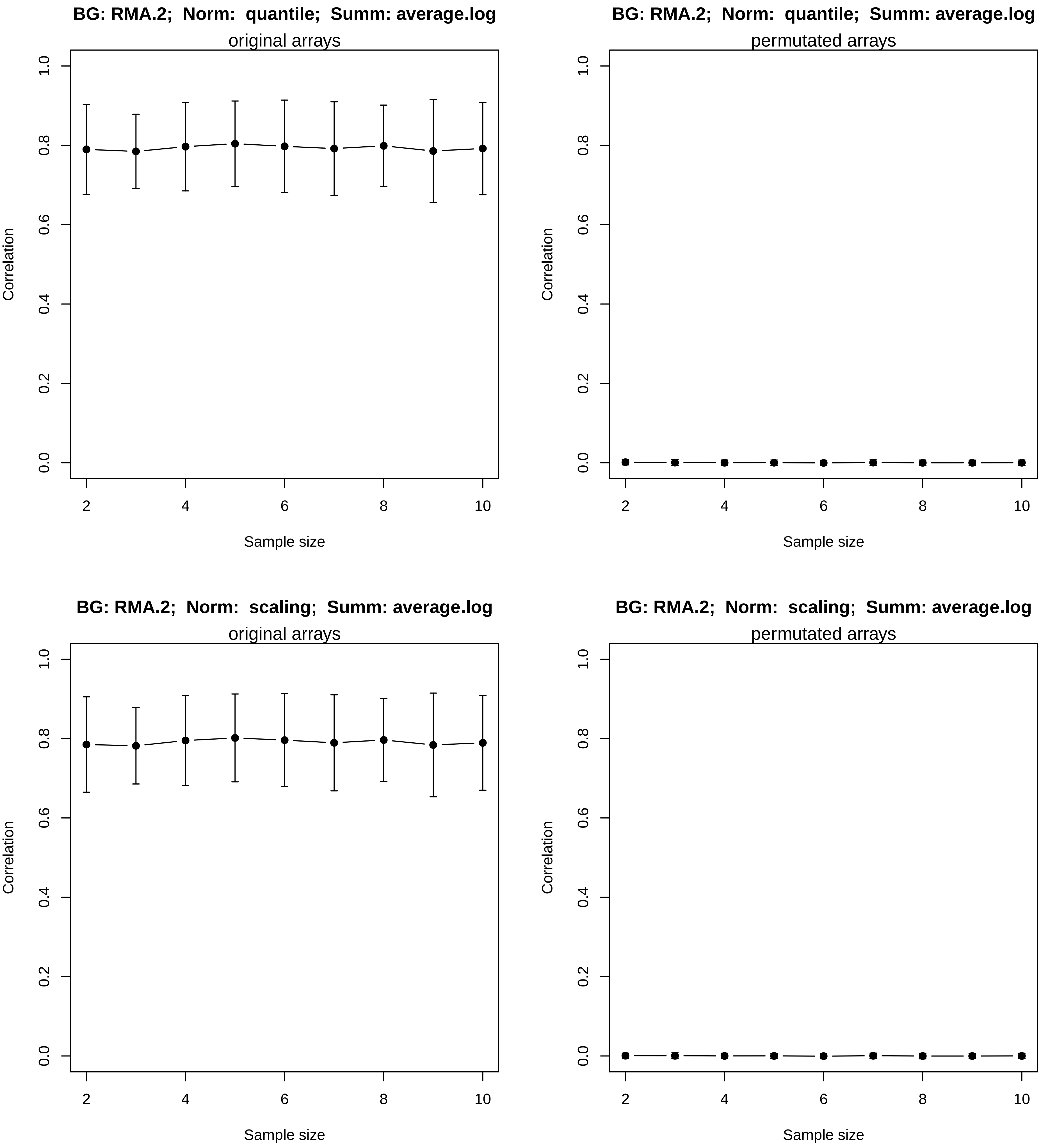
BG: RMA.2; Norm: quantile; Summ: average.log
original arrays
BG: RMA.2; Norm: quantile; Summ: average.log
permutated arrays
BG: RMA.2; Norm: scaling; Summ: average.log
original arrays
BG: RMA.2; Norm: scaling; Summ: average.log
permutated arrays
Correlation
Sample size
0.0
0.2
0.4
0.6
0.8
1.0
2
4
6
8
10

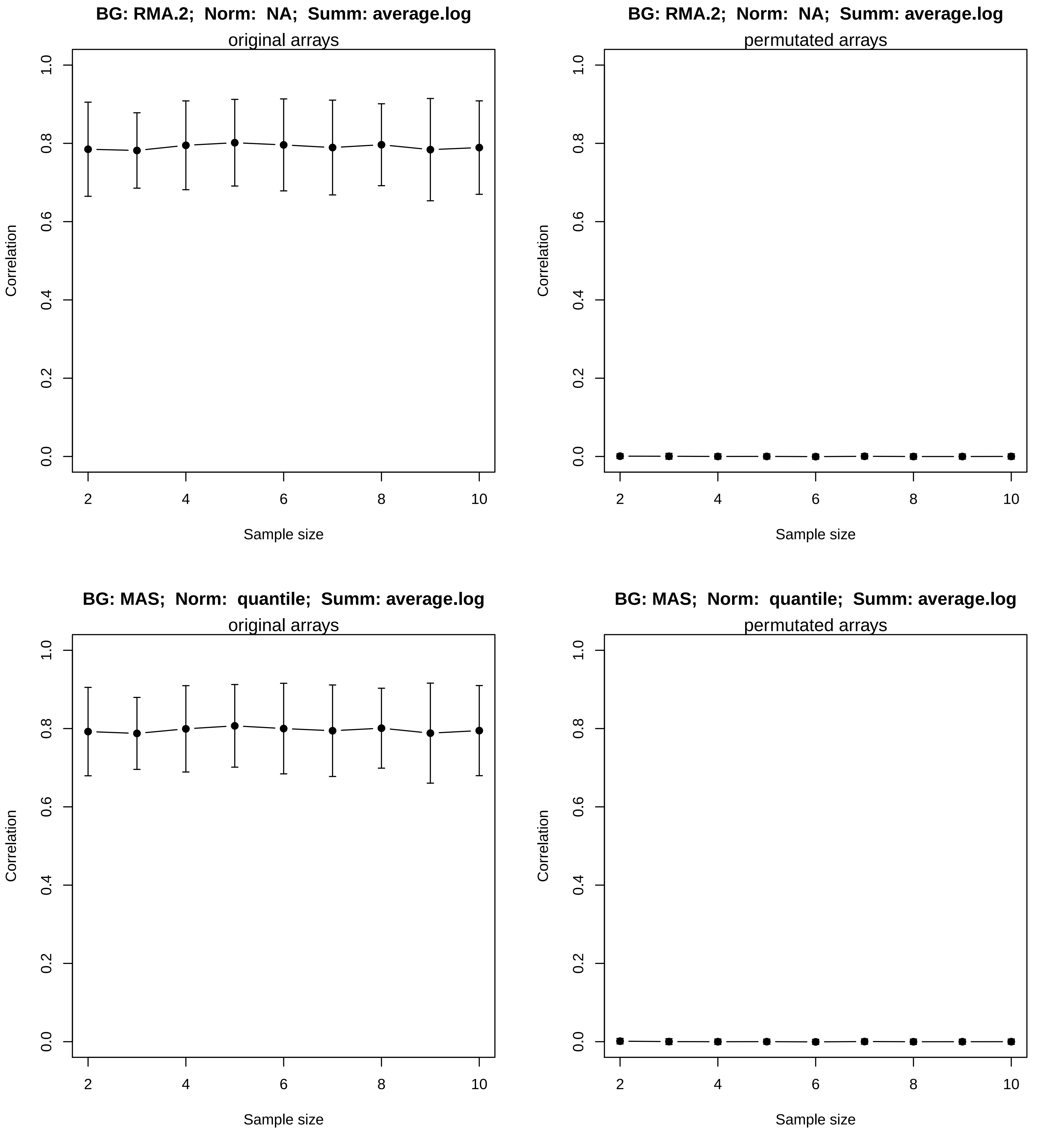
BG: RMA.2; Norm: NA; Summ: average.log
original arrays
Correlation
Sample size
BG: RMA.2; Norm: NA; Summ: average.log
permutated arrays
Correlation
Sample size
BG: MAS; Norm: quantile; Summ: average.log
original arrays
Correlation
Sample size
BG: MAS; Norm: quantile; Summ: average.log
permutated arrays
Correlation
Sample size

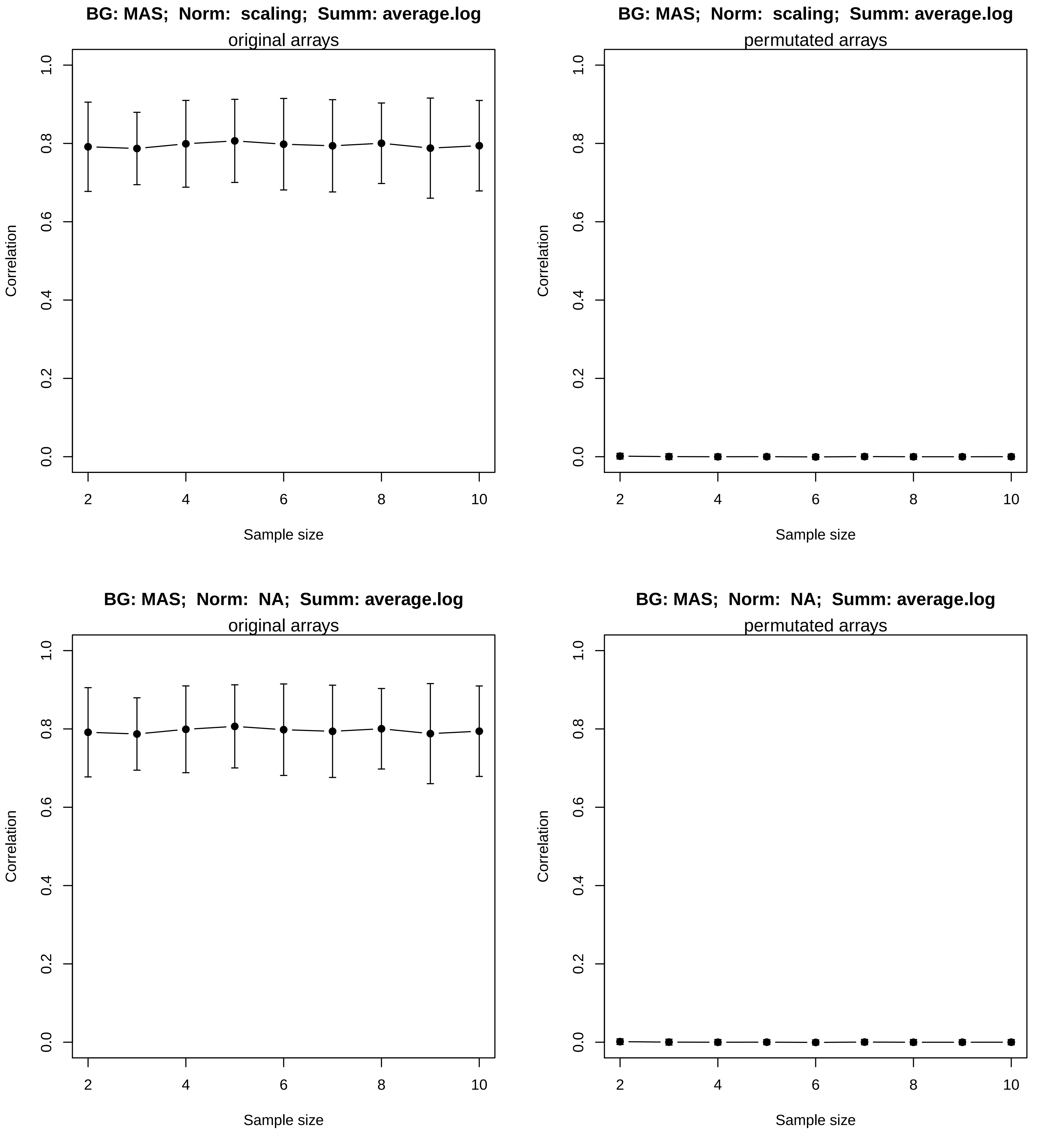
BG: MAS; Norm: scaling; Summ: average.log
original arrays
BG: MAS; Norm: scaling; Summ: average.log
permutated arrays
BG: MAS; Norm: NA; Summ: average.log
original arrays
BG: MAS; Norm: NA; Summ: average.log
permutated arrays
Correlation
Sample size
0.0
0.2
0.4
0.6
0.8
1.0
2
4
6
8
10

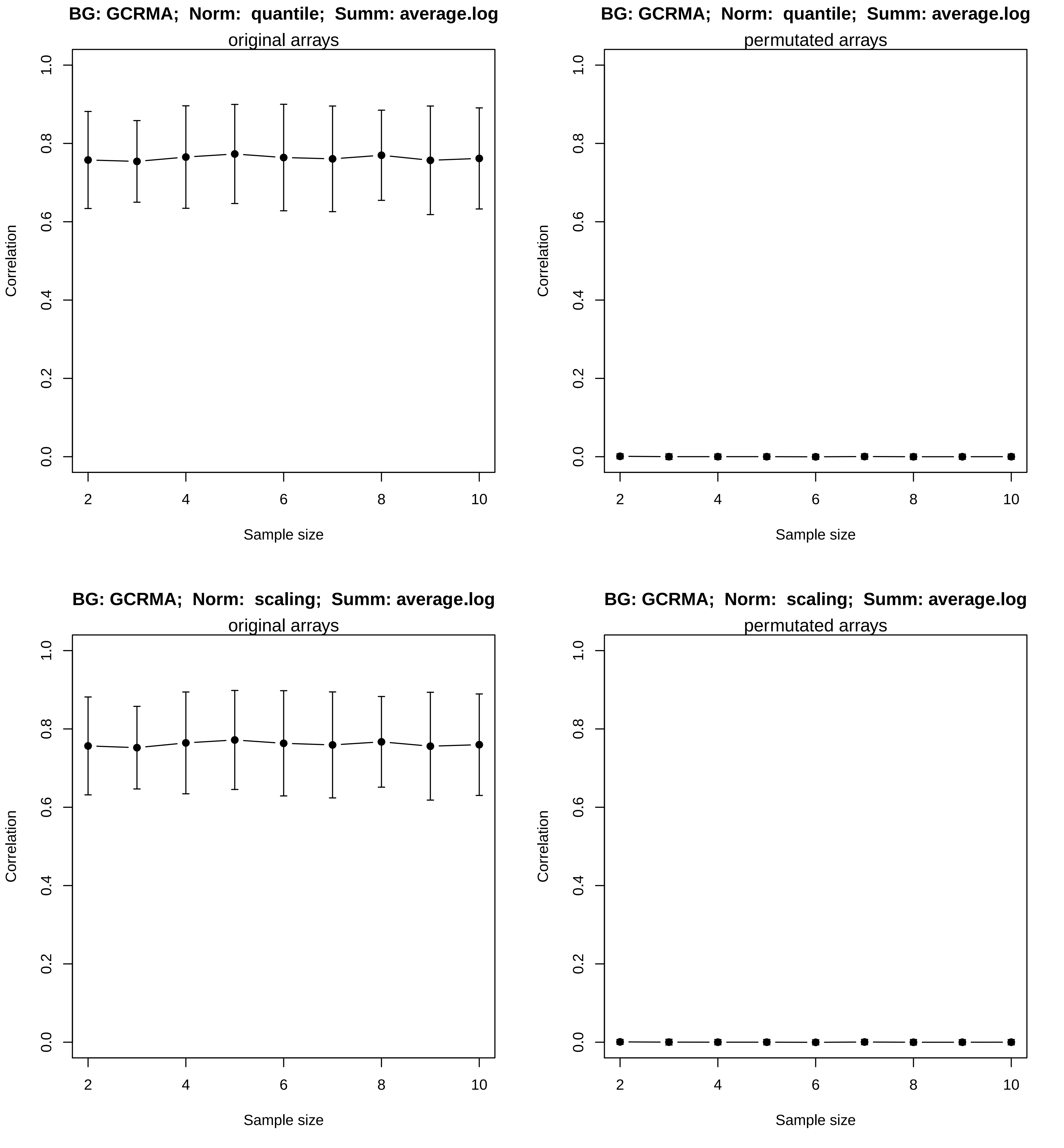
BG: GCRMA; Norm: quantile; Summ: average.log
original arrays
permutated arrays
BG: GCRMA; Norm: scaling; Summ: average.log
original arrays
permutated arrays
Correlation
Sample size
0.0
0.2
0.4
0.6
0.8
1.0
2
4
6
8
10

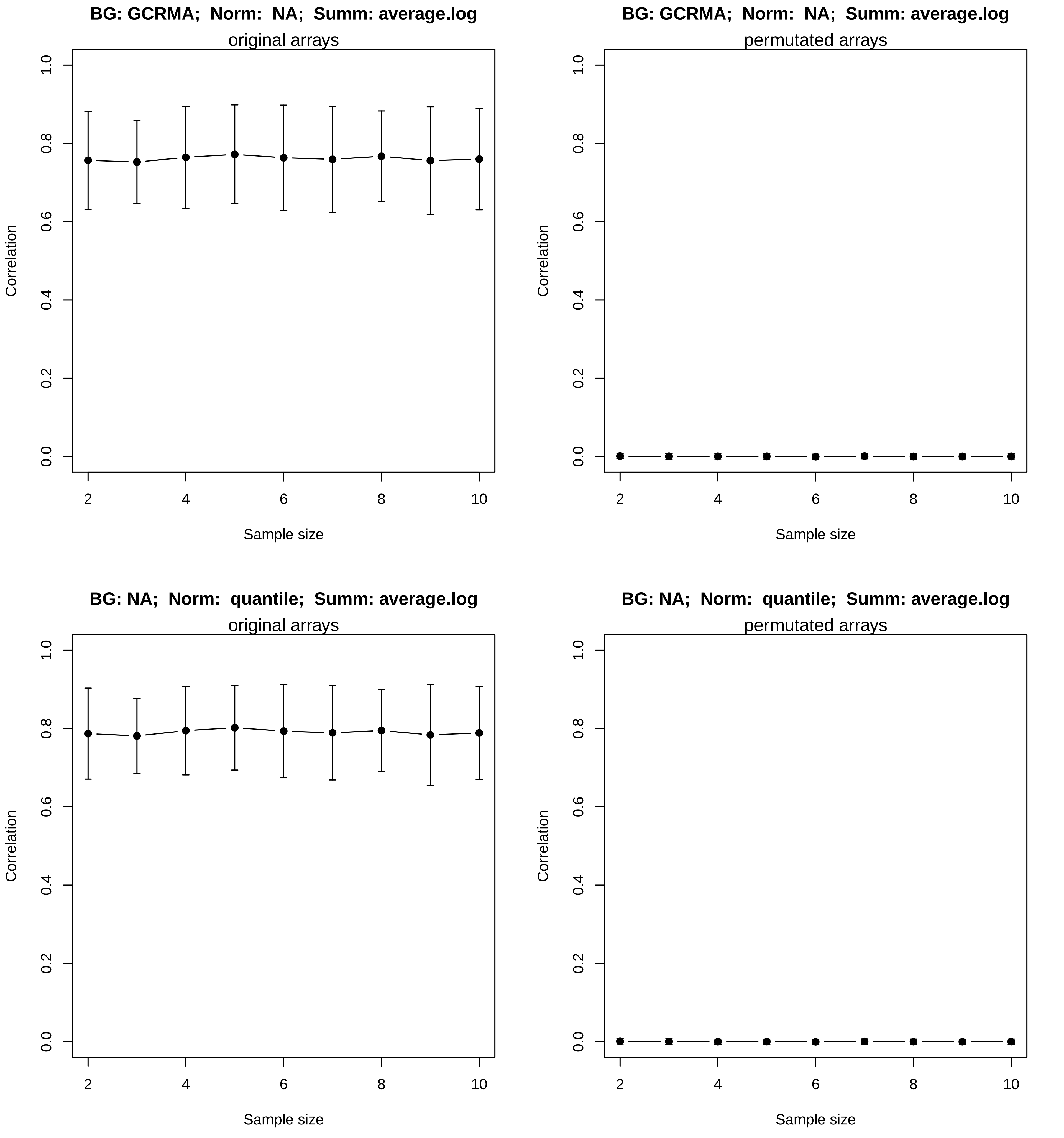
BG: GCRMA; Norm: NA; Summ: average.log
original arrays
permutated arrays
BG: NA; Norm: quantile; Summ: average.log
original arrays
permutated arrays
Correlation
Sample size
0.0
0.2
0.4
0.6
0.8
1.0
2
4
6
8
10

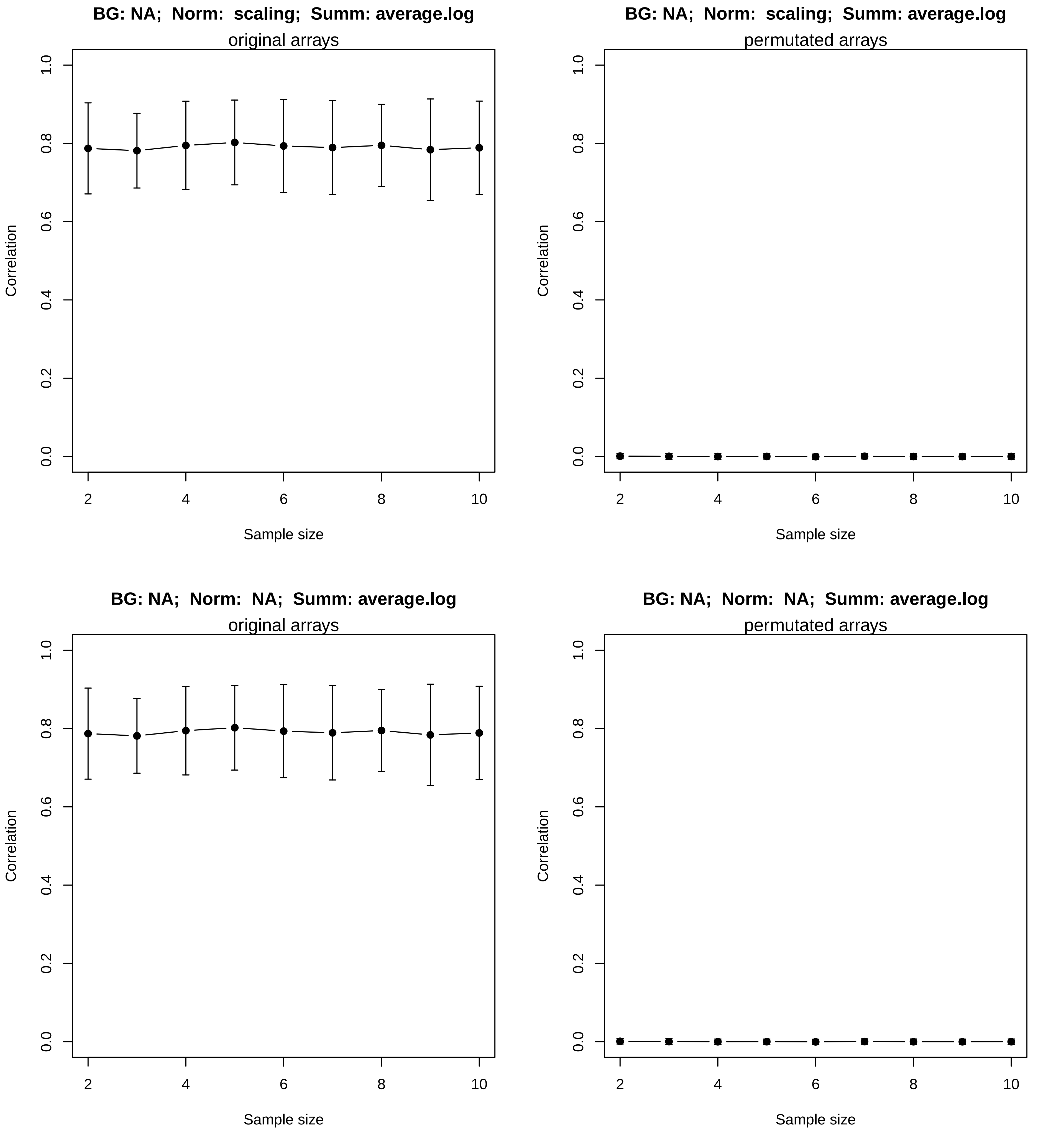
BG: NA; Norm: scaling; Summ: average.log
original arrays
Correlation
Sample size
BG: NA; Norm: scaling; Summ: average.log
permutated arrays
Correlation
Sample size
BG: NA; Norm: NA; Summ: average.log
original arrays
Correlation
Sample size
BG: NA; Norm: NA; Summ: average.log
permutated arrays
Correlation
Sample size

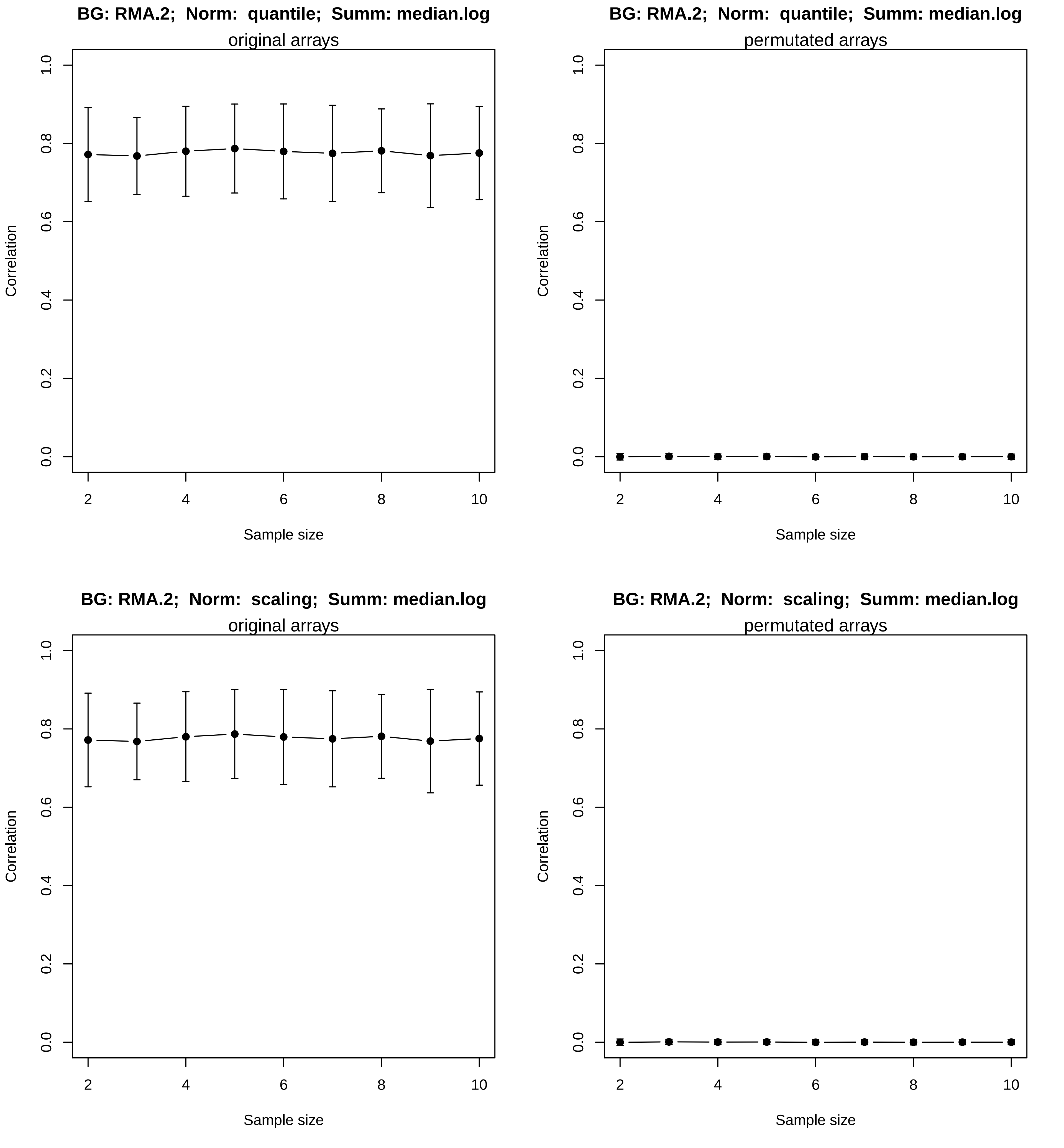
BG: RMA.2; Norm: quantile; Summ: median.log
original arrays
Correlation
Sample size
BG: RMA.2; Norm: quantile; Summ: median.log
permutated arrays
Correlation
Sample size
BG: RMA.2; Norm: scaling; Summ: median.log
original arrays
Correlation
Sample size
BG: RMA.2; Norm: scaling; Summ: median.log
permutated arrays
Correlation
Sample size

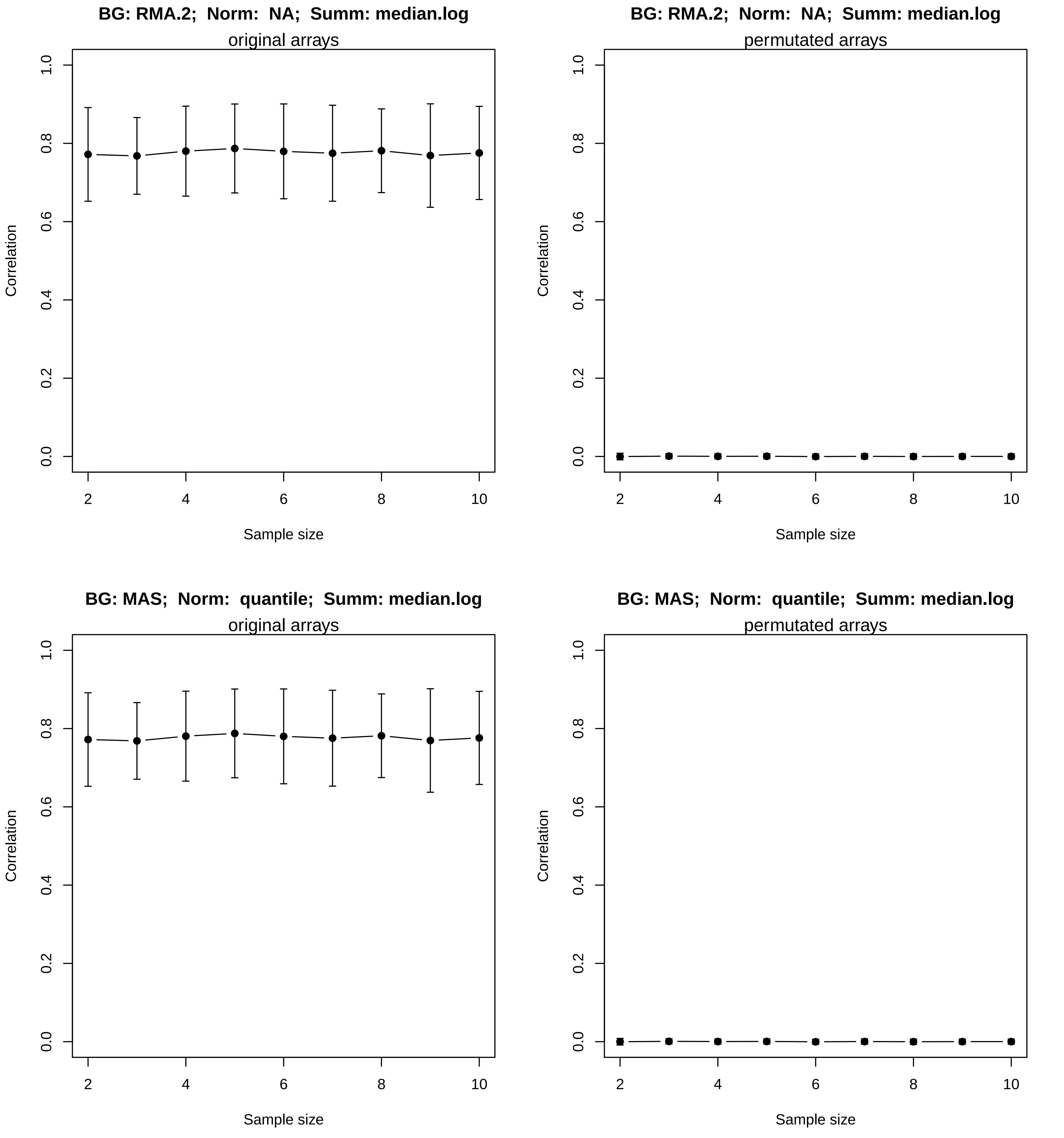
BG: RMA.2; Norm: NA; Summ: median.log
original arrays
Correlation
0.0
0.2
0.4
0.6
0.8
1.0
2
4
6
8
10
Sample size
BG: RMA.2; Norm: NA; Summ: median.log
permutated arrays
Correlation
Sample size
BG: MAS; Norm: quantile; Summ: median.log
original arrays
Correlation
Sample size
BG: MAS; Norm: quantile; Summ: median.log
permutated arrays
Correlation
Sample size

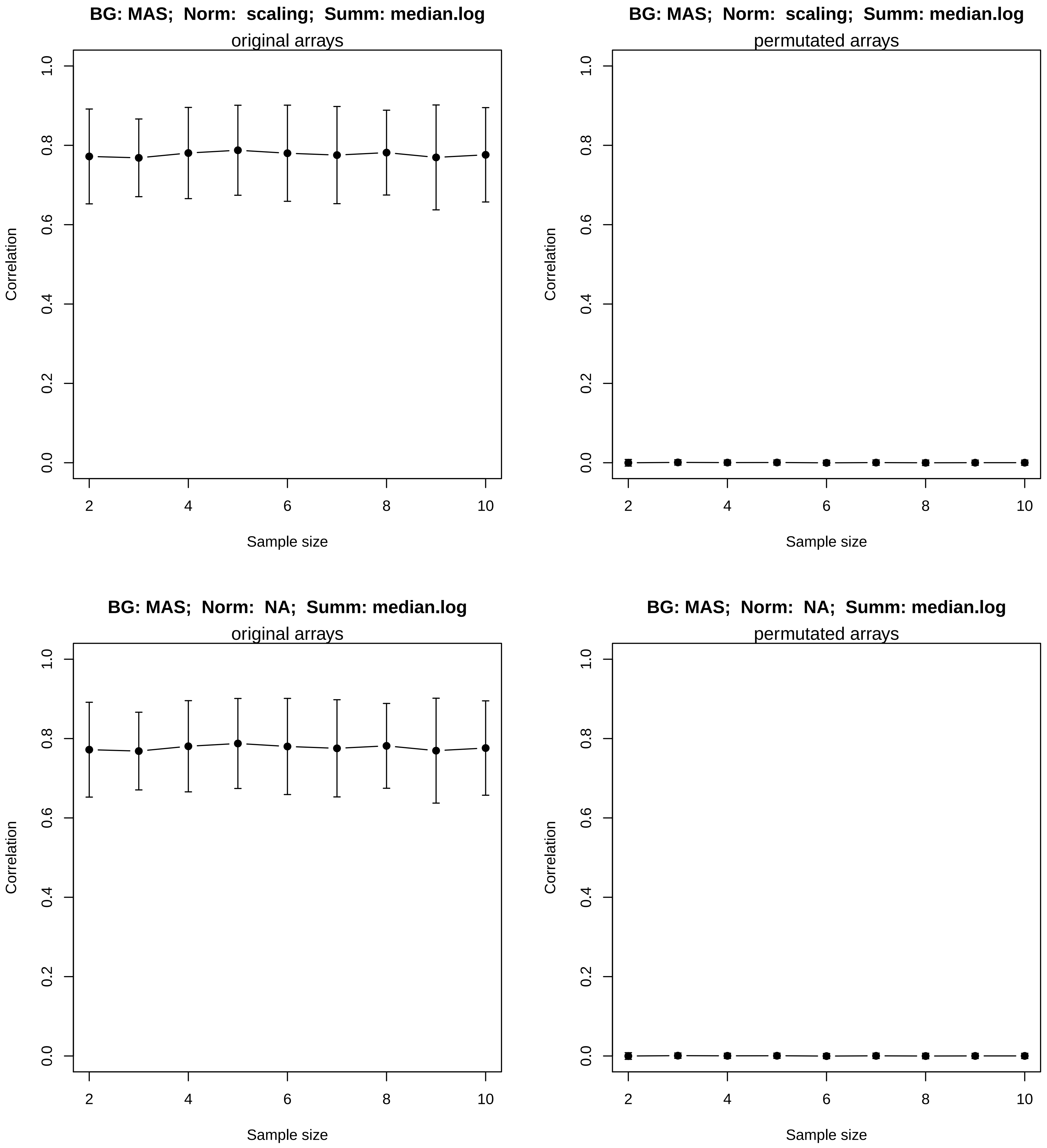
BG: MAS; Norm: scaling; Summ: median.log
original arrays
Correlation
0.0
0.2
0.4
0.6
0.8
1.0
2
4
6
8
10
Sample size
BG: MAS; Norm: scaling; Summ: median.log
permutated arrays
Correlation
Sample size
BG: MAS; Norm: NA; Summ: median.log
original arrays
Correlation
Sample size
BG: MAS; Norm: NA; Summ: median.log
permutated arrays
Correlation
Sample size

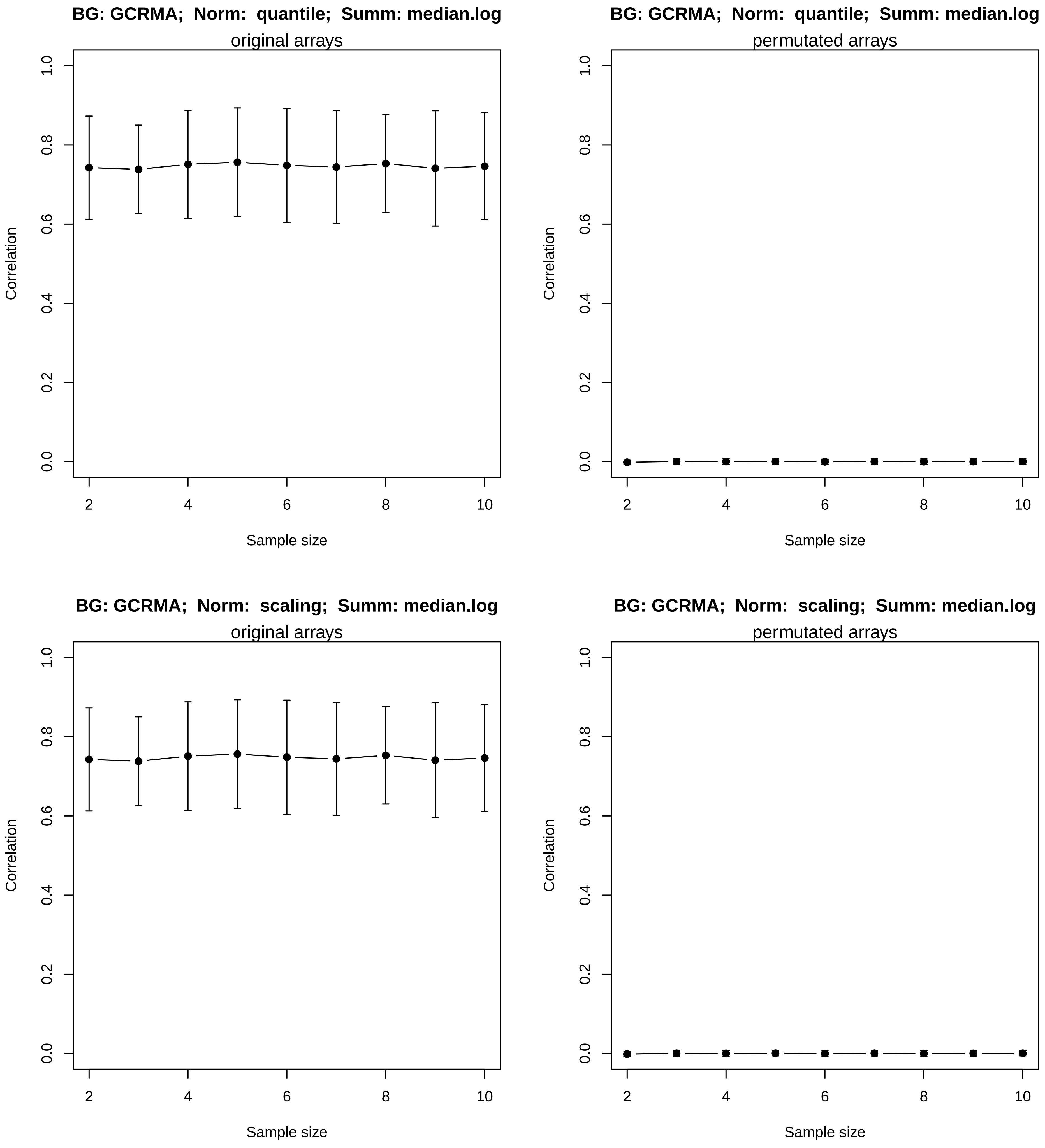
BG: GCRMA; Norm: quantile; Summ: median.log
original arrays
BG: GCRMA; Norm: quantile; Summ: median.log
permutated arrays
BG: GCRMA; Norm: scaling; Summ: median.log
original arrays
BG: GCRMA; Norm: scaling; Summ: median.log
permutated arrays
Correlation
Sample size
0.0
0.2
0.4
0.6
0.8
1.0
2
4
6
8
10

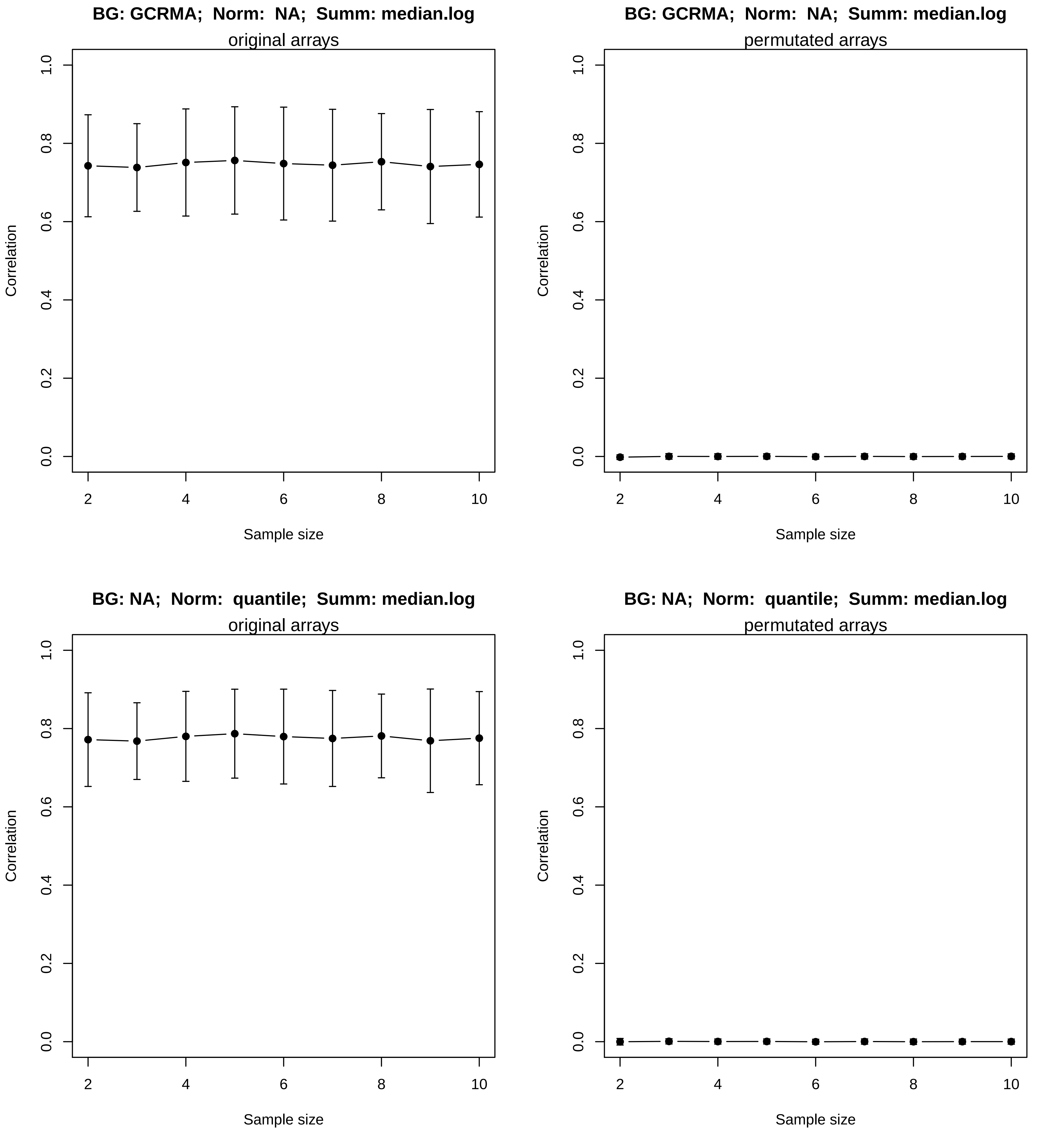

BG: GCRMA; Norm: NA; Summ: median.log
original arrays
BG: GCRMA; Norm: NA; Summ: median.log
permutated arrays
BG: NA; Norm: quantile; Summ: median.log
original arrays
BG: NA; Norm: quantile; Summ: median.log
permutated arrays
Correlation
Sample size

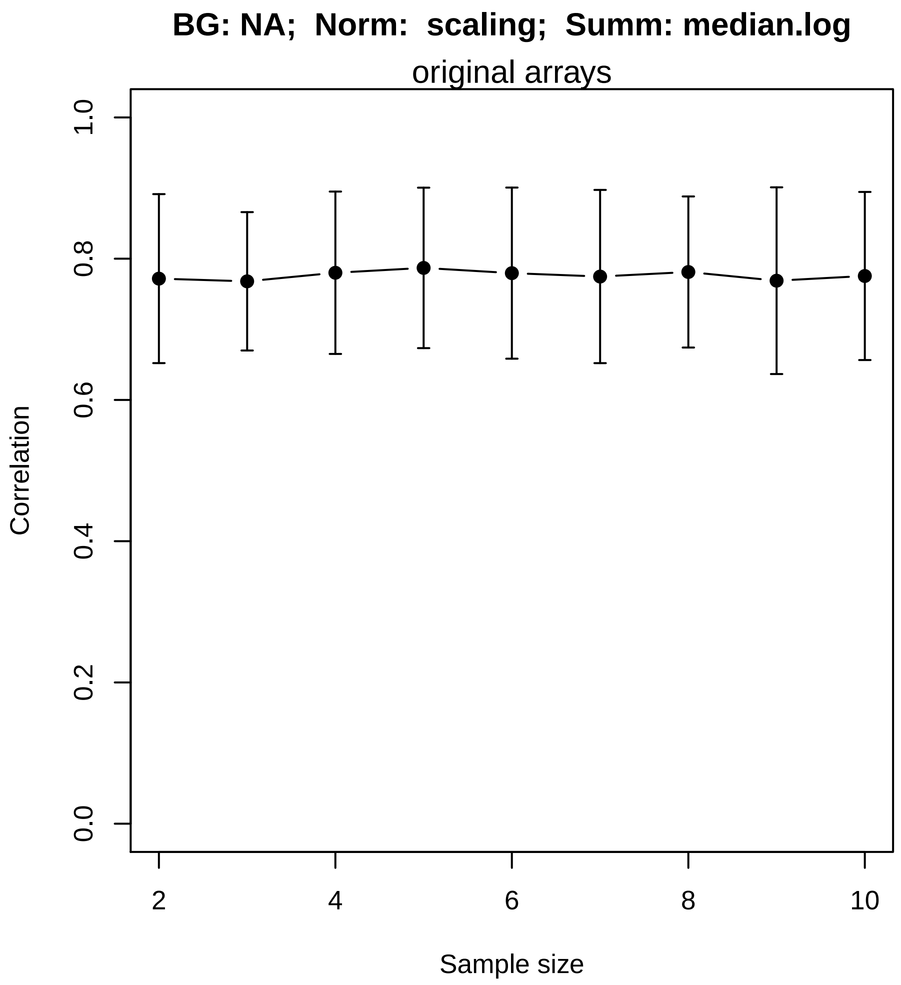
BG: NA; Norm: scaling; Summ: median.log
original arrays
Correlation
Sample size
0.0
0.2
0.4
0.6
0.8
1.0
2
4
6
8
10

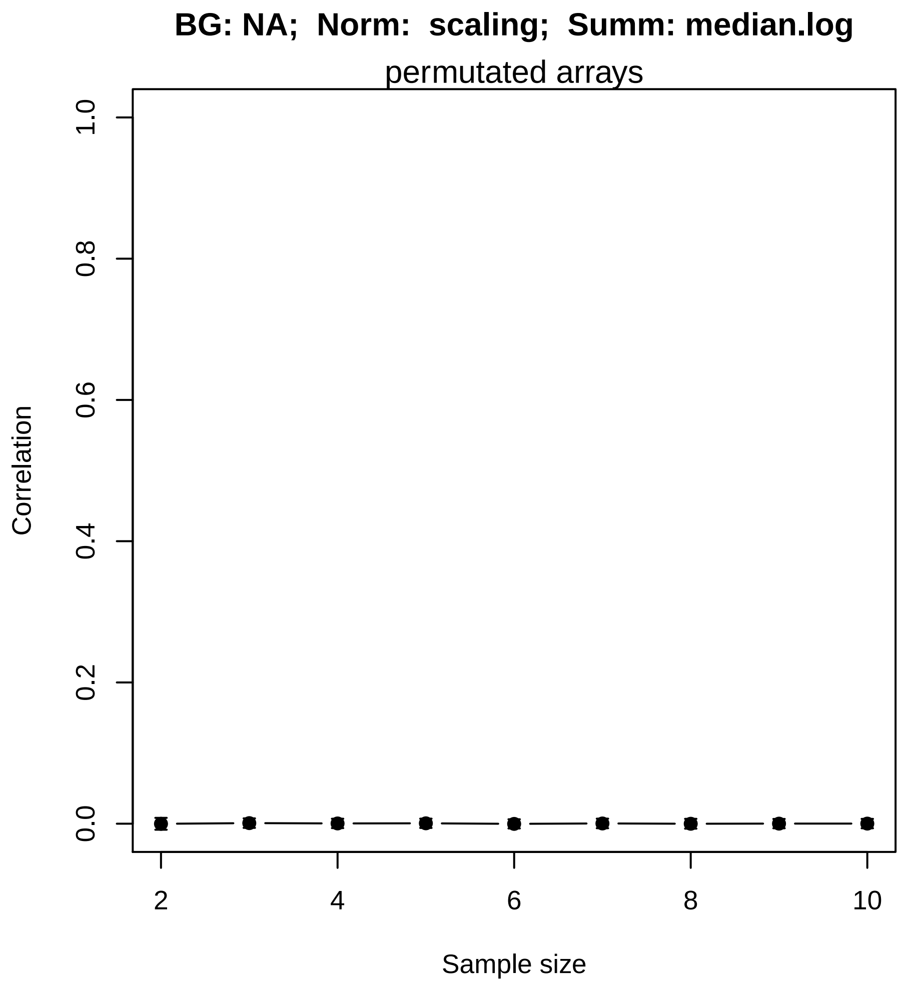
BG: NA; Norm: scaling; Summ: median.log
permutated arrays
Correlation
Sample size
0.0
0.2
0.4
0.6
0.8
1.0
2
4
6
8
10

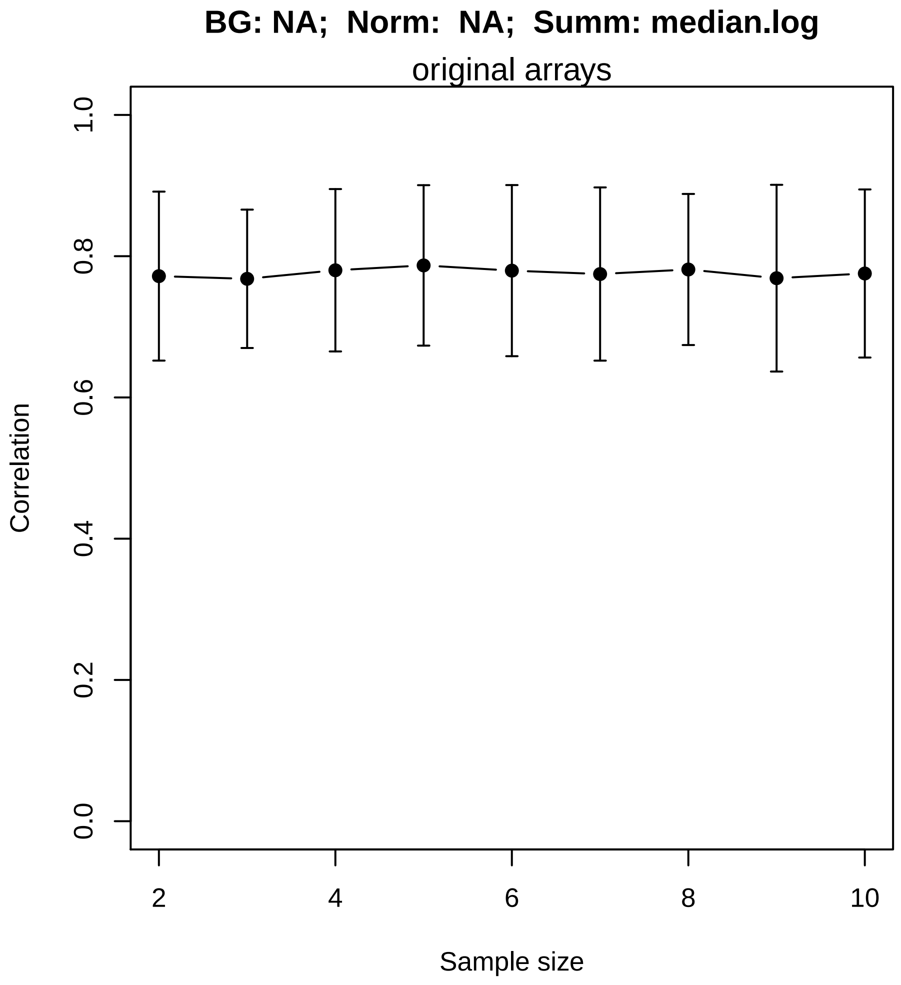
BG: NA; Norm: NA; Summ: median.log
original arrays
Correlation
Sample size
0.0
0.2
0.4
0.6
0.8
1.0
2
4
6
8
10

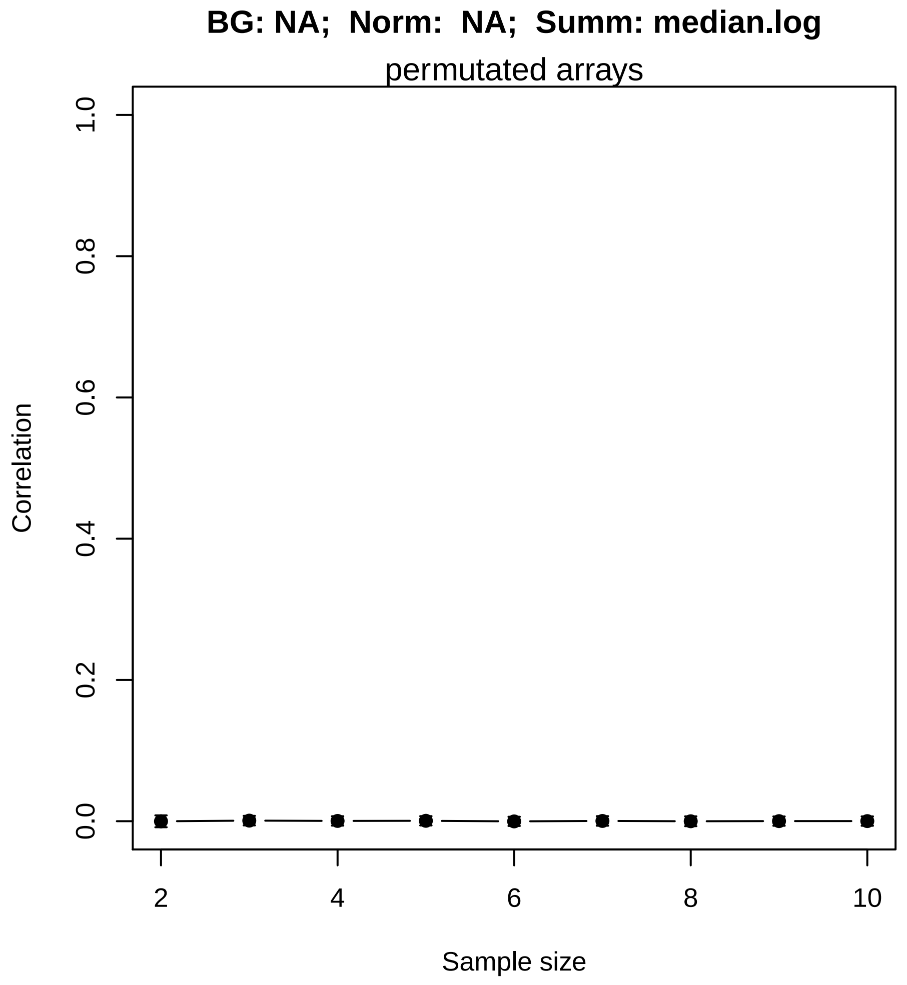
BG: NA; Norm: NA; Summ: median.log
permutated arrays
Correlation
Sample size
0.0
0.2
0.4
0.6
0.8
1.0
2
4
6
8
10

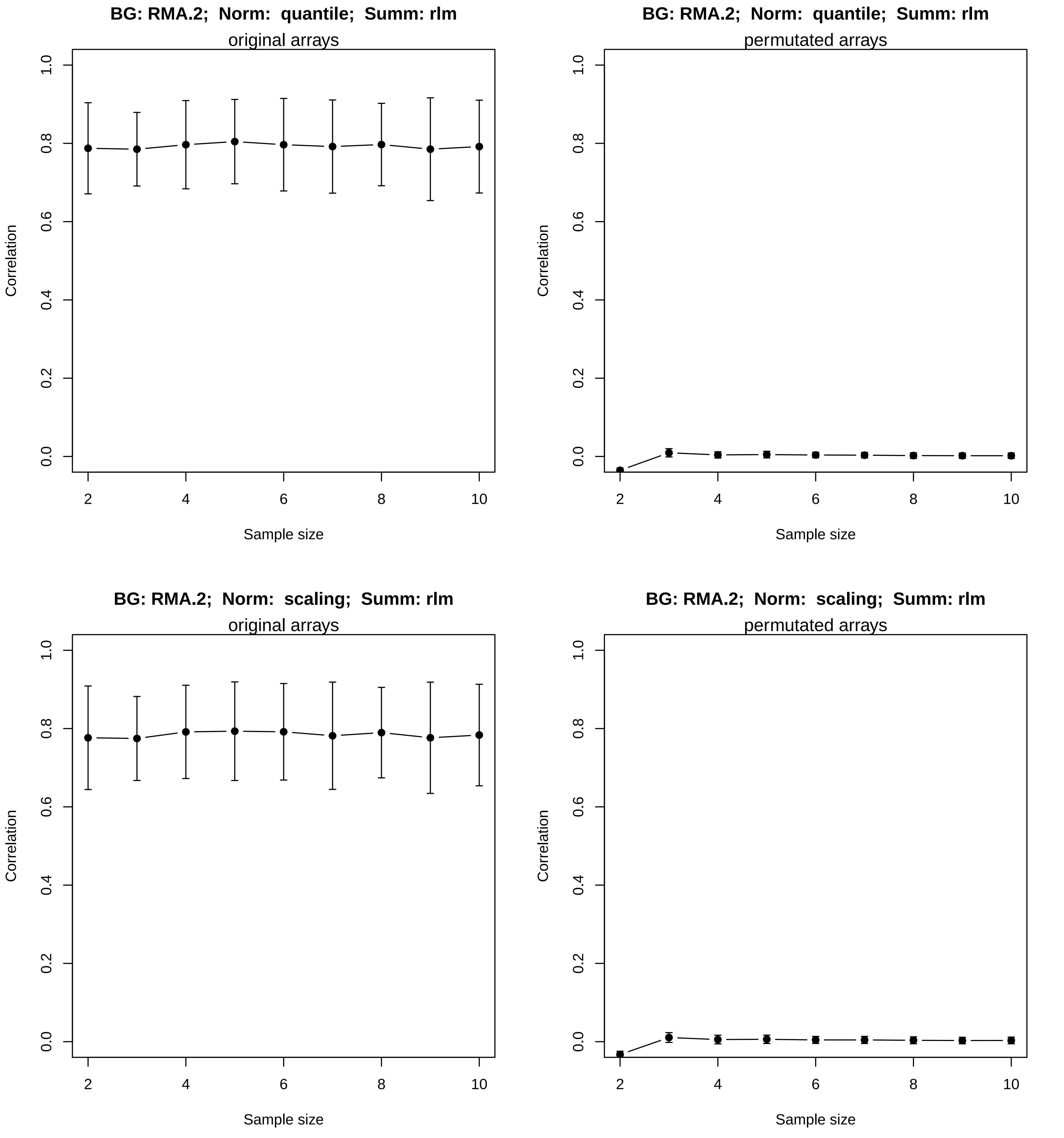
BG: RMA.2; Norm: quantile; Summ: rlm
original arrays
BG: RMA.2; Norm: quantile; Summ: rlm
permutated arrays
BG: RMA.2; Norm: scaling; Summ: rlm
original arrays
BG: RMA.2; Norm: scaling; Summ: rlm
permutated arrays
Correlation
Sample size
0.0
0.2
0.4
0.6
0.8
1.0
2
4
6
8
10

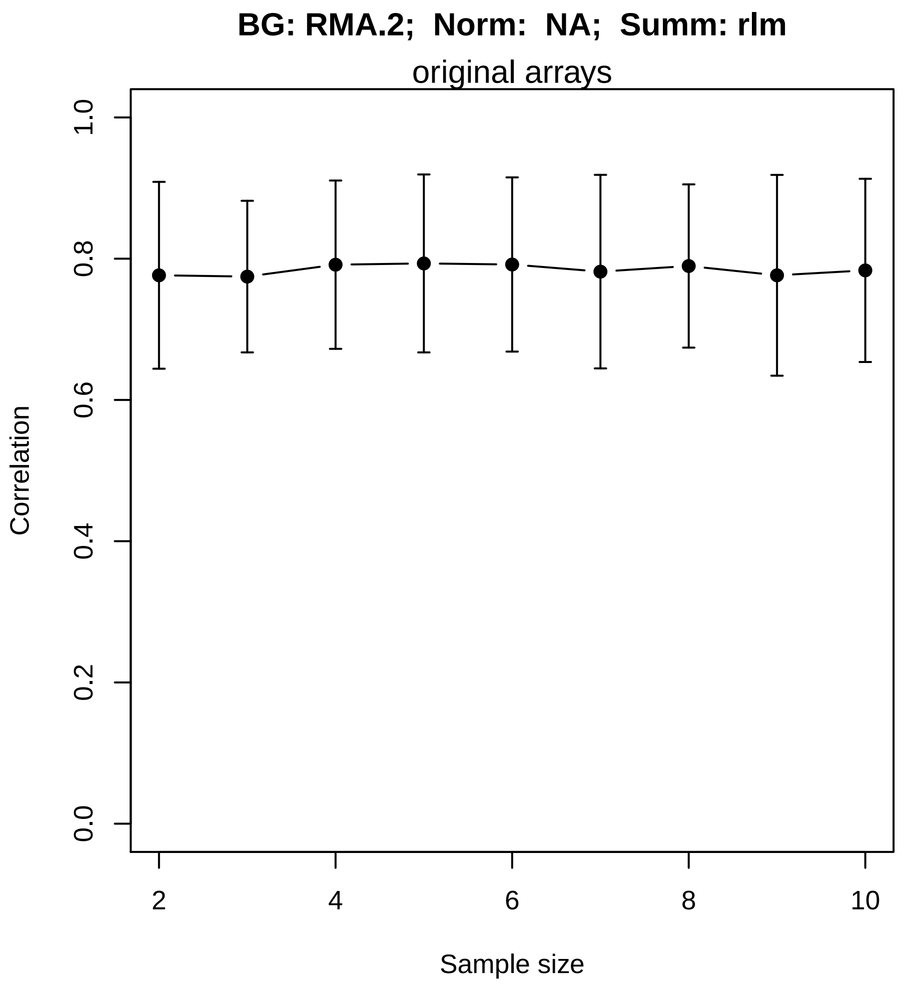

BG: RMA.2; Norm: NA; Summ: rlm
original arrays
Correlation
Sample size

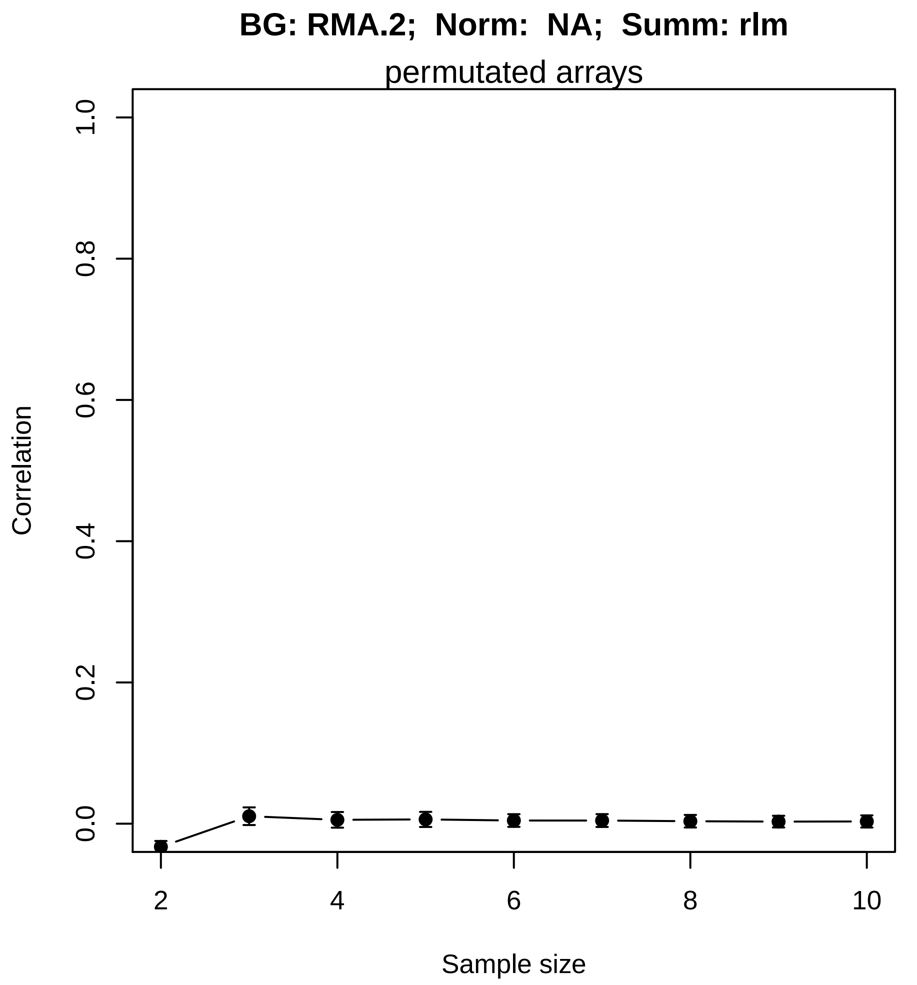

BG: RMA.2; Norm: NA; Summ: rlm
permutated arrays
Correlation
Sample size

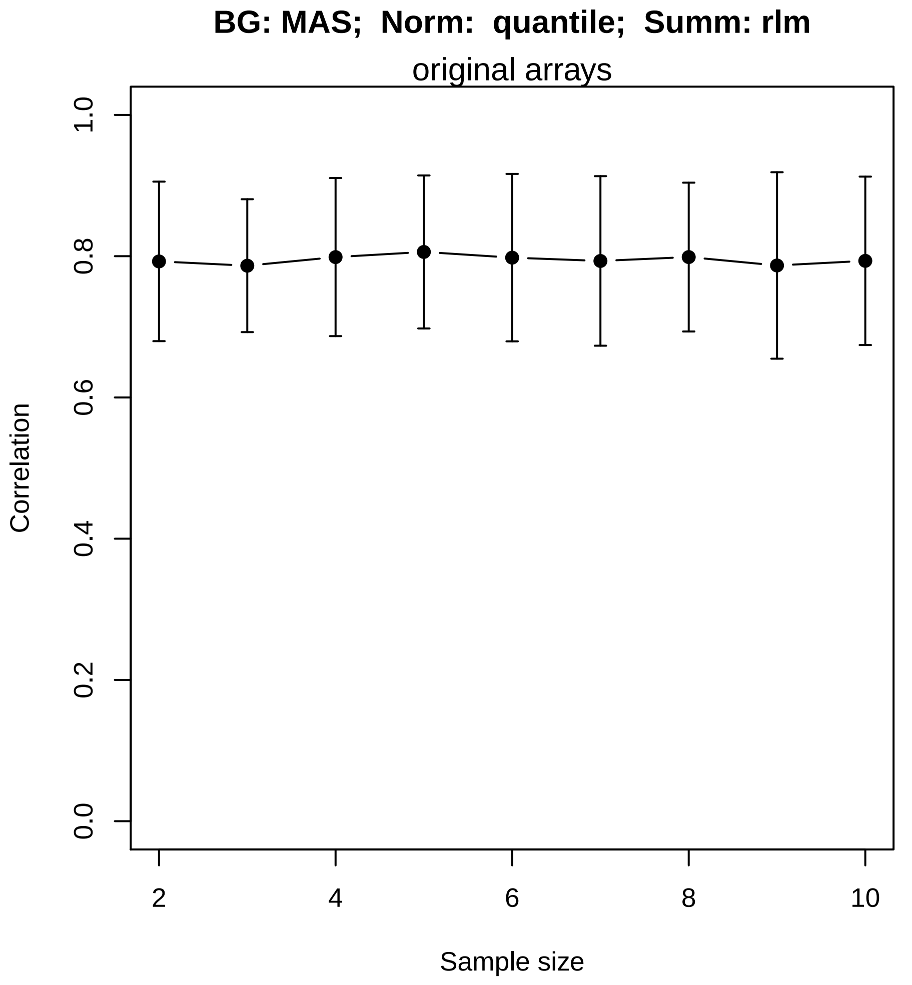

BG: MAS; Norm: quantile; Summ: rlm
original arrays
Correlation
Sample size

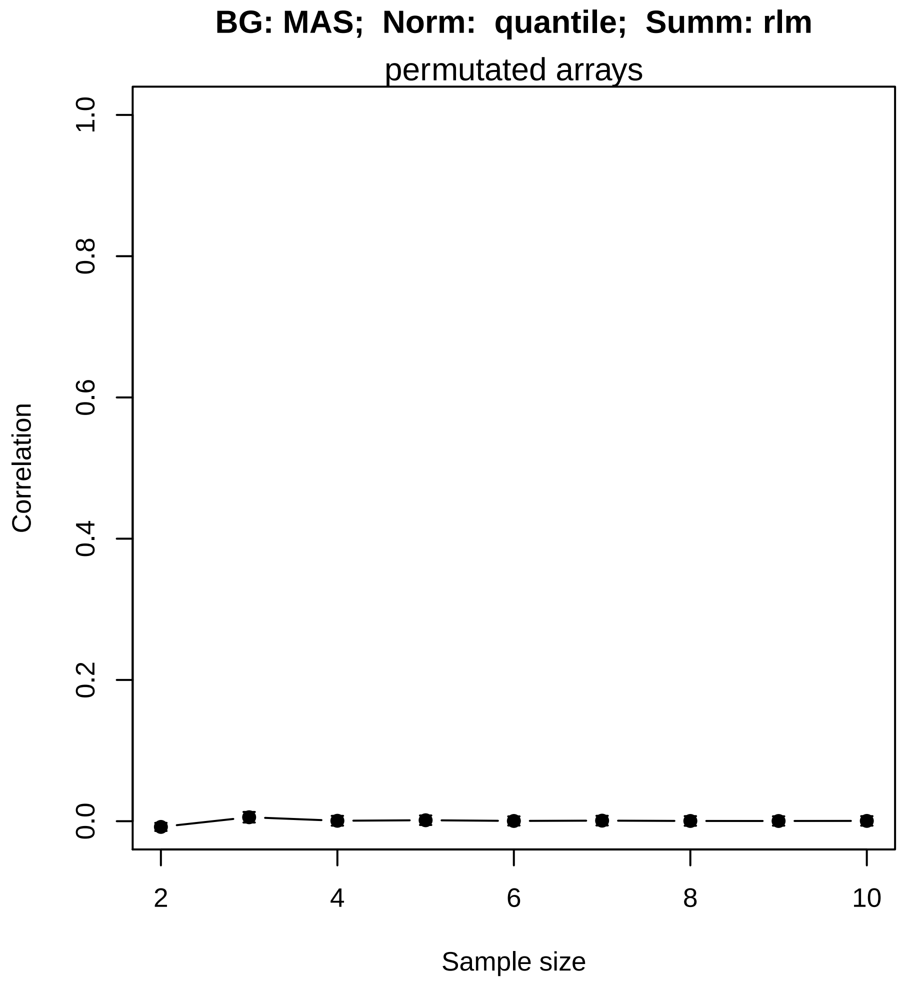

BG: MAS; Norm: quantile; Summ: rlm
permutated arrays
Correlation
Sample size

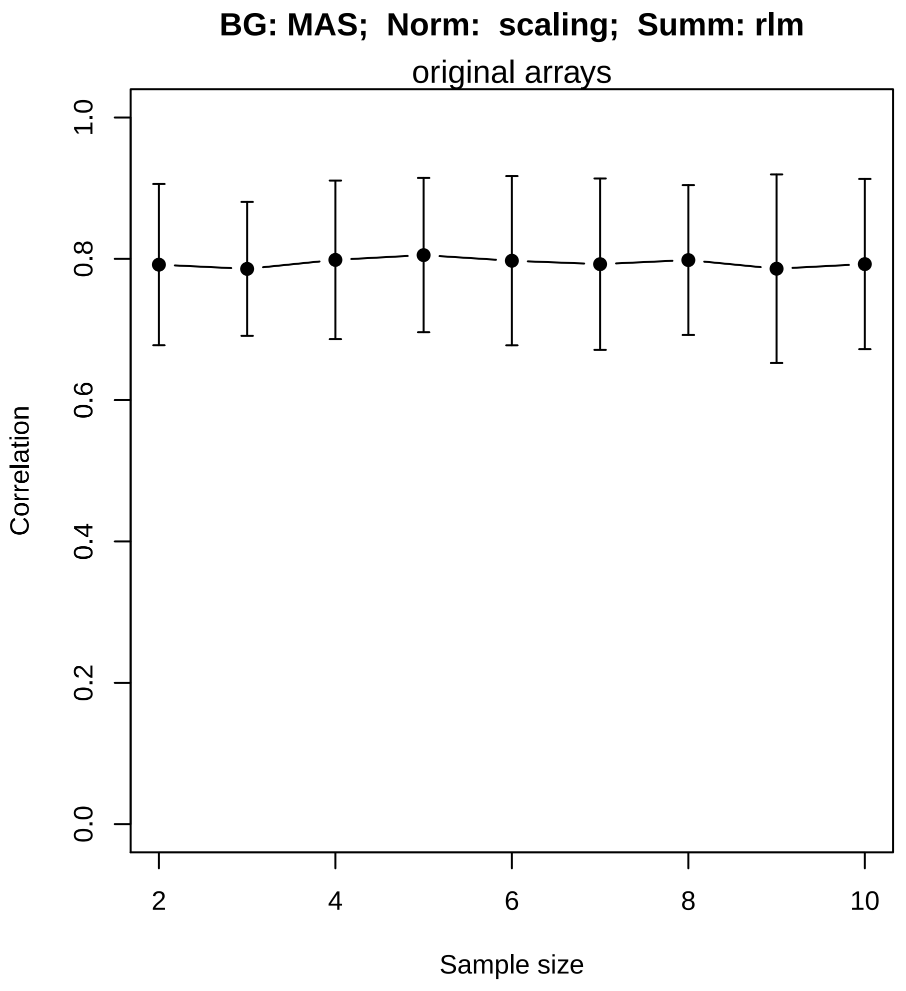
BG: MAS; Norm: scaling; Summ: rlm
original arrays
Correlation
0.0
0.2
0.4
0.6
0.8
1.0
2
4
6
8
10
Sample size

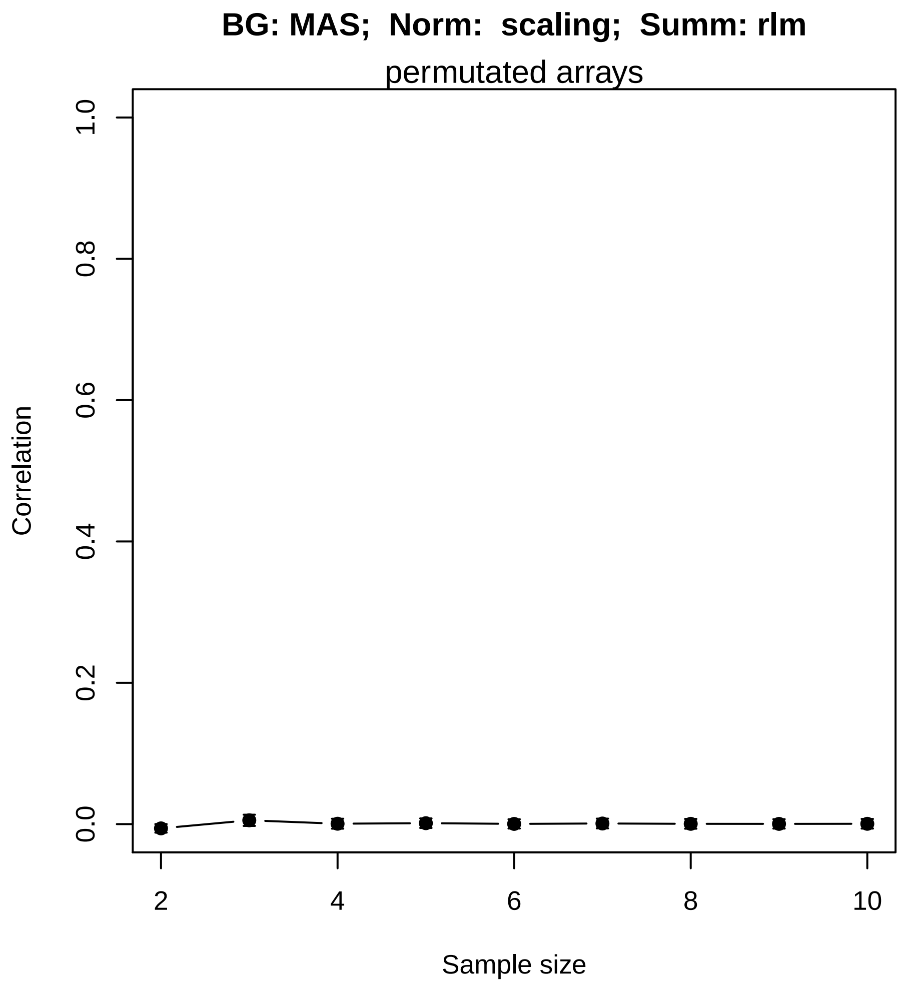
BG: MAS; Norm: scaling; Summ: rlm
permutated arrays
Correlation
0.0
0.2
0.4
0.6
0.8
1.0
2
4
6
8
10
Sample size

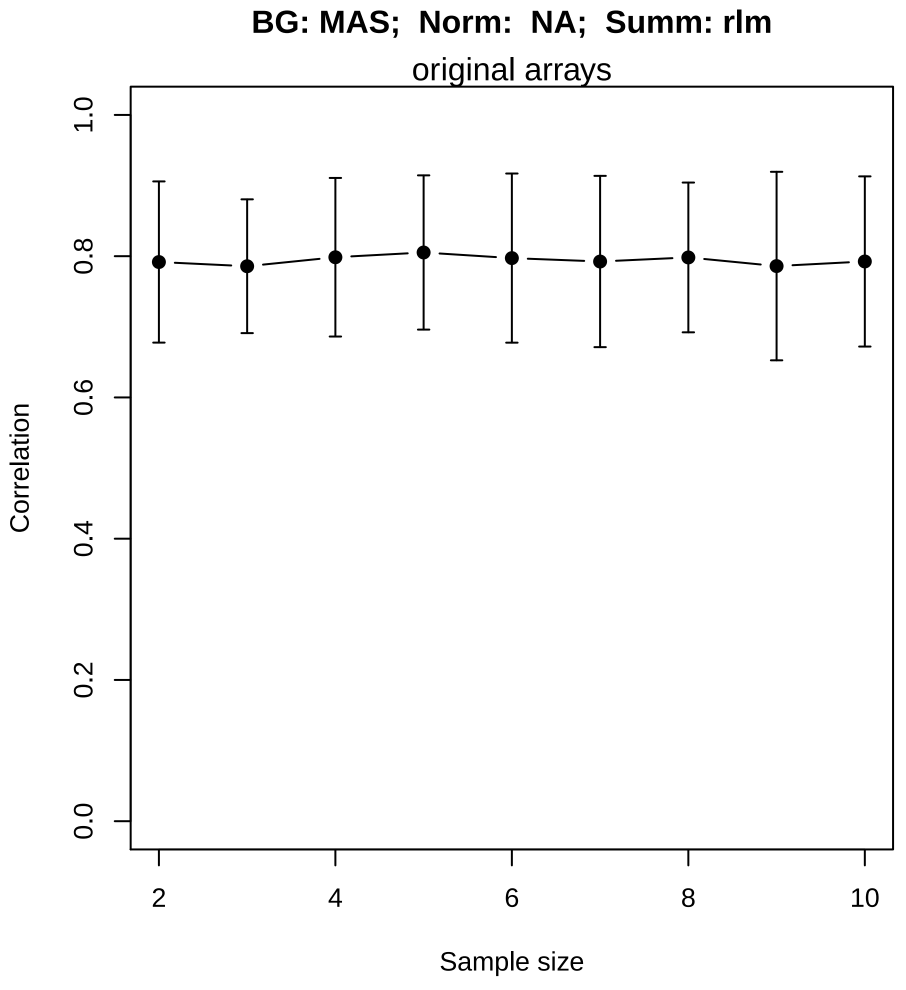
BG: MAS; Norm: NA; Summ: rlm
original arrays
Correlation
0.0
0.2
0.4
0.6
0.8
1.0
2
4
6
8
10
Sample size

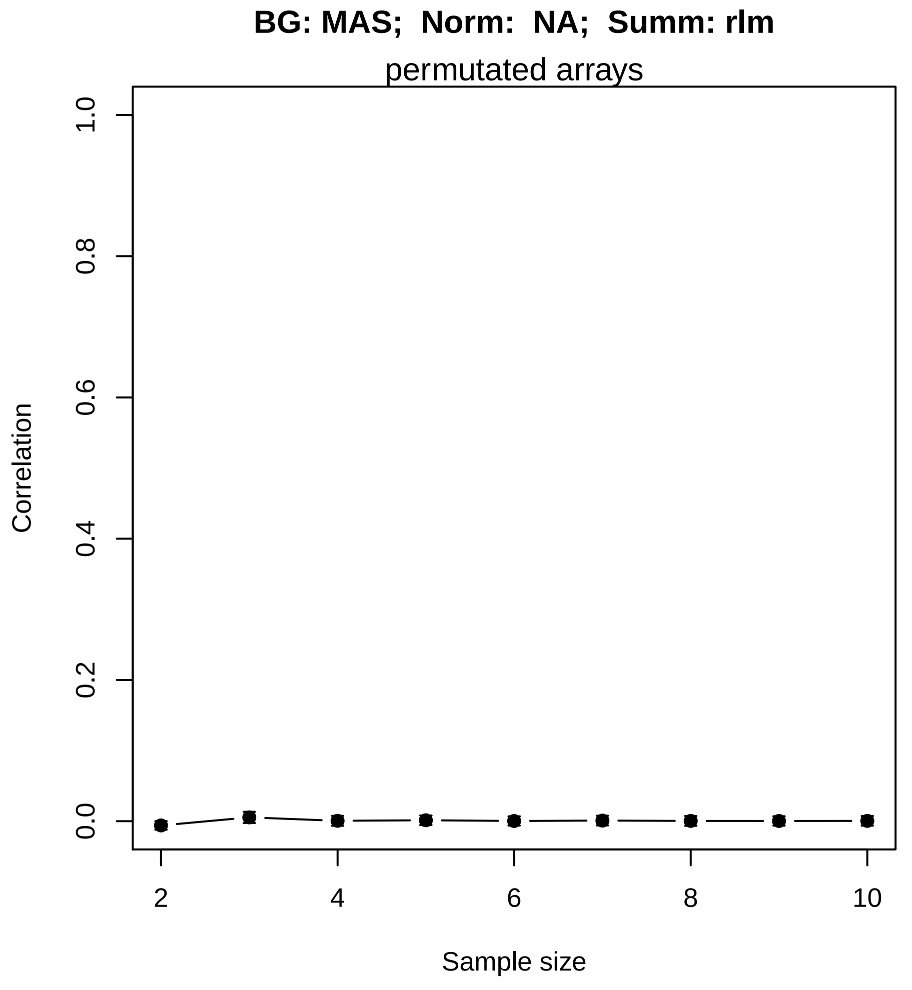
BG: MAS; Norm: NA; Summ: rlm
permutated arrays
Correlation
0.0
0.2
0.4
0.6
0.8
1.0
2
4
6
8
10
Sample size

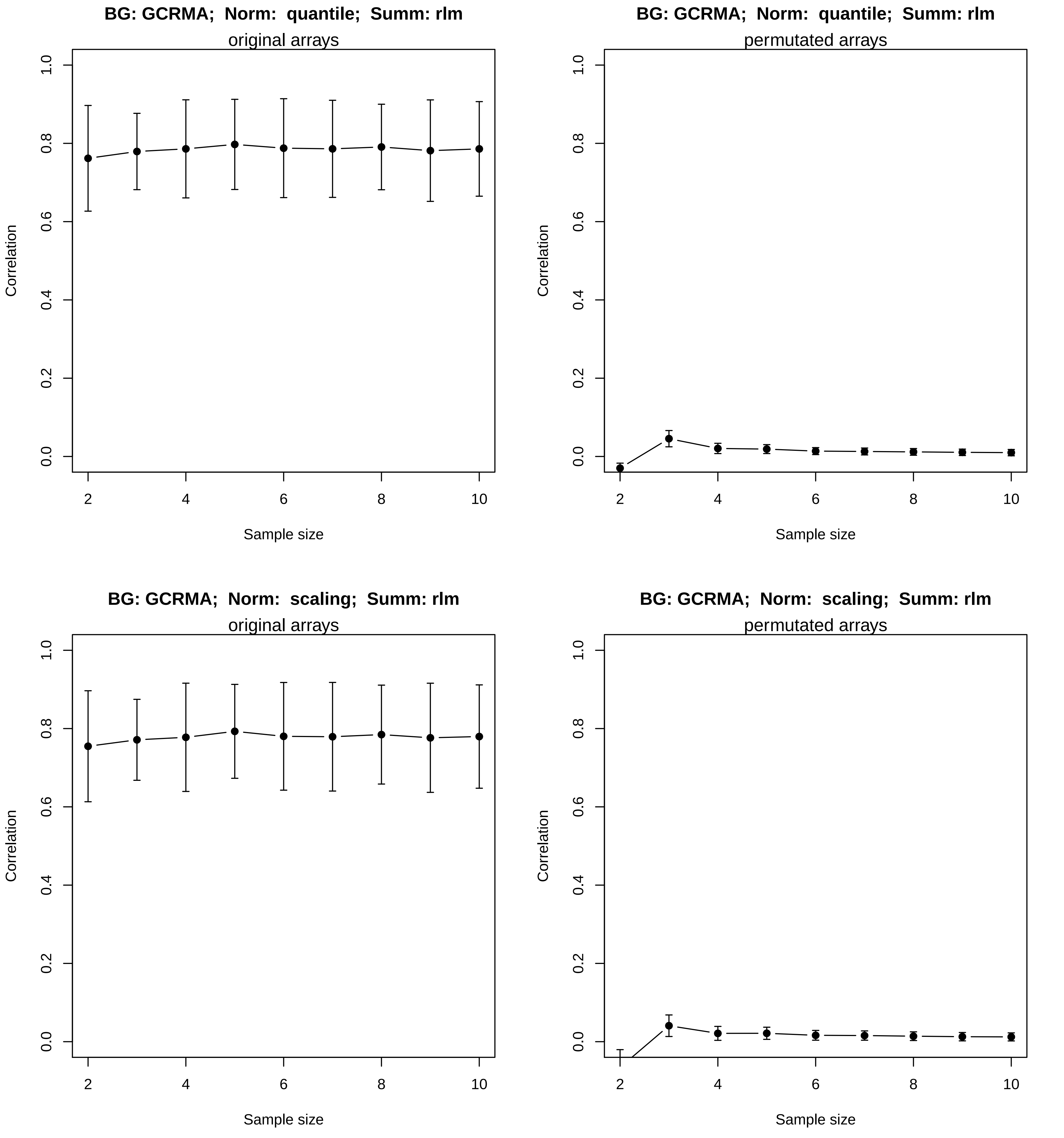
BG: GCRMA; Norm: quantile; Summ: rlm
original arrays
Correlation
Sample size
BG: GCRMA; Norm: quantile; Summ: rlm
permutated arrays
Correlation
Sample size
BG: GCRMA; Norm: scaling; Summ: rlm
original arrays
Correlation
Sample size
BG: GCRMA; Norm: scaling; Summ: rlm
permutated arrays
Correlation
Sample size

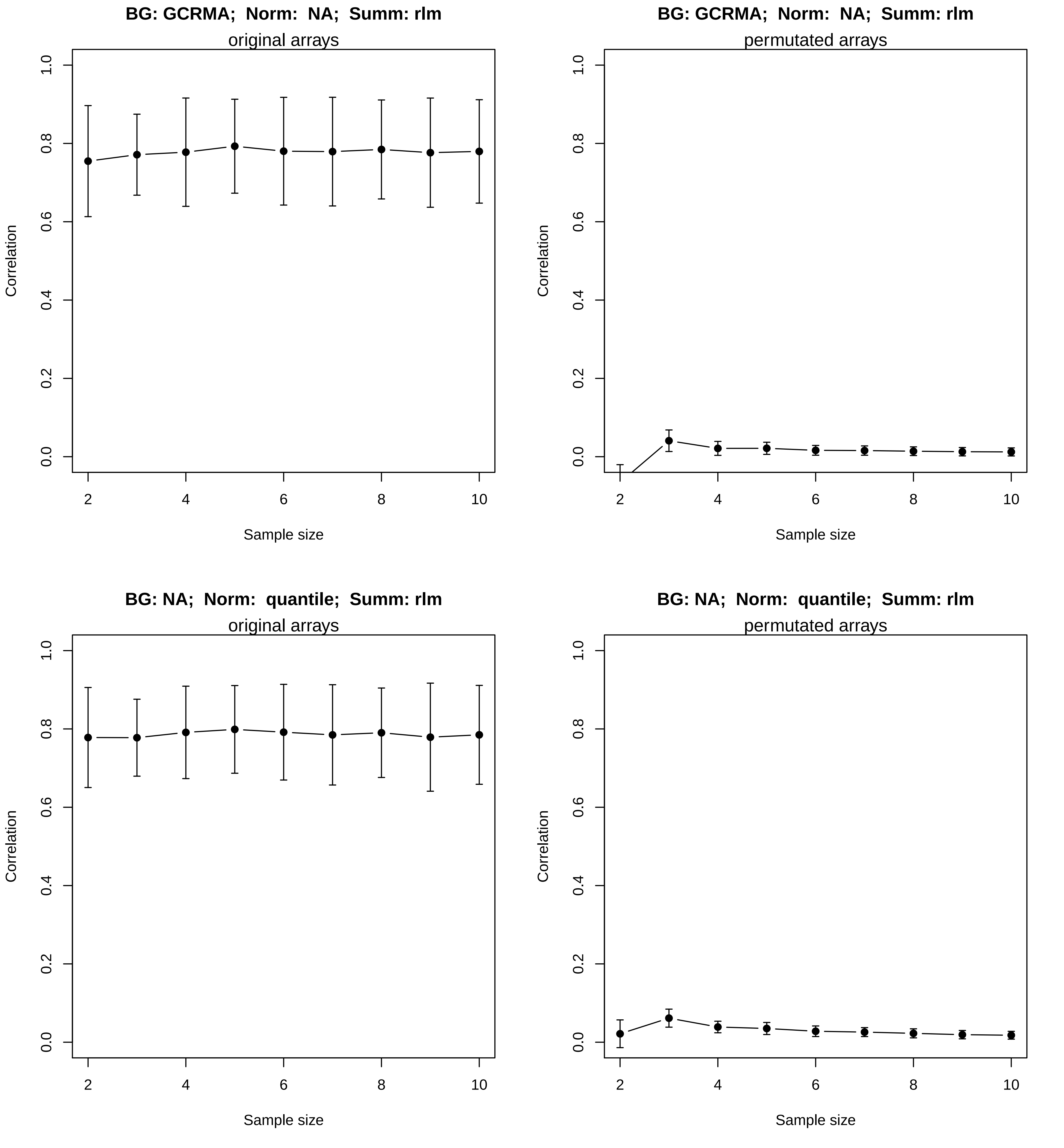

BG: GCRMA; Norm: NA; Summ: rlm
original arrays
Correlation
Sample size
BG: GCRMA; Norm: NA; Summ: rlm
permutated arrays
Correlation
Sample size
BG: NA; Norm: quantile; Summ: rlm
original arrays
Correlation
Sample size
BG: NA; Norm: quantile; Summ: rlm
permutated arrays
Correlation
Sample size

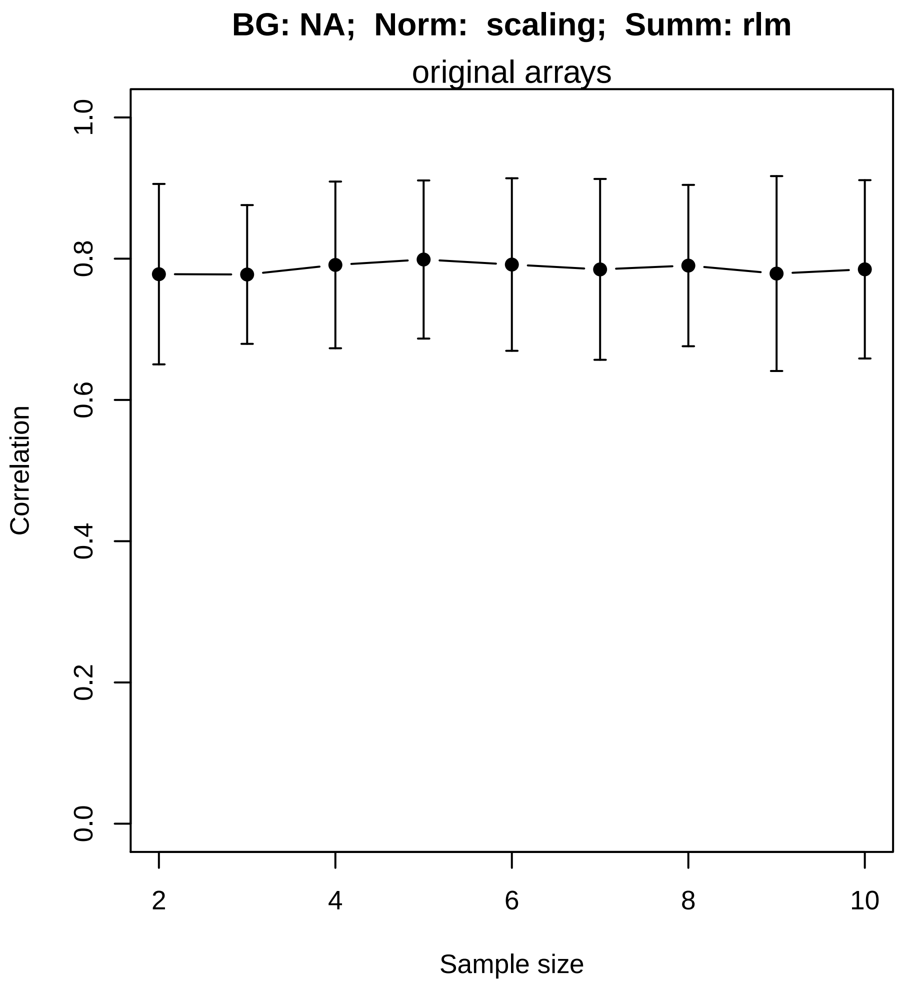
BG: NA; Norm: scaling; Summ: rlm
original arrays
Correlation
0.0
0.2
0.4
0.6
0.8
1.0
2
4
6
8
10
Sample size

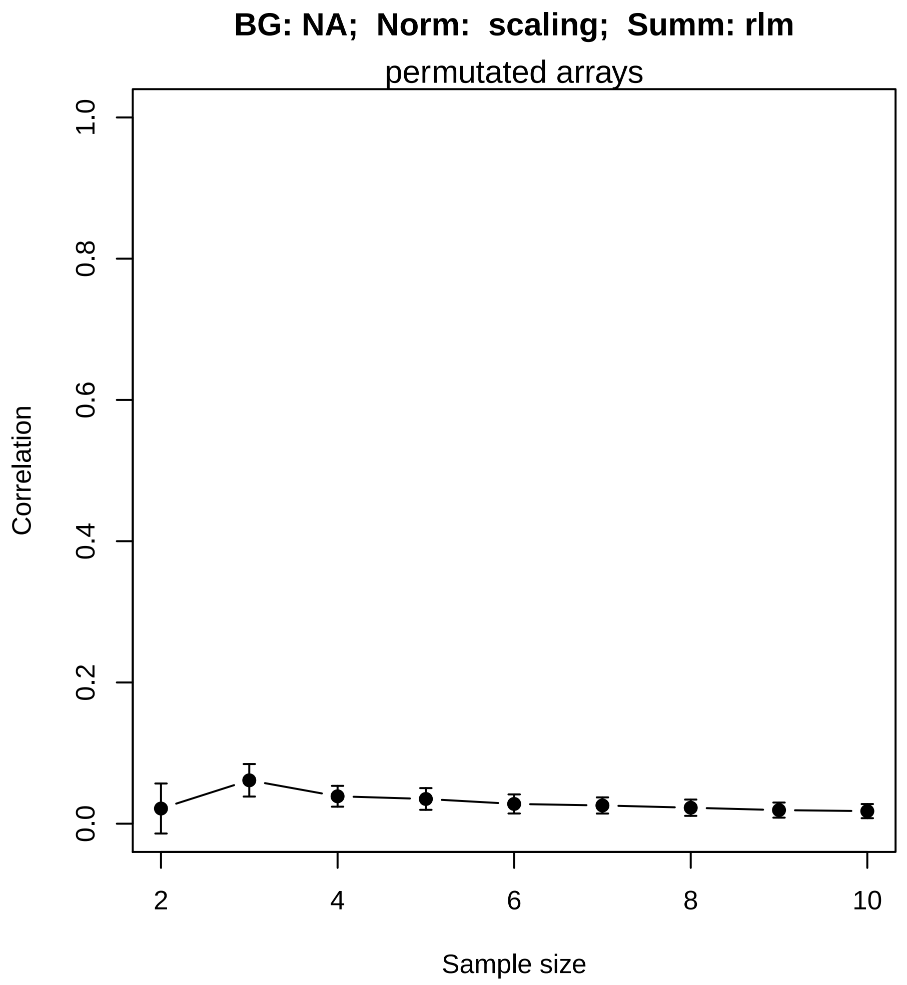
BG: NA; Norm: scaling; Summ: rlm
permutated arrays
Correlation
0.0
0.2
0.4
0.6
0.8
1.0
2
4
6
8
10
Sample size

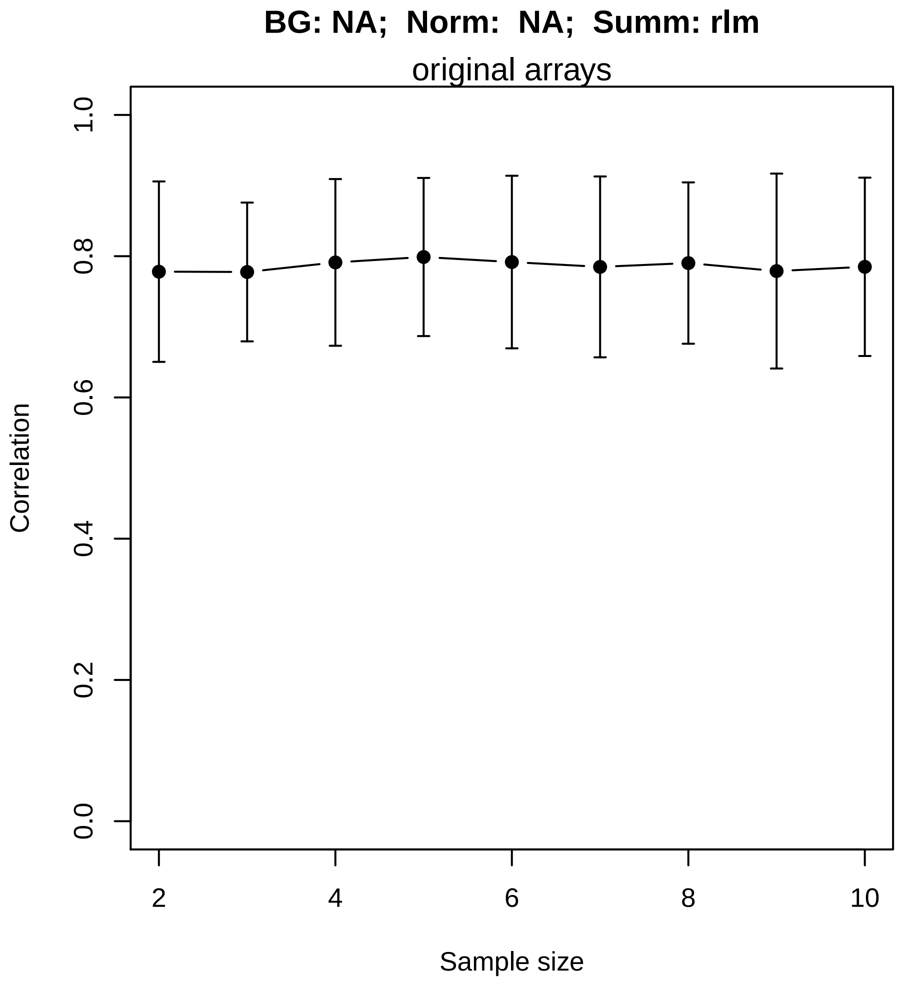
BG: NA; Norm: NA; Summ: rlm
original arrays
Correlation
0.0
0.2
0.4
0.6
0.8
1.0
2
4
6
8
10
Sample size

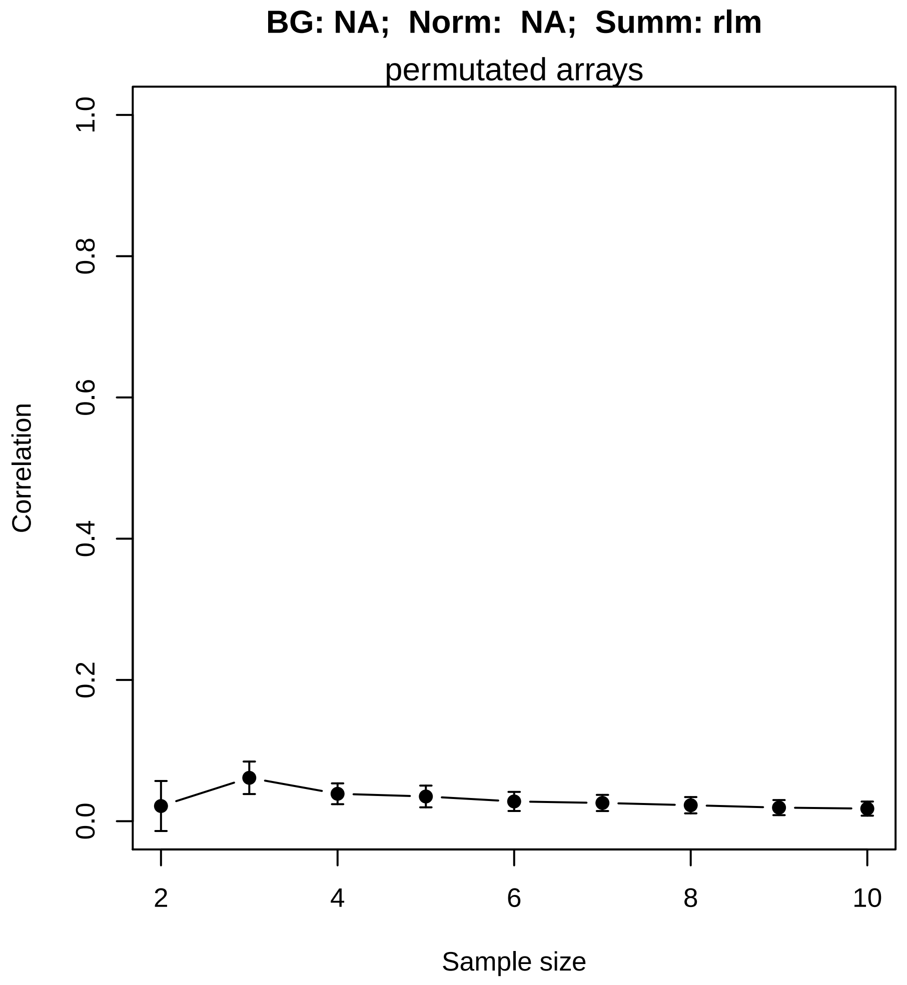
BG: NA; Norm: NA; Summ: rlm
permutated arrays
Correlation
0.0
0.2
0.4
0.6
0.8
1.0
2
4
6
8
10
Sample size

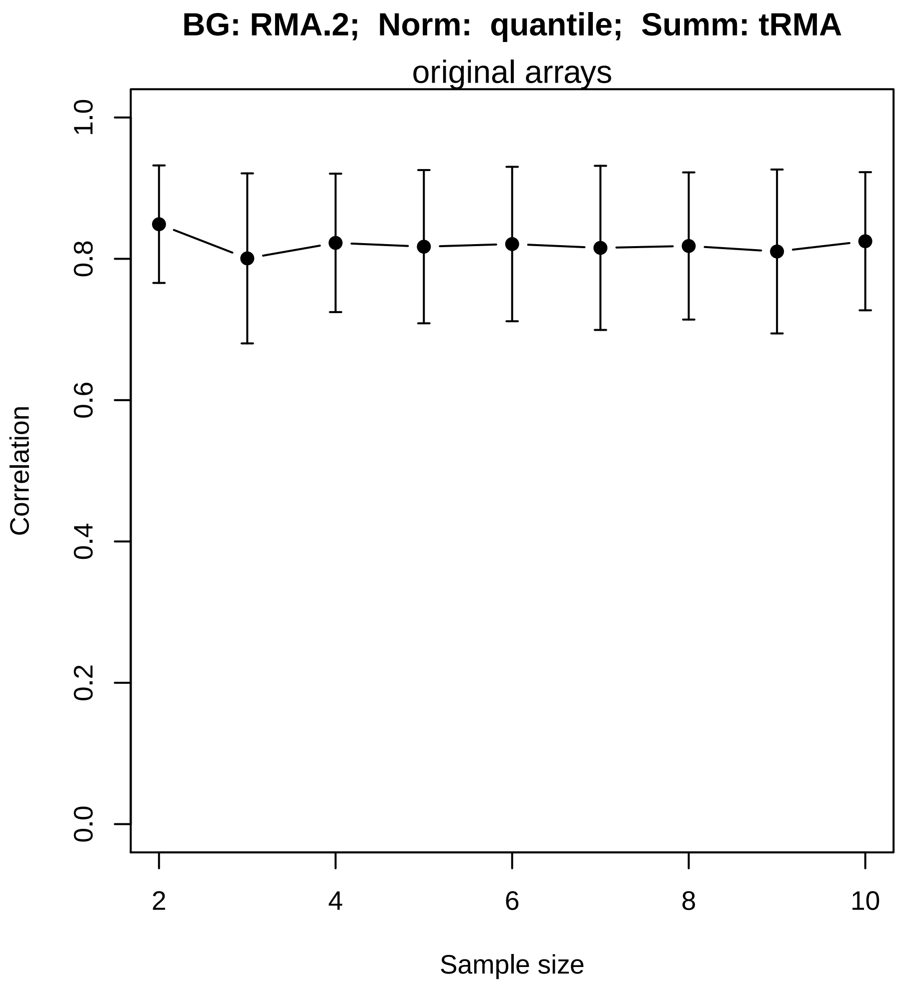
BG: RMA.2; Norm: quantile; Summ: tRMA
original arrays
Correlation
Sample size
0.0
0.2
0.4
0.6
0.8
1.0
2
4
6
8
10

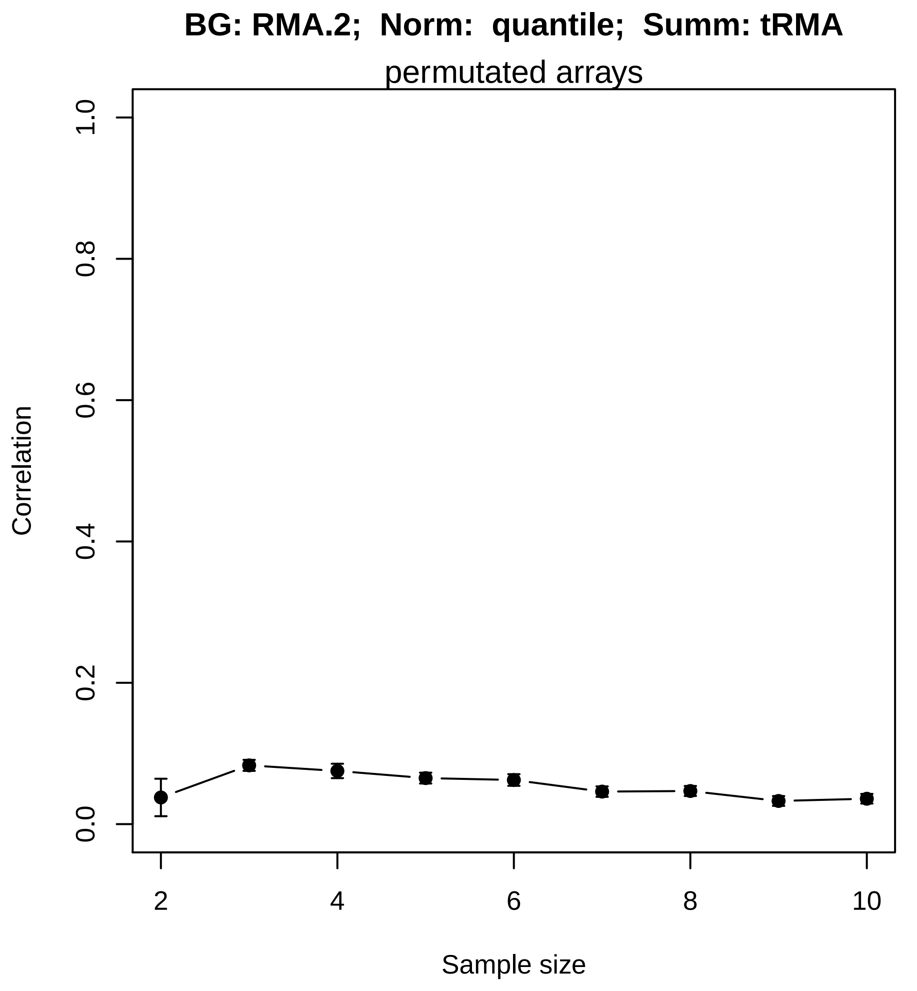
BG: RMA.2; Norm: quantile; Summ: tRMA
permutated arrays
Correlation
Sample size
0.0
0.2
0.4
0.6
0.8
1.0
2
4
6
8
10

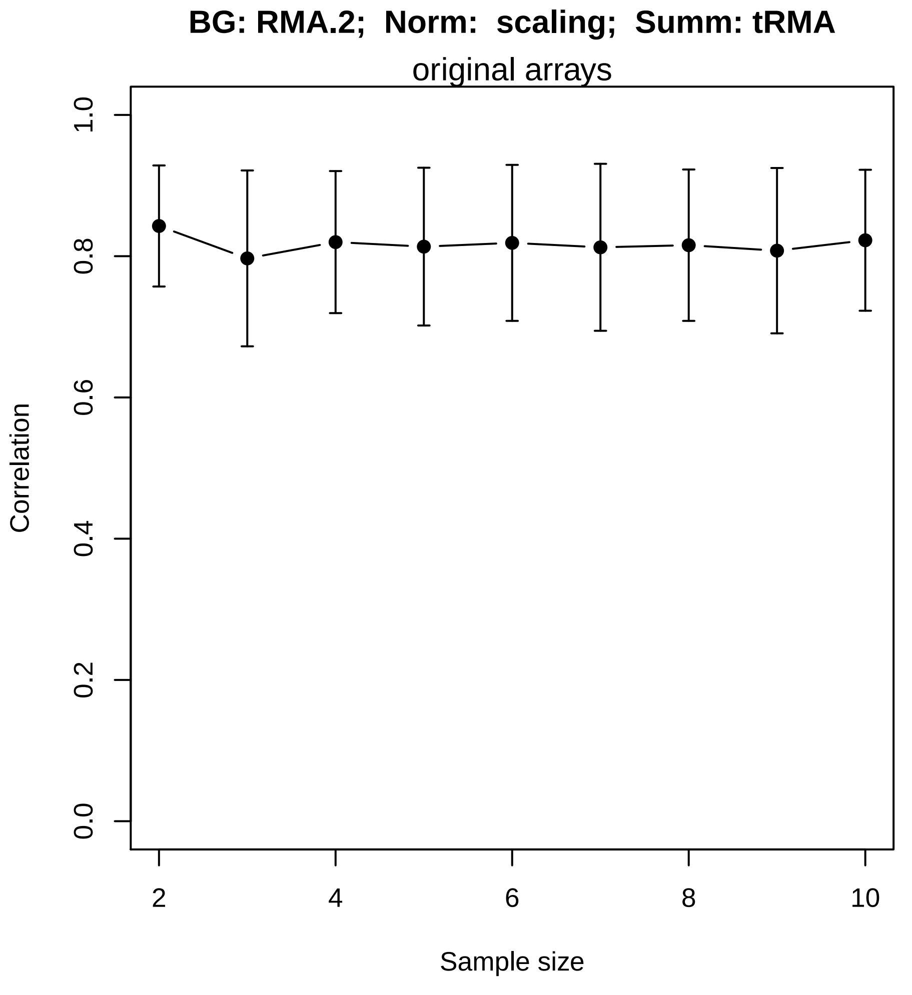
BG: RMA.2; Norm: scaling; Summ: tRMA
original arrays
Correlation
Sample size
0.0
0.2
0.4
0.6
0.8
1.0
2
4
6
8
10

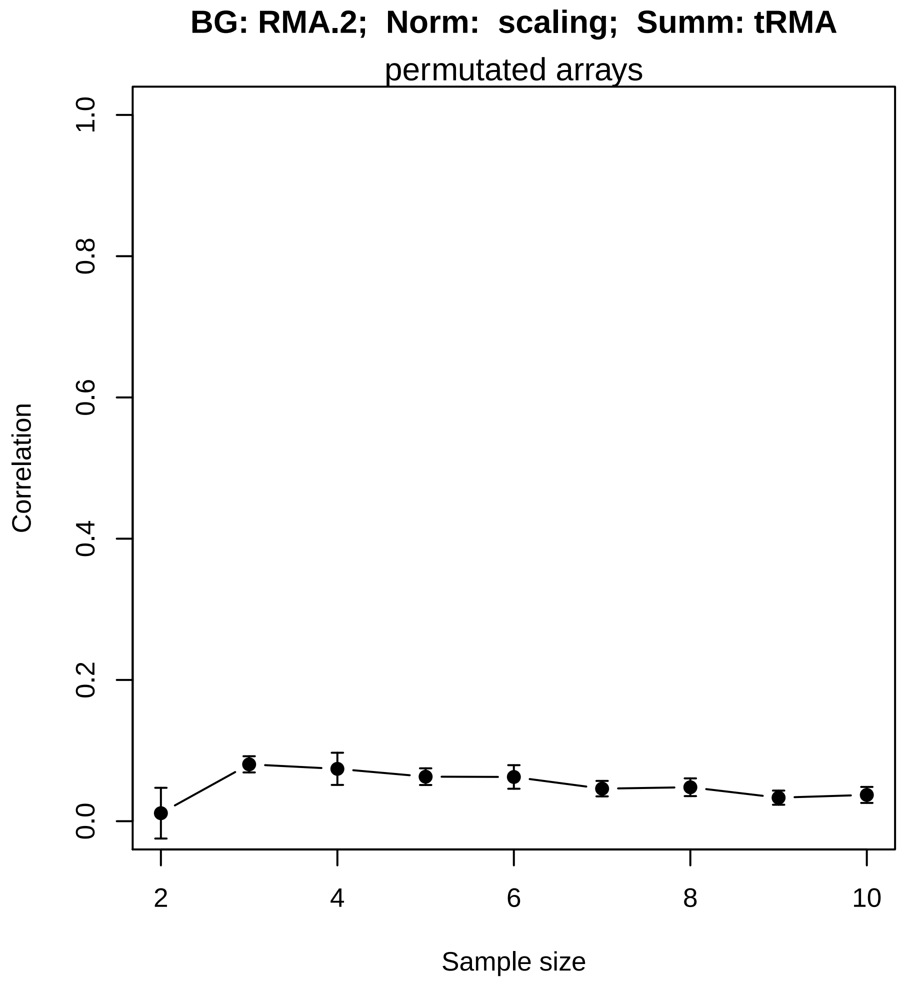
BG: RMA.2; Norm: scaling; Summ: tRMA
permutated arrays
Correlation
Sample size
0.0
0.2
0.4
0.6
0.8
1.0
2
4
6
8
10

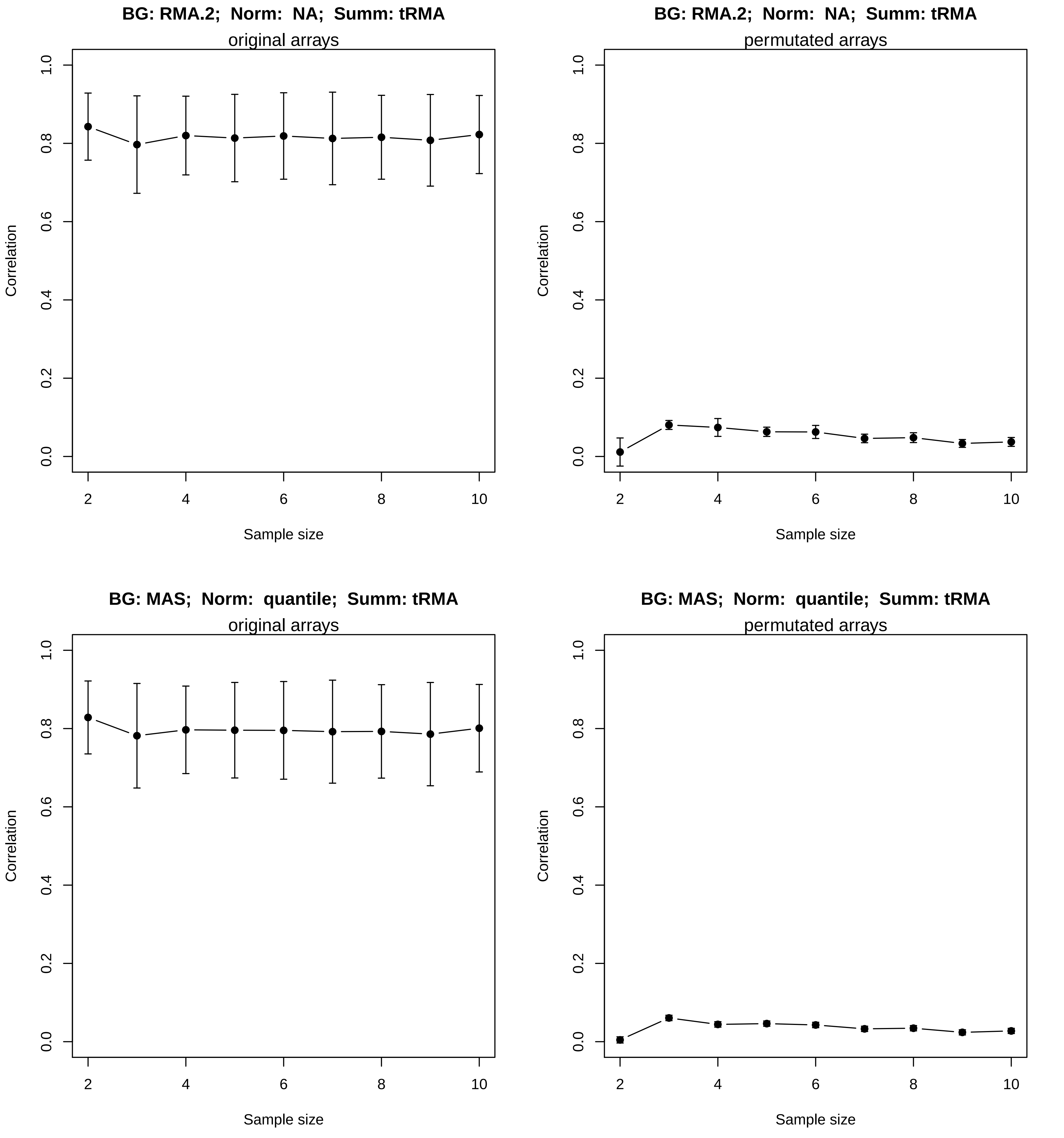
BG: RMA.2; Norm: NA; Summ: tRMA
original arrays
permutated arrays
BG: MAS; Norm: quantile; Summ: tRMA
original arrays
permutated arrays
Correlation
Sample size
0.0
0.2
0.4
0.6
0.8
1.0
2
4
6
8
10

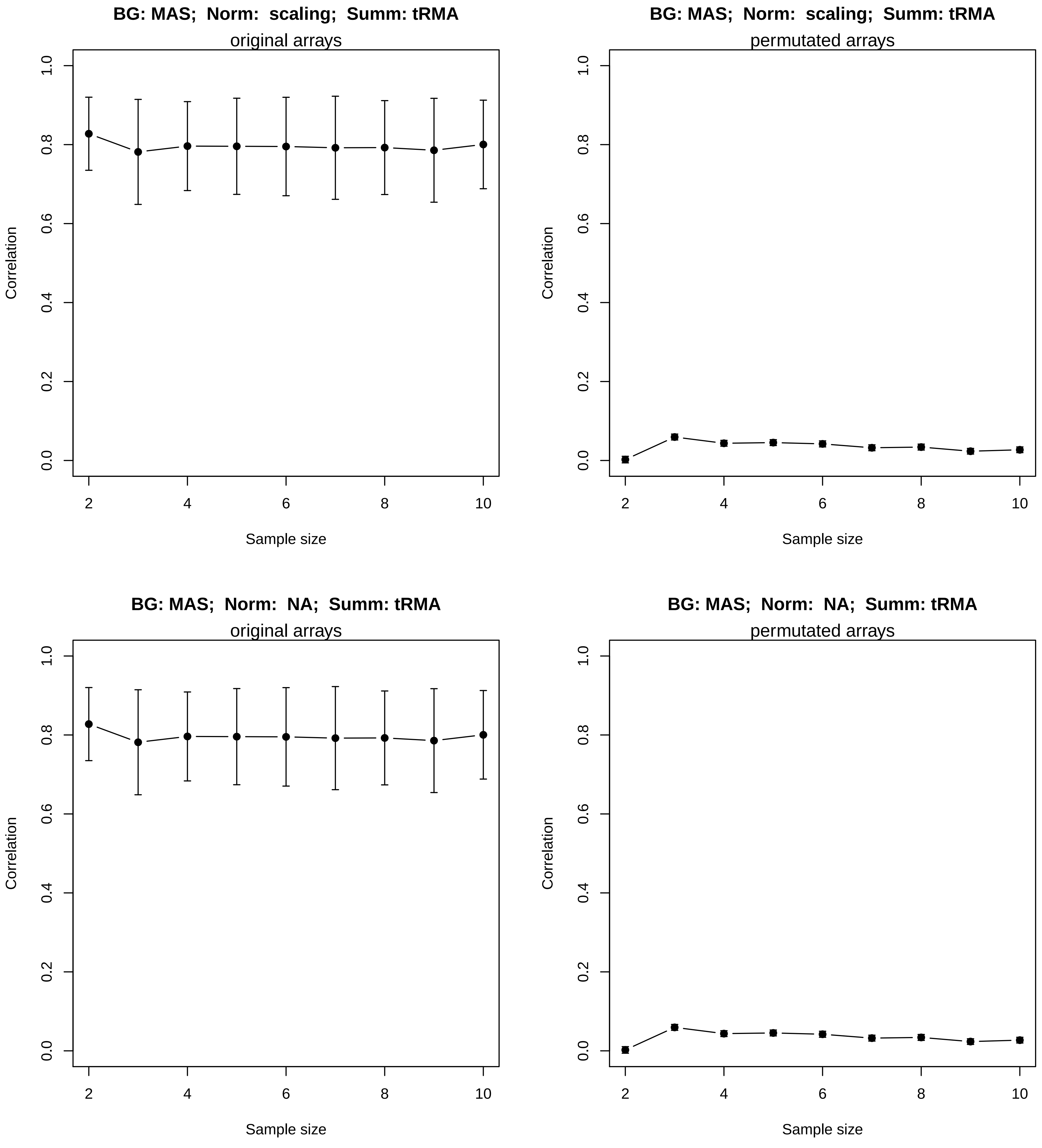

BG: MAS; Norm: scaling; Summ: tRMA
original arrays
BG: MAS; Norm: scaling; Summ: tRMA
permutated arrays
BG: MAS; Norm: NA; Summ: tRMA
original arrays
BG: MAS; Norm: NA; Summ: tRMA
permutated arrays
Correlation
Sample size
0.0
0.2
0.4
0.6
0.8
1.0
2
4
6
8
10

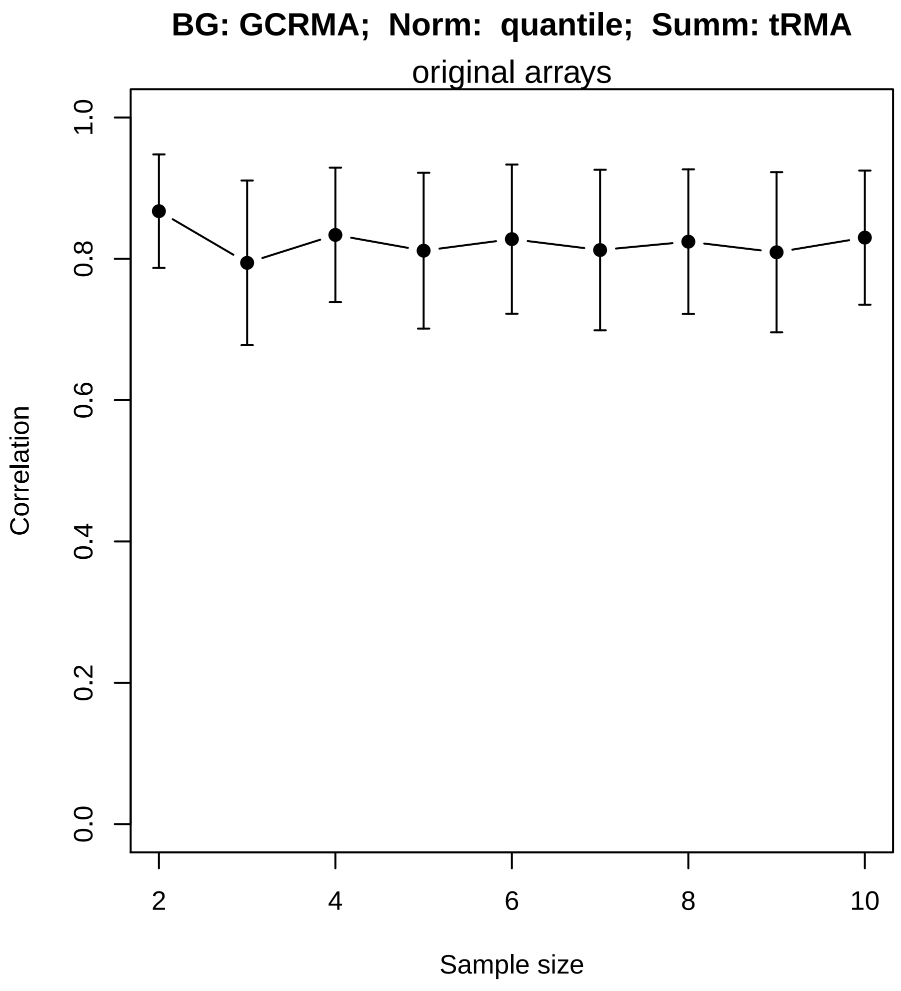
BG: GCRMA; Norm: quantile; Summ: tRMA
original arrays
Correlation
Sample size

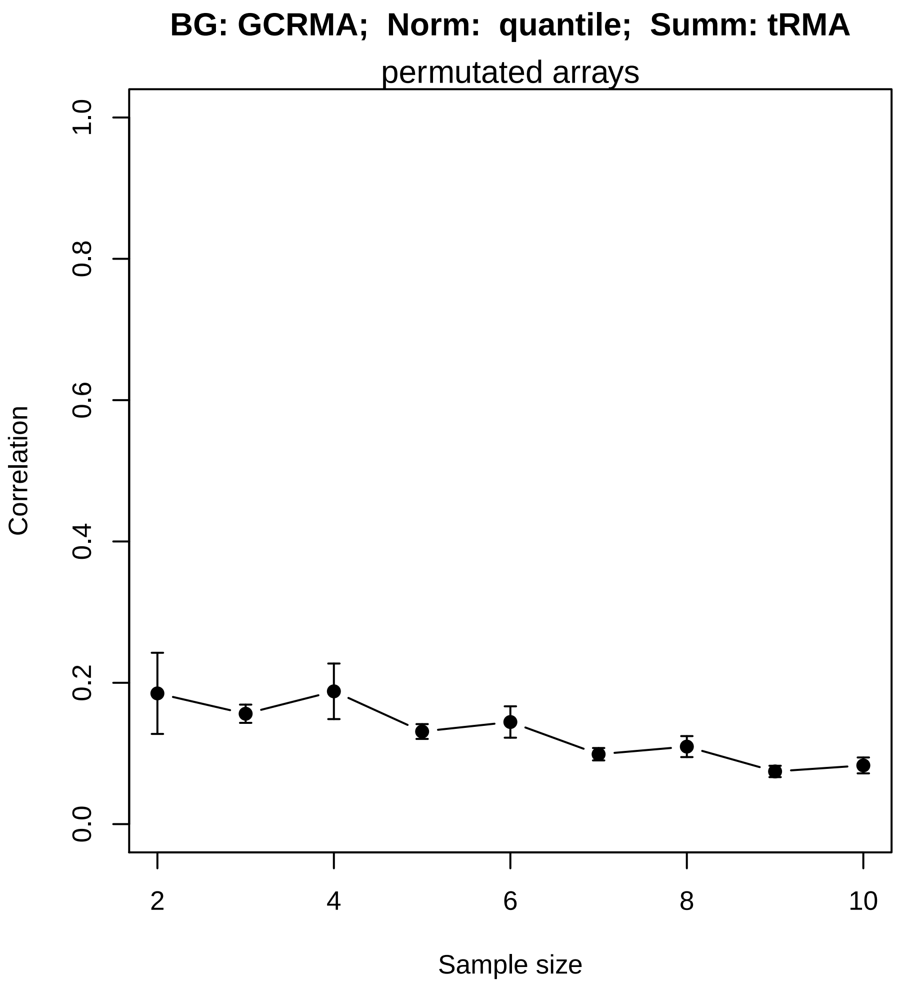
BG: GCRMA; Norm: quantile; Summ: tRMA
permutated arrays
Correlation
Sample size

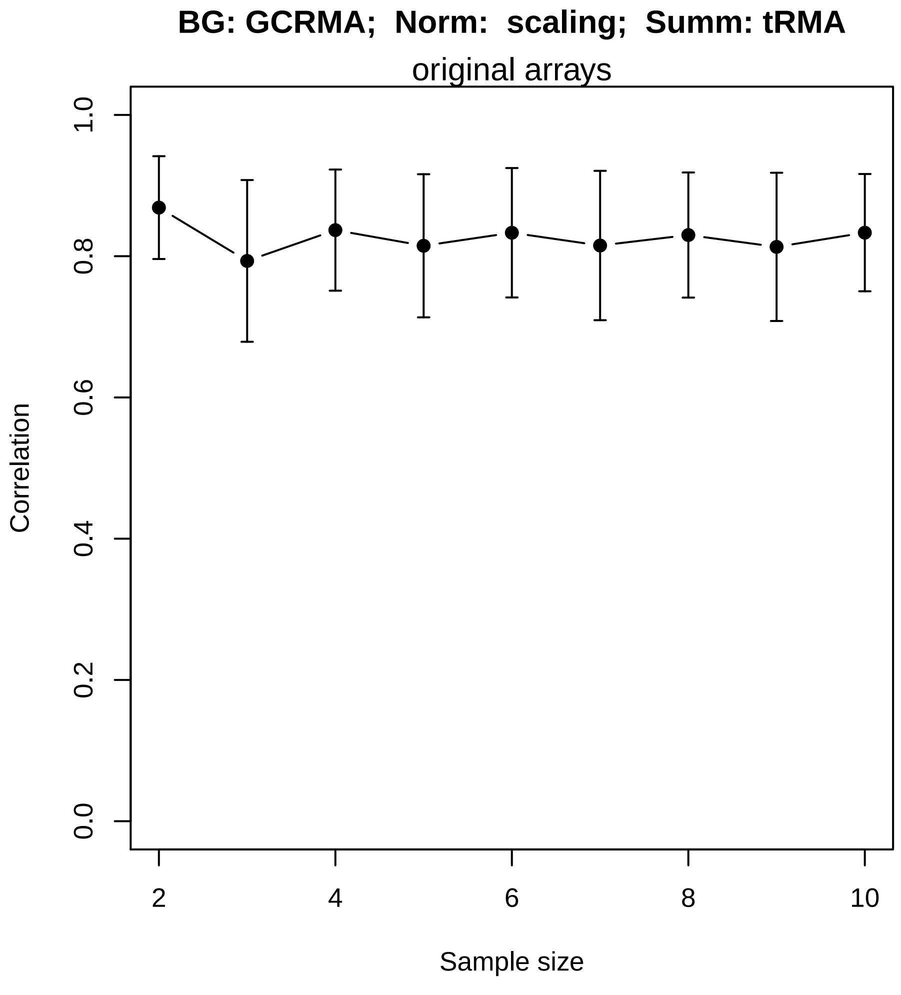
BG: GCRMA; Norm: scaling; Summ: tRMA
original arrays
Correlation
Sample size

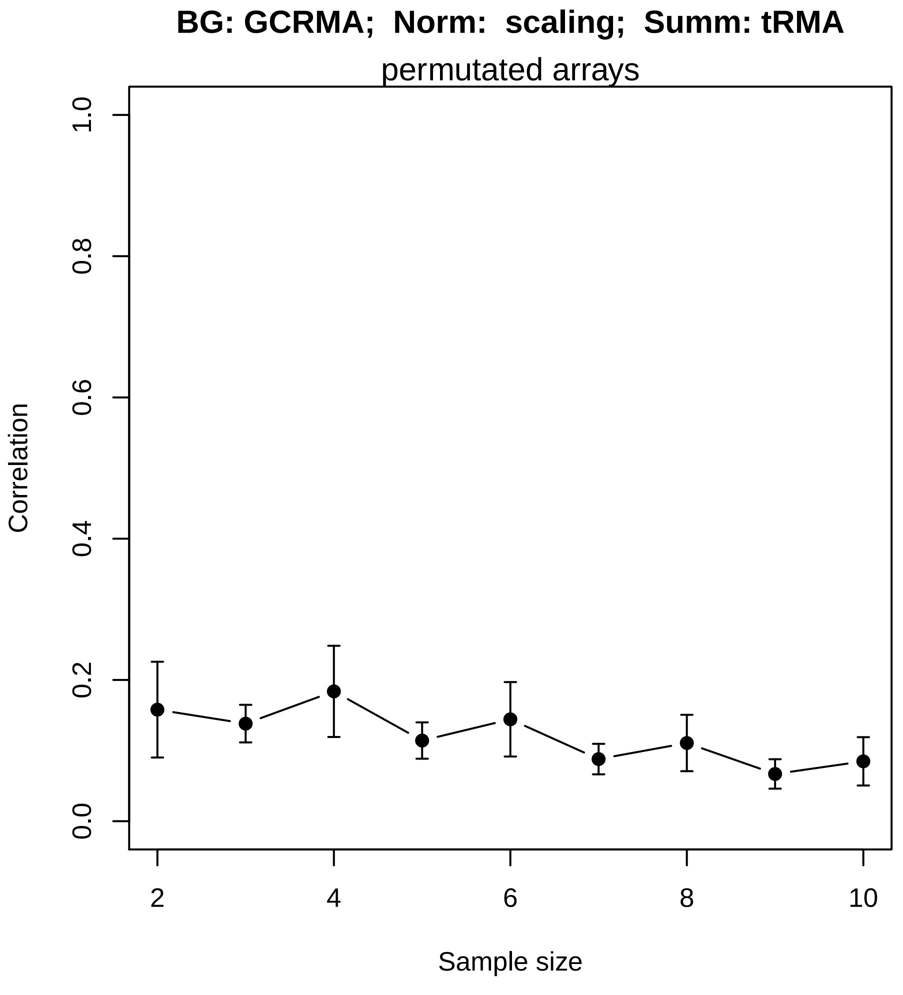
BG: GCRMA; Norm: scaling; Summ: tRMA
permutated arrays
Correlation
Sample size

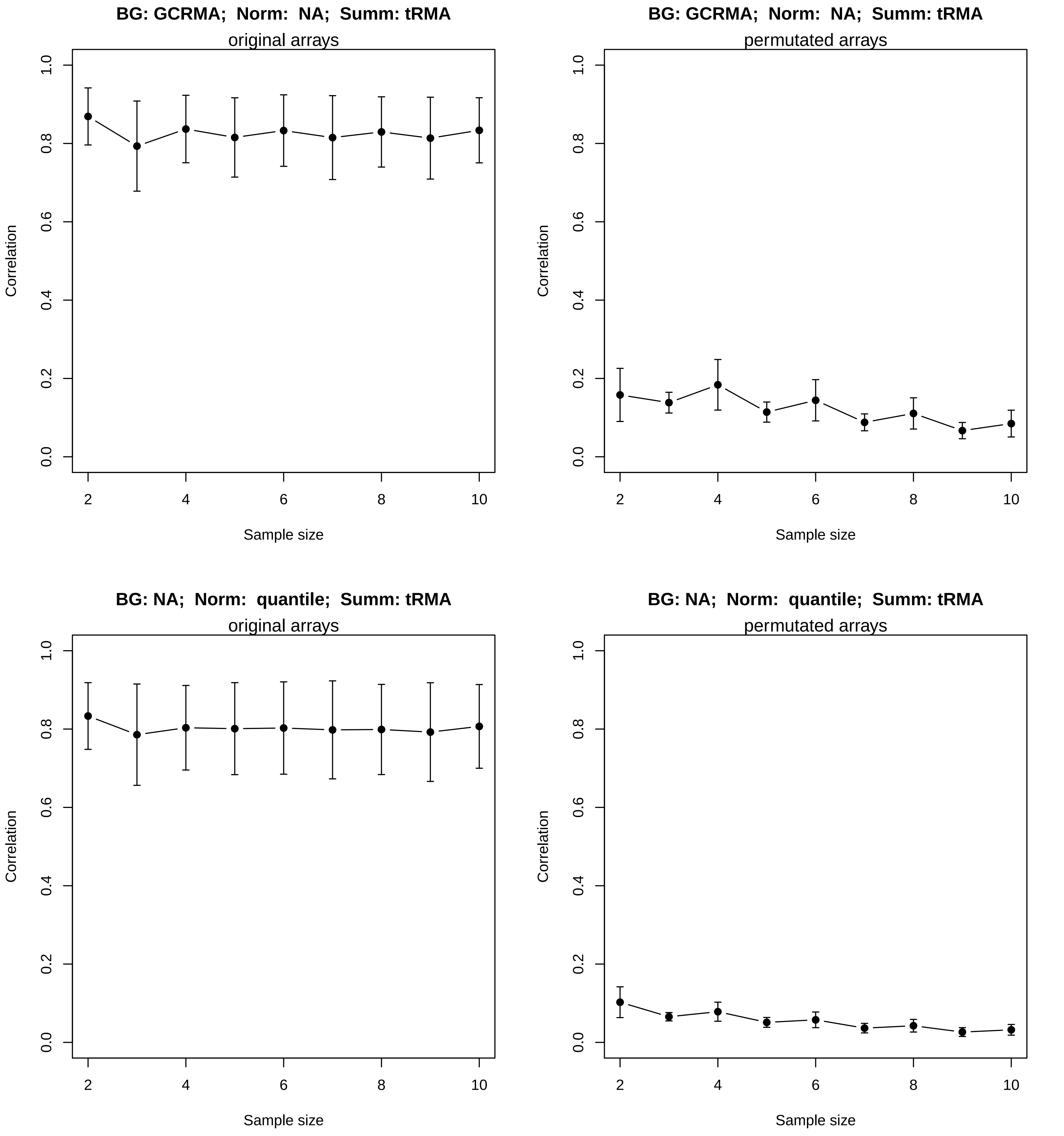
BG: GCRMA; Norm: NA; Summ: tRMA
original arrays
Correlation
Sample size
0.0
0.2
0.4
0.6
0.8
1.0
2
4
6
8
10
BG: GCRMA; Norm: NA; Summ: tRMA
permutated arrays
Correlation
Sample size
BG: NA; Norm: quantile; Summ: tRMA
original arrays
Correlation
Sample size
BG: NA; Norm: quantile; Summ: tRMA
permutated arrays
Correlation
Sample size

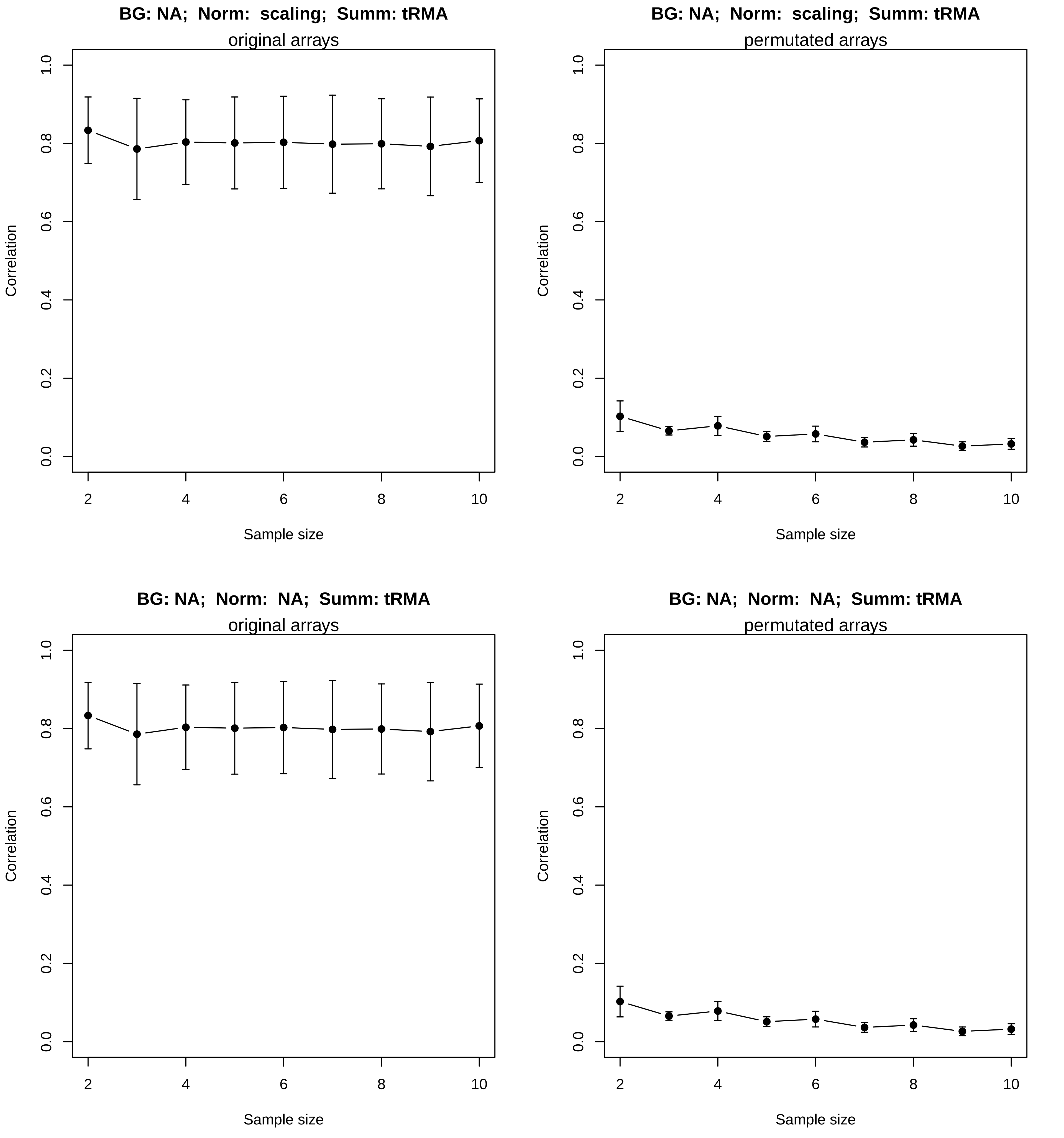
BG: NA; Norm: scaling; Summ: tRMA
original arrays
BG: NA; Norm: scaling; Summ: tRMA
permutated arrays
BG: NA; Norm: NA; Summ: tRMA
original arrays
BG: NA; Norm: NA; Summ: tRMA
permutated arrays
Correlation
0.0
0.2
0.4
0.6
0.8
1.0
Sample size
2
4
6
8
10
