# Supplementary material for: Algorithm-driven Artifacts in median polish summarization of Microarray data
Source: BMC Bioinformatics. 2010 Nov 11;11:553. doi: 10.1186/1471-2105-11-553 (PMC2998528; doi:10.1186/1471-2105-11-553)
Supplement: Additional file 8 — Figure S10. detail of the (t)RMA median polish procedures for a single probeset. Using alternating row and column sweeps, medians are calculated and subtracted until convergence is reached. In the case of RMA (upper panel), this can lead to the generation of many zeroes in a column, which subsequently could lead to the column effect also being zero, since the column effect is calculated as the median of the column residuals. In the case of tRMA (lower panel) zeroes introduced during a column sweep would lead to rather similar row (i.e. probe) effects. [file 1471-2105-11-553-S8.PDF]

RMA

B. Row sweep.
For each row, medians are calculated.

	A	B	C	→	
a	7	11	2	→	7
b	8	12	4	→	8
c	6	10	11	→	10
d	9	13	14	→	13
e	4	2	3	→	3

Then, medians are subtracted from each row.

	A	B	C		
a	0	4	-5		7
b	0	4	-4		8
c	-4	0	1		10
d	-4	0	1		13
e	1	-1	0		3

The grand effect is calculated by adding to the previous grand effect (0 if it's the first iteration) the median of row medians.

Grand effect = 0 + 8 = 8

Median of row medians is then subtracted from row medians and added to previous row effects (if present) to generate new row effects.

Row medians	-8 →	Row effects
7		-1
8		0
10		2
13		5
3		-5

C. Column sweep.
For each column, medians are calculated.

	A	B	C
a	0	4	-5
b	0	4	-4
c	-4	0	1
d	-4	0	1
e	1	-1	0
↓	0	0	0

Then, medians are subtracted from each column.

	A	B	C
a	0	4	-5
b	0	4	-4
c	-4	0	1
d	-4	0	1
e	1	-1	0
	0	0	0

The grand effect is calculated by adding to the previous grand effect the median of column medians.

Grand effect = 8 + 0 = 8

Median of column medians is then subtracted from column medians and added to previous column effects (if present) to generate new column effects.

0	0	0
↓ -0		
0	0	0

Column effects

D. Iterate until convergence.
Steps B and C are repeated until all column medians fall to zero (In the present case, this happens during the first iteration).
RMA will yield as output for each sample the final column effects plus the grand effect.

Final results - RMA

Grand effect = 8
Row effects = -1, 0, 2, 5, -5
Column effects = 0, 0, 0
Sample values = 8, 8, 8

A. Probeset Matrix.
Organize probes belonging to the same probeset in a matrix

Samples

Probes

	A	B	C
e	...	...	...
a	7	11	2
b	8	12	4
c	6	10	11
d	9	13	14
e	4	2	3
a	...	...	...

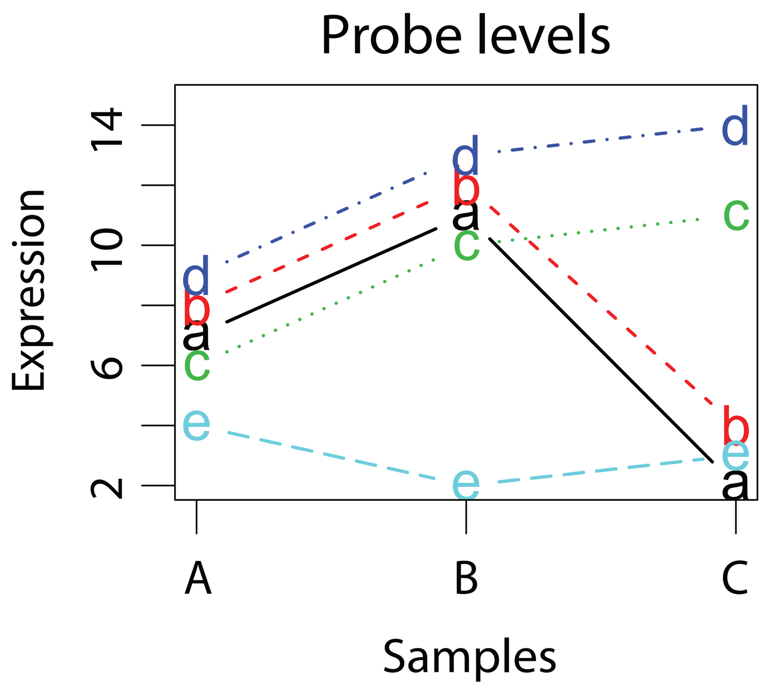

tRMA

B. Column sweep.
For each column, medians are calculated.

	A	B	C
a	7	11	2
b	8	12	4
c	6	10	11
d	9	13	14
e	4	2	3
↓	7	11	4

Then, medians are subtracted from each column.

	A	B	C
a	0	0	-2
b	1	1	0
c	-1	-1	7
d	2	2	10
e	-3	-9	-1
	7	11	4

The grand effect is calculated by adding to the previous grand effect (0 if it's the first iteration) the median of column medians.

Grand effect = 0 + 7 = 7

Median of column medians is then subtracted from column medians and added to previous column effects (if present) to generate new column effects.

7	11	4
↓ -7		
0	4	-3

Column effects

C. Row sweep.
For each row, medians are calculated.

	A	B	C	→	
a	0	0	-2	→	0
b	1	1	0	→	1
c	-1	-1	7	→	-1
d	2	2	10	→	2
e	-3	-9	-1	→	-3

Then, medians are subtracted from each row.

	A	B	C		
a	0	0	-4		0
b	0	0	-3		1
c	0	0	6		-1
d	0	0	6		2
e	0	-6	0		-3

The grand effect is calculated by adding to the previous grand effect the median of row medians.

Grand effect = 7 + 0 = 7

Median of row medians is then subtracted from row medians and added to previous row effects (if present) to generate new row effects.

Row medians	-0 →	Row effects
0		0
1		1
-1		-1
2		2
-3		-3

D. Iterate until convergence.
Steps B and C are repeated until all row medians fall to zero (In the present case, this happens during the second iteration).
tRMA will yield as output for each sample the final column effects plus the grand effect.

Final results - tRMA

Grand effect = 7
Row effects = 0, 1, -1, 2, -3
Column effects = 0, 4, -1
Sample values = 7, 11, 6
