# Supplementary material for: Algorithm-driven Artifacts in median polish summarization of Microarray data
Source: BMC Bioinformatics. 2010 Nov 11;11:553. doi: 10.1186/1471-2105-11-553 (PMC2998528; doi:10.1186/1471-2105-11-553)
Supplement: Additional file 13 — tRMA package for R (Windows), compatible with R.2.10.0 or later. R package for Windows of the trma procedure, where the median polish summarization step implements a "column-first" median calculation [file 1471-2105-11-553-S13.ZIP › trma/html/00Index.html]

R: What the package does (short line)

# What the package does (short line)

---

## Documentation for package ‘trma’ version 1.0

## Help Pages

|  |  |
| --- | --- |
| trma-package | transposed RMA |
| trma | transposed RMA |
| trma | transposed RMA |
